# COVID-19 vaccine confidence among adults of pima county using the NIMHD minority health and health disparities research framework: A qualitative analysis

**DOI:** 10.1371/journal.pone.0353345

**Published:** 2026-07-22

**Authors:** Kim Ellis, Pamela Hall, Latrisha Robinson, Sara Ruiz, Maiya G. Block Ngaybe, Christopher Lessenich, Namoonga M. Mantina, Carlos M. Perez-Velez, Beatrice Krauss, Purnima Madhivanan

**Affiliations:** 1 Mel and Enid Zuckerman College of Public Health, University of Arizona, Tucson, Arizona, United States of America; 2 Department of Psychology, College of Arts and Sciences, Barry University, Miami Shores, Florida, United States of America; 3 Department of Psychology, College of Arts, Sciences & Education, Florida International University, Miami, Florida, United States of America; 4 St. Jude Medical Center, Fullerton, California, United States of America; 5 University of Arizona Cancer Center, University of Arizona, Tucson, Arizona, United States of America; 6 Division of Infectious Diseases, College of Medicine, University of Arizona, Tucson, Arizona, United States of America; 7 Division of Epidemiology, Pima County Health Department, Tucson, Arizona, United States of America; 8 Psychology Department, College of Science, University of Arizona, Tucson, Arizona, United States of America; 9 Public Health Research Institute of India, Mysore, India; Central Laboratory for Evaluation of Veterinary Biologics, Agricultural Research Center, EGYPT

## Abstract

**Introduction:**

Vaccinations have been established as one of the most effective strategies to prevent the spread of infectious diseases. However, vaccine hesitancy has been a growing issue that needs to be monitored and understood to ensure successful promotion. Historic structural, social, and health disparities have been demonstrated to have significant impact on many health outcomes including vaccination uptake. This study applied the National Institute on Minority Health and Health Disparities Research Framework to examine factors influencing COVID-19 vaccination intention during the COVID-19 pandemic among adults in Pima County, Arizona.

**Methods:**

Between 22 Aug and 05 Dec 2020, 10 focus group discussions stratified by race/ethnicity, gender and language spoken (FGDs) were conducted virtually among 56 adult residents of Pima County, Arizona. After transcription and translation of FGDs conducted in Spanish, transcripts were organized by demographic characteristics and coded in Dedoose software by four independent coders. The NIMHD Framework’s Domains of Influence were used as *a priori* codes in the thematic analysis. All participants completed an informed consent process prior to participation.

**Results:**

Of the 56 participants who took part in the ten focus groups, 27% self-reported being Hispanic or Latinx, 25% were White/non-Hispanic, 9% were Black, and 4% were Asian. The preferred language was most often reported as English. Unique challenges for each demographic group emerged from this analysis, such as language barriers among Hispanic participants, historical mistreatment concerns among Black participants, and conflicting political messages for White participants. Cultural norms, family influences, and attitudes toward autonomy versus community responsibility further shaped vaccine decisions. Effective communication about vaccine safety, clinical trials, and public health messaging were identified as critical to addressing mistrust and vaccine confidence across diverse groups.

**Conclusion:**

Major themes in this study that predict vaccine intention include health literacy challenges, misinformation, mistrust in healthcare systems, and the desire to protect others. Understanding the behaviors and decisions behind vaccination acceptance and receipt is important for developing effective vaccine communication strategies. Identifying health disparities driving vaccination hesitancy may help guide future strategies and policies to improve vaccination rates, especially among minority groups.

## Introduction

The World Health Organization (WHO) recognized vaccine hesitancy as one of the top ten global health threats of 2019 since inadequate vaccination rates allow a disease to spread and create new variants [1]. In recent years, COVID-19 has been listed as the third leading cause of death in the United States (US), resulting in more than 6.5 million hospitalizations and about 1.2 million deaths [2].

Despite high morbidity and mortality rates, acceptance of COVID-19 vaccine remained low after its initial release. The COVID-19 vaccination program was launched on December 14^th^, 2020, in the US [3]. Ndugga et al found that 48% of Caucasians had received the vaccine by July 2021, while only 38% of Blacks and 41% of Hispanics had received the vaccine [4]. A study by Nikolovski et al found that 91% of adults aged 65 and older were willing to get vaccinated against COVID-19 [5], and Gatwood et al found an acceptance rate of 45.9% in adults aged 18–64 [6]. A review found that COVID-19 vaccination rates in the US among various populations ranged from a low of 12% to a high of 91% [7]. The same paper also mentioned that Arizona had recorded one of the highest rates (76%) of COVID-19 vaccine acceptance in the USA [7].

The Arizona Department of Health Services (ADHS) noted that the vaccine was available to all adults in Arizona (AZ) in December, 2020 [8]. By April, 2021, about 40% had received at least one dose [8], higher than the national average rate of 29.5% at the time [9]. Disparities among minority races and ethnicities in COVID-19 vaccination coverage persisted nationwide in vaccine rates [10] as well as in Arizona [11].

Multiple factors have been found to be associated with an individual’s decision to accept, refuse, or hesitate to get vaccinated, including religion, cultural background, socioeconomic status, political beliefs, the nature of vaccine communication, media consumption, past experiences with vaccination, sociodemographic characteristics and perceived benefits and risks [12–14]. Communities of Color (CoC) have been shown to face disproportionate rates of infection and death due to COVID-19 [15]. Vaccine hesitancy rates have been shown to be the highest among minority groups in the US [6,16,17]. For example, Ratnayake et al, found that Blacks had double the likelihood of vaccine hesitancy compared to Whites [14]. Similar results were reported by Savoia et al, with rates of vaccine hesitancy among Blacks being 70% in comparison to 22% for Whites [17]. A study by Williams et al, revealed that vaccination coverage is lower among all racial and ethnic minority groups except Asians when compared to Whites [18]. It has also been shown that Hispanics and Blacks had the highest rate of participants that were undecided about vaccination or less likely to be fully vaccinated [4,16,18]. While Savoia et al found similar results, their results differed in that they found that Hispanics were slightly less likely to be vaccine hesitant when compared to other races [17]. Though White communities generally fare better across many health measures compared to other racial and ethnic groups, disparities still exist in White communities. Several social determinants of health that impact White communities include socioeconomic status, neighborhood, and built environment [4].

In this paper, we analyzed COVID-19 vaccine decision-making at the time when the vaccine was in the initial stages of its rollout. Relying on a health disparities research framework, this qualitative study examined the decisions that impacted vaccine receipt, acceptance, and hesitancy in CoC and White communities in Pima County, AZ.

## Methods

### Participants

The study took place in Pima County, Arizona during the COVID-19 pandemic’s onset with recruitment done between 22 Aug and 05 December, 2020, prior to vaccine rollout in the United States. We conducted 10 focus group discussions (FGDs) with a total of 56 participants. Approximately 27 additional participants who had been recruited for the study failed to show up for their scheduled FGD for unknown reasons. We used purposive sampling methods for our study to ensure representation across race/ethnicities (White, Latinx and Black), Language (English and Spanish), and gender (Male and Female) from the networks of the study team. All participants provided informed consent before participating in any research activities. The Institutional Review Board at the University of Arizona reviewed and approved the study (Protocol ID: 2007796226).

We recruited participants to the study via social media, or email using ethical review board-approved recruitment scripts and digitally shared flyers. Participants often belonged to the same or similar social groups, such as an African American wellness group, or a group of college students familiar with a member of the research team, or a Hispanic activist group. Study team members would contact potential participants to invite them to participate in the study. Participants were included if they were above 18 years of age and were living in Pima County at the time of the study. Upon recruitment, participants were introduced to the research study and its purpose, fore-shadowing informed consent.

### Interview guide development

The semi-structured focus group discussion guide and prompts were developed iteratively by the team by using established qualitative methods [19]. The team conducted a pilot FGD internally before using the guide with participants; no repeat interviews were conducted.

### Interview setting

FGDs were held on an online virtual Zoom platform and lasted between 60–90 minutes. All participants were initially let into the Zoom room individually, while others were in the waiting room, to explain that they could change their name displayed on the Zoom call and hide their faces if they desired anonymity on the call. All participants completed a verbal informed consent process prior to the FGD. All sessions were audio and video recorded. A notetaker from the research team attended each session to take notes and provide support for the facilitator during each focus group.

### Data management

Debriefing sessions were held at the end of each FGD to discuss thematic saturation and notes for improvement in future sessions. Transcripts were not returned to participants for comment or correction. The automatic transcription feature of Zoom was used to generate initial transcripts for those held in English and then reviewed while listening to the audio recording by a research team member. Students who were studying Spanish translation were hired to transcribe and translate FGD sessions held in Spanish. For further details about the participants and the recruitment process and the analysis of data collected about seasonal influenza-related outcomes, refer to the article by Mantina et al [20].

### Reflexivity statement

The interviewers were a male Hispanic medical care provider practicing at the Pima County Health Department (CPV), a male Black PhD student (FM), a female professor of Indian origin, who was also the PI of the project with a PhD and medical degree (PM), and a female mixed race MPH student who was acting as study coordinator (MBN). All had some experience in qualitative research and received some additional training from the study team to ensure consistency. BK consulted on qualitative methods, interview design, and analysis. PM and CPV were the members of the team who conceptualized the project and determined that it was important to conduct this research to explore the evolving issues of a possible COVID-19 vaccine and possible factors that could be associated with future vaccine acceptance to inform a promotion strategy for the Pima County Health Department.

The qualitative analysis team included the lead MPH student (KE), two PhD students (MBN, LR), and a professor (PH). One of the PhD students (MBN) had been an interviewer, had previous experience in qualitative research, and served as the research project coordinator. One of the PhD students (LR) had limited experience in qualitative research and was studying Applied Social and Cultural Psychology. The professor of psychology (PH) who helped with the data analysis is a PhD holder and experienced researcher in social psychology and minority mental and public health research. All analysts identified as female, two as black (LR, PH), one as mixed race (MBN), and one as white (KE). Researchers who provided feedback and guidance on the qualitative research included one white male MPH student with more than three years of public health research experience (CL), a black female PhD student with at least 3 years of experience in qualitative research (NM), and a white female researcher with decades of experience in community-based qualitative research (BK).

### Theoretical framework

The National Institute on Minority Health and Health Disparities (NIMHD) framework accounts for social factors such as education and income [21]. In this study, we utilized this framework since it focused on Black, White, and Latinx communities. The NIMHD research framework is broadly used in the literature to explain the root causes of health disparities and social determinants of health in CoCs [21]. Building on the ideas of the socio-ecological model’s levels and concepts from a similar health disparities research framework from the National Institute of Aging (NIA), the NIMHD research framework is a “tool for conceptualizing and depicting the wide array of determinants that promote or worsen minority health or cause, sustain, or reduce health disparities” [21]. The framework was intended to help guide health disparities research by demonstrating different factors at different levels of society that may contribute to the risk and resilience factors related to health equity and inequities. The framework has two axes, with domains of health (biological, behavioral, physical and built environment, socio cultural environment, and healthcare system) depicted on the x-axis and levels of influence on health (individual, interpersonal, community, and societal) mapped onto the y-axis. This visual representation of the NIMHD framework also serves as a guide for research on health disparities, though it has yet to be used widely. It will be referred to as “the framework” henceforth. There is a more detailed description of the framework in **Appendix 1**.

### Data analysis

The qualitative analysis team began by familiarizing themselves with the FGD transcript data. A framework content analysis was using primarily a deductive approach when identifying themes, mapping them onto the framework as predetermined codes, which were applied in Dedoose. The team created a codebook of the Domains of Influence with definitions for each domain, including biological, social, psychological, etc. They collaboratively created matrices with definitions and examples to easily determine which type of data would be coded under each Domain of Influence. The Levels of Influence were included within each definition of the Domain codes. Emerging themes which may not have easily fit clearly under one Domain were additionally identified inductively and included as their own separate codes. When developing the codebook, the team discussed the overlap in the framework and determined through consensus which information was included within each code.

The transcripts were each coded by mapping the content from each transcript from each group onto the framework matrix by one person on the team. Subsequently, a second coder from the team reviewed the matrices for each group to verify for accuracy, taking notes and making tracked changes to the codes as necessary, reviewing the transcript as they reviewed the coded data. The team held regular discussions to discuss the codes that each team member applied to the transcript and resolved conflicts as a group through iterative discussion and consensus. During these meetings, suggested changes to the way the data were organized or “coded” onto the NIMHD framework matrices were discussed. We did not calculate inter coder reliability because conflicts were consistently resolved as a group and the dataset was smaller [22,23]. In order to ensure sufficient rigor, many discussions were held to ensure data were coded accurately, iteratively until all team members were satisfied. Each team member additionally took notes on the transcripts to further identify meanings, patterns, and comparisons among the data. The last step of the analysis involved in-depth discussion of each code applied in the transcript, assessing existing patterns and determining relevance to sociocultural factors, personal experience, medical history, and other relevant factors.

To display the examples of the themes that emerged, tables were created for each of the focus groups. While analyzing the data and developing themes, one additional code was added inductively to capture politics. The team created matrices and wrote summaries of findings for each racial/ethnic category of focus group and used these to create the results presented in this manuscript. See **Supplemental File 1** for the initial summaries made from each racial/ethnic category of focus groups. The codebook created based on the framework and its domains and corresponding definitions are included in **Appendix 1.** The Consolidated criteria for reporting qualitative research (COREQ) was used to inform how information was reported in this manuscript.

## Results

Of the 56 participants who took part in the ten focus groups, 34% were female. While 27% self-reported being Hispanic or Latinx, 25% were White, 9% were Black, and 4% were Asian, noting that some participants may have fallen under multiple categories. The preferred language was most frequently reported to be English (24, 43%) followed by Spanish (11, 20%). Demographic information was not reported for almost half of the participants (21, 38%). Although participants were grouped into focus groups based on their social groups, since they did not disclose their demographic information consistently, we will not report it at an individual level. It is important to note that some groups had small representation, limiting the interpretation of subgroup comparisons. The themes identified and mapped onto the NIMHD framework are reported in Table 1.

**Table 1:** NIMHD framework-aligned themes on vaccine perceptions and calculated risks by Racial/Ethnic group.

Domain	Black focus groups	Hispanic/Latino focus groups	White focus groups
**Biological** **Domain**	Family vulnerabilities (e.g., heart conditions)	Fear of infecting elderly parents	Individual immunity, calculated risk
**Behavioral Domain**	Concerns about long-term effects, protecting others	Self-isolation and strict precautions	Social responsibility, protecting others
**Physical/Built Environment**	Environmental hygiene (e.g., mask, gloves, cleaning car)	Room isolation, household distancing	Institutional concerns (school outbreaks)
**Sociocultural Environment**	Historical mistrust (Tuskegee)	Community-based trust and privacy	Trust in close circles, confusion over messaging
**Healthcare** **System**	Distrust due to testing bias & lack of representation	Conflicting medical advice & uncertainty	Concerns about rushed trials, desire for clear information
**Politics (Inductive Theme)**	Mistrust of CDC, trust in local health officials, distrust of political media	Belief that COVID is politically motivated	Distrust of political media, concern about rushed rollout

### Biological domain

Participants across racial groups highlighted biological factors affecting their vaccine-related decisions. Biological vulnerabilities, such as chronic conditions, significantly shaped attitudes and behaviors towards vaccination. For instance, a White male participant was concerned about the possibility of being infected while in public areas,

*“I think my risks of getting it are high. And I think I take precaution, and I am pretty proactive on taking care of myself. But even with me taking those steps and working, for I have the privilege of working from home, I think I still-- anytime you're in public, I think you're putting yourself at risk. So, I think there's a high probability of either encountering someone who has COVID-19 or is either connected. So, I think- my framework that I use is anytime you're out in public or outside you're at risk of getting it.” (p25)*

Black participants expressed awareness of familial vulnerabilities, illustrating heightened caution due to chronic illnesses among family members:

*“So, I just try to protect my grandchildren and my daughter, who has a bad heart. I tried to pick those, you know, things like that.” (p29)*

A Hispanic participant similarly emphasized maintaining health and protecting loved ones:

*“Immediately have hand sanitizer, or wash [my daughter’s] hands, and don’t touch anything, don’t touch your face […] You don’t pay attention to what you are going to do because you’re watching them. Because there is fear-- my fear is that she infects my parents because they are vulnerable.” (p36)*

Participants collectively recognized that individual health directly influenced decisions to vaccinate for broader community protection, with a Black male participant noting communal responsibility said:

*“I feel like it’s just my responsibility to the rest of the community” (p53).*

Black and Hispanic participants prominently emphasized familial and community biological vulnerabilities, contrasting with White participants, who focused more on individual health and robust immunity.

### Behavioral domain

Behavioral factors influencing preventive practices and vaccine hesitancy were articulated across groups. Hispanic participants frequently described cautious preventive measures at home and working on proactive strategies*.* One Hispanic participant added,

*“What I would do is isolate myself, you understand me, from my family as well to not get other people sick and that’s what we are doing right now” (p50).*

A Black participant expressed significant concerns regarding vaccine uncertainties, stating,

*“Long term health effects…they can't tell us what it does to us five years in the future, until it starts popping up five years in the future. So that's the biggest worry” (p53).*

White participants framed their decisions around familial responsibilities, stating,

“*Yeah, protect other people...my motivator is to protect them…it's my social responsibility not to be a vector, if possible” (p9)*.

Across groups, community responsibilities emerged as a common theme. Societal mandates played a significant role in shaping behaviors, particularly in cases where vaccination was required for immigration or employment purposes. Behavioral responses differed across racial and ethnic groups in our study: Black and Hispanic participants tended to express more cautious approaches influenced by uncertainties around the vaccine, while White participants more frequently emphasized responsibility to protect others in their social and family circles:

*“But that's like how I think about it like I don't want to get my parents sick. You know my grandmas are both 87 they would die” (p18).*

### Physical/Built environment

Physical and built environmental contexts influenced health behaviors among participants. A Black participant particularly emphasized rigorous environmental hygiene practices as critical preventive measures:

*“I don't go out to the malls and family gatherings and stuff where there's a lot of people, so therefore I go out, mainly when there's an essential reason and I try to do all those things. So, to distance myself, I use a mask and gloves if necessary. Then I pull them off and dispose of those gloves there at the store or where I am.I don't want any germs in my car. And when I pick up my groceries, I call them and pick them up from the store. I have to put it in place into my trunk because again I want nothing near me.” (p32)*

Similarly, a Hispanic male participant detailed significant home isolation strategies to prevent viral transmission within families, describing actions such as

*“She [my daughter] calls us from her work that she did not want to have direct contact with us, because a person came out positive, then what we did was that we started fixing the room and leaving the house to go live with another daughter in the same place.” (p55)*

A few White male participants briefly mentioned concerns about professional environments like schools, acknowledging risks within institutional settings, such as,

*" New cases in Tucson are being driven by the university student population, particularly the large student housing complexes. And so, I have concern for, for my colleagues who are teaching and interacting with those students.” (p24)*.

This domain revealed focused personal and familial environmental adjustments predominantly among Black and Hispanic participants. Black and Hispanic participants provided more detailed descriptions of proactive ecological strategies than White participants, who mainly acknowledged broader institutional environmental risks. Participants were more concerned about bringing the virus into their physical/built environments.

### Sociocultural environment

Sociocultural factors profoundly influenced participant perspectives, including historical mistrust, cultural norms, and social networks. Black participants notably referenced historical injustices, exemplified by the Tuskegee Syphilis Study. One participant emotionally stated,

*“Getting ready to get a little deep for a moment. Oh, the Tuskegee Syphilis Study done on African American. I want to say with all my goodness, maybe the 20s, 30s, 40s. I don't know the exact date, but that sticks in my mind, of giving People of color placebo … Oh, it makes me cry sometimes because it's so sad… I am hesitant to go to the doctors. There’s been [a] long line and a long history of African American people, whether we are older or younger, or female or male not being treated right in the health care system.” (p43)*

Some Hispanic participants placed significant trust in local communities, describing cultural privacy and preventive practices. As one participant said,

*“I think that in the Hispanic environment, or so to speak, there is a little more privacy in terms of people taking into account... Well, I am talking about the circle closest to me, and they take into account that we must follow the rules precisely so as not to become infected. For example, there are not so many parties, I don't know if they go to so many bars and all that.” (p55).*

A White male participant emphasized interpersonal trust within immediate social groups as pivotal:

*“I think … from my perspective, I think we've kept our circle pretty tight. And I think we've kind of been- especially during these times- we've kept it... Well, I think, trust, trust, trust in each other. And I think we've been hanging out a lot with like-minded people that kind of see this in the same light. So, trust really has played a big part in who we spend time with, who we hang out [with]. So, I think that has been a huge part in our, in our circle.” (p25)*

Societal influences also emerged clearly, with participants across groups expressing deep frustration over politicized and inconsistent health information: *“[T]hey confuse people more like that, and they scare them more because they are the experts;* *they should be constant” (p37).* Some Black participants highlighted systemic discrimination and historical mistrust. Hispanic participants often demonstrated strong community-centered trust, and White participants often focused on confusion due to societal misinformation.

These findings illustrate how vaccine decision-making was shaped not only by individual beliefs but also by interpersonal trust, community norms, and broader societal narratives. Within the NIMHD framework, sociocultural influences operated across levels: family and close social networks shaped perceived responsibility, community histories shaped mistrust, and politicized national discourse shaped confidence in public health institutions.

### Health care system

Participants expressed widespread skepticism and mistrust regarding healthcare systems, highlighting communication, accessibility, representation, and care quality concerns. Some Hispanic participants notably voiced confusion due to inconsistent messaging from healthcare authorities:

*“The ones who did the exam said she should stay seven more days after the test. The pediatrician tells me that she should stay ten more days. Who is right? They told me, ‘Well, in reality we don’t know.’ So, if you are trusting someone and they tell you ‘well we don’t know because it’s new.’ Who are you going to trust?” (p36)*

Some Black participants articulated specific apprehensions regarding different cultural representations in vaccine trials*.* A participant expressed broader mistrust:

*“Medications are done on white people and maybe white men mostly. Yeah, just save it. And so, we are in the minority when they are testing out these new medications, and then they are being used on you. Used in a negative way. A lot of work is done in Caribbean and African countries, and people are not well informed.” (p45)*

A White participant emphasized concerns about rapid vaccine development:

*“One of my concerns is because it has a profit motive, but also because it's a race right now. Not just cutting corners, but just efficacy may not be measured as stringent as it should be. And so, the first-generation vaccines may or may not be effective, but they also probably [have] been run through as much rigorous testing in order to have a time frame and that worries me.” (p8)*

Additionally, another White participant highlighted the need to understand vaccine implications clearly:

*“I'd want to know the effects of it. Is it you know, something, you get the vaccine, but you still have, you know, some odd percentage or chance of being exposed to it and getting it? It may just be mild or? I just want to understand If I did get the vaccine, what does that mean as far as risk of exposure, and if I got sick, what that means.” (p20)*

While mistrust towards healthcare systems was common across all groups, Black participants particularly emphasized representation issues and systemic barriers, Hispanic participants focused on confusion from inconsistent messaging, and White participants emphasized trust in interpersonal healthcare relationships.

#### Inductive theme: Politics.

In our analysis, we identified a significant theme not captured in the NIMHD Research Framework: the role of the political environment in influencing COVID-19 vaccine hesitancy. Participants across racial and ethnic groups expressed concerns about political interference potentially compromising the integrity of vaccine development. Some of the Hispanic/Latino participants felt that COVID-19 was fake, and being used by the government to cover something up and expressed a fear of what was being put into the vaccine. Participants from this group tended to not trust President Trump and reported that if the vaccine were to come out before the election, they would not trust the COVID-19 vaccine. Many participants also felt that scientists were disagreeing with each other, and that countries were competing instead of trying to develop a vaccine that would collectively help humanity. Similarly, a Hispanic female voiced frustration over the politicization of vaccine efforts, commenting,

*“I see more political taint in the countries, more than the desire… for curing this sickness” (p4).*

Some of the Black participants felt that those in power were able to influence what is being said in the media. Participants in this group mentioned that the COVID-19 vaccine was being too politicized. Distrust in federal health institutions was common. However, many did trust their local government officials. A Black mixed gender group participant noted,

*“I would not trust the CDC, the NIH, or any public health [officials] that he has control over… There's just too much politics and political pressure. But at the local level, like Dr. Garcia…I trust him…”* (p30).

Some of the White participants felt that more people would be willing to receive the COVID-19 vaccine if information was given via a non-political platform. They note that CNN, Fox News and MSNBC are very political. In line with the Hispanic/Latino and Black participants, White participants also felt that the vaccine was being rushed, and that President Trump was using the vaccine to grow his popularity.

*“I think it is completely politicized and that would be the only reason that it would be available is because Trump has gone through all the chains of commands to make that happen … in order to grow his base. I'm completely pro vaccine, but I really don't think I could get behind that.”* (p18)

For some, political views extended to personal beliefs, as shared by a White female participant:

*“My father… bought into the idea that COVID was a hoax… mainly because he didn’t know anyone who had gotten it”* (p10).

This theme highlights the importance of recognizing how political dynamics can shape public trust in health initiatives.

While all groups expressed concern about rushed vaccine development, the basis of concern differed. Black participants more often linked vaccine concerns to historical mistreatment and underrepresentation in clinical trials; Hispanic participants emphasized uncertainty, family protection, and inconsistent medical guidance; and White participants more often emphasized political interference, individual risk calculation, and the need for clear evidence. These differences suggest that similar expressions of hesitancy may arise from distinct social and structural contexts.

Across domains, vaccine intention was shaped by the interaction of perceived biological vulnerability, behavioral risk management, sociocultural trust, healthcare system mistrust, and political context. Participants did not describe vaccine decisions as isolated individual choices; rather, they framed decisions as negotiated through family responsibility, prior experiences with healthcare institutions, perceived credibility of messengers, and uncertainty about the speed and transparency of vaccine development. These findings suggest that vaccine hesitancy in this sample was best understood as a multilevel process rather than a single attitude or knowledge deficit.

## Discussion

By applying the National Institute on Minority Health and Health Disparities Research Framework in this study, we were able to highlight many factors that influenced adults in Pima County, Arizona to choose to vaccinate or not to vaccinate against COVID-19. There were many common themes among all FGDs, such as participants choosing to take the flu vaccine due to COVID-19 when they might otherwise have not received the flu vaccine. Another common theme shared across the FGDs was wanting to protect others, especially those close to them. A fear that the COVID-19 vaccine was being developed too fast was also mentioned across all FGDs.

Health literacy and misinformation were major concerns mentioned across groups, as participants voiced doubts about the accuracy of vaccine information and were skeptical about the vaccine’s effectiveness and safety due to conflicting messages. Concern about safety and side effects has also been mentioned in the literature [24]. Historical and sociopolitical mistrust were also common themes, especially concerning the government’s handling of the pandemic. Mistrust in healthcare systems and public health authorities also surfaced as a significant theme. Washington suggests that many in the Black community and possibly other CoC have a fear of the “white coat” or what is called iatrophobia [25]. Iatrophobia is associated with historical events that create what Grier and Cobbs long ago described as a type of cultural paranoia [26]. Vazquez et al. found that historical, cultural, and social trauma induce fear and mistrust in public health and medical institutions, influencing COVID-19 testing and vaccination decisions among Latinx, Indigenous Americans, and Black Americans [27]. Lastly, attitudes toward behavioral protective practices, such as masking and social distancing, also varied significantly across groups with Hispanic participants, especially women, appearing to be more inclined to view vaccination as part of a comprehensive approach necessary for their family’s safety.

While there were some similarities across all the FGDs, we found a few decisions surrounding vaccination that were specific to certain groups. It appeared that White participants noted the importance of effective messaging. They also appeared more likely to discuss nurturing one’s own immunity. In contrast, Black and Hispanics appeared more concerned about protecting friends and family. Black participants at times demonstrated a concern about clinical trials, in particular mentioning worries about if they were ethical and inclusive. They also were the only group to mention the Tuskegee Project as their reason to not trust the medical community, similar to other studies from the literature [24,28]. A qualitative study by Casselman-Hontalas et al found several participants referring to the Tuskegee syphilis study and felt that COVID-19 was being experimented on Blacks [29]. This demonstrates a similar need for better communication from researchers to the public regarding how these trials are being conducted. This emphasizes the importance of communicating everything from how a vaccine works to the extensive research and clinical trials involved in making a new vaccine.

As was mentioned in our study by Black participants, faith and religion appeared important to them in this context. One way to build trust in the Black community and increase vaccination rates is to involve those they trust such as faith leaders [30]. Participants in our study recommended implementing a faith-based approach, and the first step was to listen and get to know the community [30]. Many Black communities have been shown to trust their pastors in the community; therefore, these pastors may be effective messengers for vaccine-related information [30].

Our findings align with established vaccine hesitancy frameworks, including the “3Cs” model of confidence, complacency, and convenience and the expanded “5Cs” model, which includes confidence, complacency, constraints, calculation, and collective responsibility [31,32]. Participants’ concerns about rushed vaccine development, inconsistent messaging, and political interference reflect challenges related to confidence. Their questions about personal risk, immunity, and vaccine safety reflect calculation, while repeated references to protecting family members and community members reflect collective responsibility. However, our findings also suggest that these constructs are experienced through racialized and sociopolitical contexts, particularly among participants who linked vaccine concerns to historical mistreatment, underrepresentation in clinical trials, and mistrust of federal institutions.

Importantly, participants’ concerns should not be interpreted solely as misinformation or lack of health literacy. Rather, many concerns reflected reasonable responses to rapidly changing guidance, limited transparency about vaccine development, prior experiences of discrimination, and broader political mistrust. This distinction is important because interventions focused only on correcting misinformation may fail if they do not also address trust, representation, and institutional accountability.

Taken together, these findings suggest that vaccine communication strategies should be tailored not only by demographic group but also by the underlying source of concern. Messages addressing safety and trial transparency may be particularly important when concerns are rooted in mistrust of research. Language-concordant, family-centered communication may be especially useful when concerns involve protecting elders and navigating conflicting guidance. Nonpartisan, locally grounded messaging may be critical when hesitancy is shaped by political distrust. Across groups, trusted messengers, transparency about uncertainty, and acknowledgment of historical and contemporary inequities may be more effective than information-only approaches.

A strength of this study is the use of a nationally recognized framework on health disparities to determine factors affecting vaccine attitudes among a purposively sampled group of participants. Additionally, this is the first article to use the NIMHD framework in the field of vaccine hesitancy to date to our knowledge. This sample provided diverse perspectives from different communities within Pima County. The information is very locally relevant, making the findings internally valid, especially during this unique period during the COVID-19 pandemic. The findings could help to inform future health promotion campaigns for novel vaccines, especially during a pandemic. Additionally, it is important to emphasize the value of this study applying the NIMHD Minority Health and Health Disparities Research Framework to pre-rollout vaccine confidence perceptions in a diverse county-level context, showing how concerns about trust, health literacy, historical marginalization, access, and information environments operate across multiple levels of influence.

Nevertheless, we recognize that the study has its limitations. It is important to note that the findings are from data collected prior to vaccine rollout in the United States, and therefore reflect anticipated perceptions, concerns, and stated intentions under uncertainty rather than actual vaccine uptake behavior. The data were not collected with the NIMHD research framework in mind, which may mean that some of the information related to the framework may have been missing not due to the participants not mentioning it, but rather due to the data collection tool and facilitator not being oriented towards the framework and its domains. Additionally, there was some possibility of misinterpretation of the data, since the analysts were mostly not involved with the data collection, member checking was not performed due to the delay of the analysis post data collection, and many were not from Pima County as well. We acknowledge that formal back-translation was not conducted for Spanish focus groups, which may have affected nuance in interpretation. Zoom and technological issues additionally made it challenging for some participants to actively and comfortably engage in discussion which may have made it more challenging to have free and open dialogue or share accurate and complete information. The format may also have introduced digital divide bias, which could have caused a selection bias. This discomfort with the new technology, changing the format of the discussions from a focus group discussion to more of a group interview format, unintentionally, and also additionally making it more challenging to collect socio demographic data leading to some gaps in the demographic data collected. We also had a limited sample size, so while we made comparison between racial ethnic groups and participants, these participants do not represent everyone with the same race or ethnicity and so it is important to note that findings may not be generalizable. No member checking was conducted in this study which limits its validity, but this was not feasible since this was a secondary analysis conducted on the data years after the data were collected so the participants themselves would be removed from their thoughts from this specific time in their lives and therefore it would not have made sense to conduct. One final limitation of this study is that the use of a disparities framework may have made the analysis biased towards trying to find disparities rather than recognizing similarities too.

Scientists at the National Institute on Minority Health and Health Disparities are devoted to addressing and conducting research on health disparities and inequities. [33]. To help bridge the gap between health disparities and to improve vaccination uptake among minority populations, it will be important to have access to data with accurate reporting on vaccination status. Gaps in vaccine uptake will persist until we work towards addressing the health disparities and the drivers behind them. Future studies in Pima County may benefit from a follow-up study to determine how attitudes may have changed regarding these vaccines post-pandemic. It would additionally be important to conduct similar studies on other important emerging vaccines such as the human papillomavirus (HPV), human immunodeficiency virus (HIV) and malaria vaccines to determine how vaccine attitudes vary depending on the type of vaccine.

## Conclusion

This study adds to the health disparities literature, demonstrating how the COVID-19 pandemic exacerbated health disparities in this country. It is vital that policies should address socioeconomic factors to help reduce these disparities [18]. A paper by Strully et al mentioned that some states had put into place strategies to increase equity while distributing vaccines [28]. However, they note that for this to work, stakeholders need to engage the community and gain their trust [28]. In summary, the driving forces behind vaccine hesitancy may be better understood when categorized into behavioral, biological, sociocultural, and healthcare, and political domains. By applying the NIMHD research framework lens to vaccine hesitancy, it is apparent that there are many opportunities for policymakers and other stakeholders to implement interventions that address different racial/ethnic issues among vaccine uptakes. We encourage future studies to explore the intersectionality of different sociodemographic variables and their impacts on health disparities regarding vaccine hesitancy.
