# Supplementary material for: COVID-19 vaccine confidence among adults of pima county using the NIMHD minority health and health disparities research framework: A qualitative analysis
Source: PLoS One. 2026 Jul 22;21(7):e0353345. doi: 10.1371/journal.pone.0353345 (PMC13390816; doi:10.1371/journal.pone.0353345)
Supplement: Attachment — Submitted filename: Review File PONE-D-25-63283.docx [file pone.0353345.s001.docx]

| Line No. | Technical Issue | Description |
| --- | --- | --- |
| 47–49 | Missing Coder Reliability | Mistake: You mention "four independent coders" but fail to report an Inter-rater Reliability (IRR) metric (e.g., Cohen’s Kappa or Percent Agreement).  Technical Impact: Without this, the reader cannot verify the consistency of the thematic analysis. This is a common "Major Revision" or "Rejection" point in qualitative research. |
| 48 | Transcription/Translation Gap | Mistake: The line is physically broken, but technically it misses the Back-Translation verification step. If FGDs were conducted in Spanish, standard protocol requires a description of how translation accuracy was verified (e.g., back-translation by a third party). |
| 50 | Ethics Sequence | Mistake: The mention of "Informed Consent" is tucked into the analysis description.  Technical Impact: Ethical approval and consent must be the first item in the Methods section, usually including the IRB Protocol Number, which is missing here. |
| 472 | Validation Failure | Mistake: (From the back snippet/limitations) "Member checking was not performed."  Technical Impact: In qualitative studies, member checking (returning transcripts to participants) is a primary tool for Credibility (Internal Validity). Its absence significantly weakens the study's rigor. |

| 53–54 | Denominator Inconsistency | Mistake: You report percentages (27%, 25%, 9%, 4%) for a sample size of N=56.  Technical Impact: These percentages result in non-whole numbers of people (e.g., 27% of 56 is 15.12). In clinical/social science reporting, if the denominator is under 100, you must report the raw number (n) alongside the percentage, or the math appears fabricated. |
| --- | --- | --- |
| 54 | Under-representation | Mistake: Reporting 4% for a racial group in a sample of 56 means only 2 people represented that entire category.  Technical Impact: This is statistically insufficient to draw thematic conclusions for that specific demographic, yet the abstract implies generalizable findings across these groups. |
| 56 | Missing NIMHD Mapping | Mistake: You claim to use the NIMHD Framework, but the results snippet (Page 56) shows codes like "(Insurance Coverage)" and "(Health Literacy)" without mapping them to the specific Levels of Influence (Individual, Interpersonal, Community, Societal). |

| Back Snippet | Selection Bias | Mistake: The focus groups were conducted virtually (Line 46).  Technical Impact: This introduces a significant "Digital Divide" bias. Participants without stable internet or high health literacy (as mentioned in line p21) were likely excluded, yet the study aims to examine "health disparities." This contradiction must be addressed in the methodology as a selection bias. |
| --- | --- | --- |
| Back Snippet | Thematic Overlap | Mistake: The codes "(Patient-Clinician Relationship)" and "(Health Literacy)" are applied to the same participant (p23).  Technical Impact: This suggests a lack of Mutual Exclusivity in your codebook. If codes overlap too much, the thematic analysis lacks "Discriminant Validity." |
| 940 | Metadata Leak | Mistake: The presence of a stray number like "940" suggests the manuscript was generated from a database or old versioning software  Technical Impact: This signals to the editor that the "Clean Copy" protocol was not followed, often leading to a desk rejection for lack of professionalism. |
