# Supplementary material for: COVID-19 vaccine confidence among adults of pima county using the NIMHD minority health and health disparities research framework: A qualitative analysis
Source: PLoS One. 2026 Jul 22;21(7):e0353345. doi: 10.1371/journal.pone.0353345 (PMC13390816; doi:10.1371/journal.pone.0353345)
Supplement: Attachment — Submitted filename: Cover Letter_NIMHD Paper Responses.docx [file pone.0353345.s004.docx]

April 23, 2026

**Title:** *COVID-19 Vaccine Confidence Among Adults of Pima County using the NIMHD Minority Health and Health Disparities Research Framework: A Qualitative Analysis*

Dear Editor and Reviewers,

We sincerely thank the Academic Editor and Reviewers for their thoughtful and constructive feedback on our manuscript. We appreciate the time and care taken to evaluate this work. We have carefully revised the manuscript in response to all comments and believe the paper has been substantially strengthened as a result.

Major revisions include: clarifying recruitment and eligibility procedures; improving demographic reporting and table presentation; revising the manuscript to clearly reflect that data were collected prior to COVID-19 vaccine rollout and therefore represent anticipated perceptions and intentions rather than actual vaccine uptake; expanding the description of qualitative rigor and coding procedures; strengthening the mapping of findings to the NIMHD Minority Health and Health Disparities Research Framework; tempering conclusions regarding subgroup differences; improving discussion of the study’s contribution to existing literature; revising tables for readability; and moving supplementary tables to Supporting Information files.

Below we provide a detailed point-by-point response to each comment. All changes are reflected in the revised manuscript and tracked-changes version. Page and line numbers refer to the revised manuscript.

We are grateful to the Editor and Reviewers for their careful reading and constructive feedback. Their comments helped us improve the manuscript’s clarity, rigor, and contribution. We hope that the revisions have addressed all concerns and that the revised manuscript will now be suitable for publication in PLOS ONE.

Sincerely,

Maiya Block Ngaybe PhD, MPH

On behalf of all authors

**Responses to Journal Requirements / Editor Comments**

| **Comment** | **Response** |
| --- | --- |
| 1. Please ensure that your manuscript meets PLOS ONE's style requirements, including those for file naming. | Thank you. We carefully reviewed the manuscript and submission files for compliance with PLOS ONE style requirements, including formatting, file naming, tables, references, and supporting information labeling. We also reviewed the manuscript against the PLOS ONE formatting templates and revised the files accordingly. |
| 2. We note that the grant information you provided in the ‘Funding Information’ and ‘Financial Disclosure’ sections do not match. Please ensure correct grant numbers are provided. | Thank you for identifying this inconsistency. We have corrected and harmonized the funding information and financial disclosure statements across the submission system and manuscript files to ensure consistency, including the correct grant number for MBN’s support.  The revised funding statement now reads:  *“This study was conducted using PM’s start-up funds. The Pima County Health Department provided some funds to aid data collection. The content is solely the responsibility of the authors and does not necessarily represent the official views of the funders. MBN was supported by the National Institutes of Health, Fogarty International Center/ Global Health Emerging Scholars Fellowship (D43TW010540). There was no additional external funding received for this study. The content is solely the responsibility of the authors and does not necessarily represent the official views of the funders.”*  These revisions are reflected in the manuscript on page [24], lines [596-597]. |
| 3. Please state what role the funders took in the study. If the funders had no role, include the statement in the cover letter. | Thank you. We have included the requested funder role statement in the cover letter and revised disclosure materials.  The statement is:  *“The funders had no role in study design, data collection and analysis, decision to publish, or preparation of the manuscript.”* |
| 4. Please confirm whether the submission contains all raw data required to replicate the study and revise the Data Availability Statement accordingly. | Thank you for this important request. We revised the Data Availability Statement to more accurately reflect the nature of the qualitative data and the journal’s data-sharing expectations.:  *“Since our data contain potentially identifying or sensitive patient information, we have provided our ethical review board information in case someone would like to request permission to access our data and can make a reasonable case for why they would need access to the data. Contact:* vpr-irb@arizna.edu.*”* |
| 5. Please include a new copy of Table 1–7 in your manuscript; the current table is difficult to read. | Thank you. We reformatted and replaced Tables 1–7 to improve readability and consistency with PLOS ONE table guidelines. |
| 6. Supplementary tables are included in the manuscript file. Please remove them and upload them as Supporting Information, with legends listed in the manuscript after the references. | Thank you. We removed the supplementary tables from the main manuscript and uploaded them separately as Supporting Information files. We also added corresponding legends after the references section in the manuscript, as requested. |
| 7. If reviewers recommended citations, evaluate whether they are relevant and should be cited. | Thank you. We reviewed all recommended areas for additional citation and revised the discussion to better situate our findings in the existing literature where appropriate. We added relevant citations where they strengthened contextualization and interpretation. |
| 8. Please review the reference list for completeness and correctness, including any retracted articles. | Thank you. We carefully reviewed and revised the reference list for completeness, formatting consistency, and accuracy. We also checked the cited literature to ensure that no retracted references were included unintentionally. Any changes to the reference list are reflected in the revised manuscript. |
| **Responses to Reviewer #1** | |
| Comment 1: Clarify sampling details and temper conclusions about racial/ethnic differences. Incomplete demographic reporting and small subgroup representation make structured racial/ethnic comparisons too strong. | Thank you for this important observation. We agree and have revised the manuscript to present subgroup findings more cautiously. We now clarify that demographic information was incomplete for a portion of participants and that some groups had small representation, limiting the interpretation of subgroup comparisons. We revised the relevant sections in the Results and Discussion to avoid overstatement and to frame these findings as observed patterns within this sample and focus group context, rather than broader racial or ethnic group characteristics. See results and discussion sections. |
| Comment 2: Emphasize that findings reflect pre-rollout perceptions rather than actual vaccine behavior. | Thank you. We agree and revised the manuscript throughout to make clear that data were collected between August and December 2020, prior to vaccine rollout in the United States, and therefore reflect anticipated perceptions, concerns, and stated intentions under uncertainty rather than actual vaccine uptake behavior. We revised the Methods and discussion section to make this clear. |
| Comment 3: Provide more transparency regarding qualitative rigor, especially given no member checking. Clarify how credibility and consistency across coders were maintained. | Thank you. We expanded the Methods section to provide greater detail on the steps used to support qualitative rigor and analytic consistency. Specifically, we clarified coder training, the process for codebook development and refinement, how transcripts were independently reviewed, and how discrepancies were discussed and resolved through consensus among the research team. We also expanded the Limitations section to acknowledge that member checking was not performed and to explain this as a limitation while describing other steps taken to support credibility and dependability. |
| Comment 4: Avoid overgeneralizing group distinctions. These should be described as observed patterns within specific focus groups. | Thank you. We agree and have revised the manuscript to avoid overgeneralized statements about racial and ethnic groups. The language now reflects that these were patterns observed within this purposive sample and focus group setting, rather than definitive group-level characteristics. |
| Comment 5: Deepen the discussion to clarify contribution beyond existing literature. | Thank you for this helpful suggestion. We revised the Discussion to move beyond summary and more clearly articulate how this study contributes to the literature. |
| Comment 6: Improve demographic clarity and editorial consistency. | Thank you. We revised the demographic reporting to improve clarity, including updating the participant characteristics table to present counts and percentages more transparently and noting where data were missing or not disclosed. We also reviewed the manuscript for minor editorial and formatting inconsistencies, including reference formatting. |
| **Responses to Reviewer #2** | |
| Comment 1: Recruitment procedures and inclusion/exclusion criteria are insufficiently described. | Thank you. We expanded the Methods section to provide additional detail on recruitment procedures, including how participants were identified and invited, the recruitment setting and approach, and the inclusion criteria used for participation. |
| Comment 2: Demographic reporting is incomplete and percentages do not total 100%. | Thank you. We have clarified in the methods section that we do not have consistent demographic information and the totals are not 100% because some participants belonged to multiple groups. |
| Comment 3: The study does not discuss thematic saturation. | Thank you, we did, but called it “data saturation” so adjusted the wording accordingly. |
| Comment 4: Analytic procedures, including intercoder reliability and resolution of disagreements, are not fully described. | Thank you. We revised the Methods section to better describe the analytic process, including how coding consistency was supported, and how coding disagreements were resolved through iterative discussion and consensus. |
| Comment 5: Mapping of findings to the NIMHD framework domains is not clearly demonstrated. | Thank you. We strengthened the presentation of the NIMHD framework throughout the manuscript. Specifically, we revised the Results to more explicitly identify how themes align with NIMHD levels and domains, and we clarified this mapping in the text and tables/[figure, if added]. We also revised the Discussion to explain how the framework helped structure interpretation of multilevel determinants of vaccine confidence. |
| Comment 6: Data were collected before vaccine rollout, so findings reflect anticipated intentions rather than actual confidence or uptake. | Thank you. We agree and revised the manuscript throughout to clarify the temporal context of the data. We now consistently describe the findings as reflecting pre-rollout perceptions, anticipated vaccine intentions, and early confidence concerns, rather than actual uptake behavior. |
| Comment 7: Data availability is unclear; de-identified qualitative data or excerpts should be clarified. | Thank you. We revised the Data Availability Statement to clarify what materials are available and, where applicable, the reasons full transcripts are not publicly shared due to confidentiality and re-identification concerns. We also ensured that representative excerpts and supporting materials are included as appropriate in the Appendices and that the statement clearly explains the mechanism for accessing data if permissible. |
| Comment 8: Findings are largely descriptive. Greater analytic depth is needed, including explicit linkage of themes to the framework and illustrative participant quotations. | We agree with the reviewer that the original Results section was largely descriptive. To address this, we adjusted the results and added more interpretation. We also added a cross-domain synthesis paragraph to clarify how biological vulnerability, behavioral risk management, sociocultural trust, healthcare system mistrust, and political context interacted to shape vaccine intention. |
| Comment 9: Some interpretations overextend the data and should be tempered. | Thank you. We carefully reviewed the manuscript and revised interpretations that may have overstated the implications of the findings. We now use more cautious language in the Abstract, Results, Discussion, and Conclusion, particularly regarding subgroup comparisons and implications for behavior. |
| Comment 10: The discussion could better situate findings in existing vaccine hesitancy literature. | Thank you. We expanded the Discussion to better contextualize our findings within prior literature on vaccine confidence, mistrust. This helps clarify both what is consistent with prior studies and what is distinctive about our findings. |
| **Responses to Reviewer #3** | |
| Comment 1: Four independent coders are mentioned, but no inter-rater reliability metric is reported. | Thank you for raising this point. We revised the Methods section to more clearly describe our coding process and the procedures used to support analytic consistency. |
| Comment 2: If focus groups were conducted in Spanish, translation verification or back-translation should be described. | Thank you. We did not have funding for translation verification or back-translation due to the funding limitations for this research, which is why we do not report it. We further acknowledge in the Limitations that formal back-translation was not conducted, which may have affected nuance in interpretation. |
| Comment 3: Ethical approval and informed consent should appear earlier in the Methods, including IRB protocol number. | Thank you. We revised the Methods section so that ethical approval and informed consent are presented clearly and prominently at the beginning of the Methods. The IRB protocol number is provided. |
| Comment 4: Member checking was not performed, which weakens rigor. | Thank you. We agree that the absence of member checking should be clearly acknowledged. We retained this as a limitation and expanded the manuscript to describe alternative strategies used to support credibility, including multiple coders, iterative discussion, codebook refinement, and team consensus. We also clarify the practical reasons member checking was not feasible in this study. |
| Comment 5: Percentages are reported without raw numbers, and denominator inconsistency makes interpretation difficult. | Thank you. We have clarified in the methods section that we do not have consistent demographic information and the totals are not 100% because some participants belonged to multiple groups. |
| Comment 6: Very small subgroup representation limits the ability to draw thematic conclusions for those groups. | Thank you. We agree and have tempered the manuscript accordingly. We revised the Abstract, Results, and Discussion to avoid implying broad generalizability from very small subgroup representation. We now describe these as sample-specific observations and explicitly note this limitation. |
| Comment 7: The NIMHD framework is referenced, but the mapping to levels of influence is not explicit enough. | Thank you. We strengthened the framework application by explicitly explaining the mapping of themes to relevant NIMHD levels and domains clearer in the Methods. |
| Comment 8: Virtual focus groups may introduce digital divide bias, which should be addressed as a selection bias. | Thank you for this important observation. We added discussion of this issue to the Limitations section. |
| Comment 9: Thematic overlap between codes may indicate insufficient discriminant validity. | Thank you. We appreciate this point. However, we do not believe there is thematic overlap, since each theme has distinct differences based on the framework and therefore do not believe this is an issue. We discussed the definition of the overlap and came up with specific inclusion criteria during our iterative discussions to ensure validity. |
| Comment 10: Presence of a stray number such as “940” suggests manuscript cleanup issues. | Thank you for identifying this. We carefully reviewed the manuscript and removed stray metadata, formatting artifacts, and versioning remnants to ensure a clean revised submission. |
