# Supplementary material for: COVID-19 vaccine confidence among adults of pima county using the NIMHD minority health and health disparities research framework: A qualitative analysis
Source: PLoS One. 2026 Jul 22;21(7):e0353345. doi: 10.1371/journal.pone.0353345 (PMC13390816; doi:10.1371/journal.pone.0353345)
Supplement: Attachment — Submitted filename: PONE-D-25-63283_R1.docx [file pone.0353345.s003.docx]

**PLOS One**

**COVID-19 Vaccine Confidence Among Adults of Pima County using the NIMHD Minority Health and Health Disparities Research Framework: A Qualitative Analysis**

--Manuscript Draft--

| **Manuscript Number:** | PONE-D-25-63283R1 |
| --- | --- |
| **Article Type:** | Research Article |
| **Full Title:** | COVID-19 Vaccine Confidence Among Adults of Pima County using the NIMHD Minority Health and Health Disparities Research Framework: A Qualitative Analysis |
| **Short Title:** | COVID-19 Vaccine Confidence in Pima County Adults: A Qualitative Study Using the NIMHD Framework |
| **Corresponding Author:** | Purnima Madhivanan  The University of Arizona Mel and Enid Zuckerman College of Public Health Tucson, Arizona UNITED STATES OF AMERICA |
| **Keywords:** | Vaccination Refusal; COVID-19 vaccine; patient acceptance of health care; Trusted Sources of Information; Health Disparities |
| **Abstract:** | Introduction  Vaccinations have been established as one of the most effective strategies to prevent the spread of infectious diseases. However, vaccine hesitancy has been a growing issue that needs to be monitored and understood to ensure successful promotion.  Historic structural, social, and health disparities have been demonstrated to have significant impact on many health outcomes including vaccination uptake. This study applied the National Institute on Minority Health and Health Disparities Research Framework to examine factors influencing COVID-19 vaccination intention during the COVID-19 pandemic among adults in Pima County, Arizona.  Methods  In the Fall of 2020, 10 focus group discussions stratified by race/ethnicity, gender and language spoken (FGDs) were conducted virtually among 56 adult residents of Pima County, Arizona. After transcription and translation of FGDs conducted in Spanish, transcripts were organized by demographic characteristics and coded in Dedoose software by four independent coders. The NIMHD Framework’s Domains of Influence were used as a priori codes in the thematic analysis. All participants completed an informed consent process prior to participation.  Results  Of the 56 participants who took part in the ten focus groups, 27% self-reported being Hispanic or Latinx, 25% were White/non-Hispanic, 9% were Black, and 4% were Asian. The preferred language was most often reported as English. Unique challenges for each demographic group emerged from this analysis, such as language barriers among Hispanic participants, historical mistreatment concerns among Black participants, and conflicting political messages for White participants. Cultural norms, family influences, and attitudes toward autonomy versus community responsibility further shaped vaccine decisions. Effective communication about vaccine safety, clinical trials, and public health messaging were identified as critical to addressing mistrust and vaccine confidence across diverse groups.  Conclusion  Major themes in this study that predict vaccine intention include health literacy challenges, misinformation, mistrust in healthcare systems, and the desire to protect others. Understanding the behaviors and decisions behind vaccination acceptance and receipt is important for developing effective vaccine communication strategies.  Identifying health disparities driving vaccination hesitancy may help guide future strategies and policies to improve vaccination rates, especially among minority groups. |

| **Order of Authors:** | Kim Ellis, MSc, MPH |
| --- | --- |
|  | Pamela Hall, PhD |
|  | Latrisha Robinson |
|  | Sara Ruiz |
|  | Maiya G Block Ngaybe, MPH, PhD |
|  | Christopher Lessenich, MPH |
|  | Namoonga M. Mantina, MsPH, MBA, PhD |
|  | Carlos M Perez-Velez, MD |
|  | Beatrice Krauss, PhD |
|  | Purnima Madhivanan, MBBS, MPH, PhD |
| **Response to Reviewers:** | Dear Editor and Reviewers,  We sincerely thank the Academic Editor and Reviewers for their thoughtful and constructive feedback on our manuscript. We appreciate the time and care taken to evaluate this work. We have carefully revised the manuscript in response to all comments and believe the paper has been substantially strengthened as a result.  Major revisions include: clarifying recruitment and eligibility procedures; improving demographic reporting and table presentation; revising the manuscript to clearly reflect that data were collected prior to COVID-19 vaccine rollout and therefore represent anticipated perceptions and intentions rather than actual vaccine uptake; expanding the description of qualitative rigor and coding procedures; strengthening the mapping of findings to the NIMHD Minority Health and Health Disparities Research Framework; tempering conclusions regarding subgroup differences; improving discussion of the study’s contribution to existing literature; revising tables for readability; and moving supplementary tables to Supporting Information files.  Below we provide a detailed point-by-point response to each comment. All changes are reflected in the revised manuscript and tracked-changes version. Page and line numbers refer to the revised manuscript.  We are grateful to the Editor and Reviewers for their careful reading and constructive feedback. Their comments helped us improve the manuscript’s clarity, rigor, and contribution. We hope that the revisions have addressed all concerns and that the revised manuscript will now be suitable for publication in PLOS ONE.  Sincerely,  Maiya Block Ngaybe PhD, MPH On behalf of all authors  Responses to Journal Requirements / Editor Comments  CommentResponse   1. Please ensure that your manuscript meets PLOS ONE's style requirements, including those for file naming.Thank you. We carefully reviewed the manuscript and submission files for compliance with PLOS ONE style requirements, including formatting, file naming, tables, references, and supporting information labeling. We also reviewed the manuscript against the PLOS ONE formatting templates and revised the files accordingly. 2. We note that the grant information you provided in the ‘Funding Information’ and ‘Financial Disclosure’ sections do not match. Please ensure correct grant numbers are provided.Thank you for identifying this inconsistency. We have corrected and harmonized the funding information and financial disclosure statements across the |

|  | submission system and manuscript files to ensure consistency, including the correct grant number for MBN’s support.  The revised funding statement now reads:  “This study was conducted using PM’s start-up funds. The Pima County Health Department provided some funds to aid data collection. The content is solely the responsibility of the authors and does not necessarily represent the official views of the funders. MBN was supported by the National Institutes of Health, Fogarty International Center/ Global Health Emerging Scholars Fellowship (D43TW010540). There was no additional external funding received for this study. The content is solely the responsibility of the authors and does not necessarily represent the official views of the funders.”  These revisions are reflected in the manuscript on page [24], lines [596-597].   3. Please state what role the funders took in the study. If the funders had no role, include the statement in the cover letter.   Thank you. We have included the requested funder role statement in the cover letter and revised disclosure materials.  The statement is:  “The funders had no role in study design, data collection and analysis, decision to publish, or preparation of the manuscript.”   4. Please confirm whether the submission contains all raw data required to replicate the study and revise the Data Availability Statement accordingly.Thank you for this important request. We revised the Data Availability Statement to more accurately reflect the nature of the qualitative data and the journal’s data-sharing expectations.:   “Since our data contain potentially identifying or sensitive patient information, we have provided our ethical review board information in case someone would like to request permission to access our data and can make a reasonable case for why they would need access to the data. Contact: [vpr-irb@arizna.edu](mailto:vpr-irb@arizna.edu).”   5. Please include a new copy of Table 1–7 in your manuscript; the current table is difficult to read.   Thank you. We reformatted and replaced Tables 1–7 to improve readability and consistency with PLOS ONE table guidelines.   6. Supplementary tables are included in the manuscript file. Please remove them and upload them as Supporting Information, with legends listed in the manuscript after the references.   Thank you. We removed the supplementary tables from the main manuscript and uploaded them separately as Supporting Information files. We also added corresponding legends after the references section in the manuscript, as requested.   7. If reviewers recommended citations, evaluate whether they are relevant and should be cited.   Thank you. We reviewed all recommended areas for additional citation and revised the discussion to better situate our findings in the existing literature where appropriate. We added relevant citations where they strengthened contextualization and interpretation.   8. Please review the reference list for completeness and correctness, including any retracted articles.   Thank you. We carefully reviewed and revised the reference list for completeness, formatting consistency, and accuracy. We also checked the cited literature to ensure that no retracted references were included unintentionally. Any changes to the reference list are reflected in the revised manuscript.  Responses to Reviewer #1  Comment 1: Clarify sampling details and temper conclusions about racial/ethnic differences. Incomplete demographic reporting and small subgroup representation make structured racial/ethnic comparisons too strong.  Thank you for this important observation. We agree and have revised the manuscript to present subgroup findings more cautiously. We now clarify that demographic information was incomplete for a portion of participants and that some groups had small representation, limiting the interpretation of subgroup comparisons. We revised the relevant sections in the Results and Discussion to avoid overstatement and to frame these findings as observed patterns within this sample and focus group context, rather than broader racial or ethnic group characteristics. See results and discussion sections.  Comment 2: Emphasize that findings reflect pre-rollout perceptions rather than actual vaccine behavior. |
| --- | --- |

|  | Thank you. We agree and revised the manuscript throughout to make clear that data were collected between August and December 2020, prior to vaccine rollout in the United States, and therefore reflect anticipated perceptions, concerns, and stated intentions under uncertainty rather than actual vaccine uptake behavior. We revised the Methods and discussion section to make this clear.  Comment 3: Provide more transparency regarding qualitative rigor, especially given no member checking. Clarify how credibility and consistency across coders were maintained.  Thank you. We expanded the Methods section to provide greater detail on the steps used to support qualitative rigor and analytic consistency. Specifically, we clarified coder training, the process for codebook development and refinement, how transcripts were independently reviewed, and how discrepancies were discussed and resolved through consensus among the research team. We also expanded the Limitations section to acknowledge that member checking was not performed and to explain this as a limitation while describing other steps taken to support credibility and dependability.  Comment 4: Avoid overgeneralizing group distinctions. These should be described as observed patterns within specific focus groups.  Thank you. We agree and have revised the manuscript to avoid overgeneralized statements about racial and ethnic groups. The language now reflects that these were patterns observed within this purposive sample and focus group setting, rather than definitive group-level characteristics.  Comment 5: Deepen the discussion to clarify contribution beyond existing literature.Thank you for this helpful suggestion. We revised the Discussion to move beyond summary and more clearly articulate how this study contributes to the literature.  Comment 6: Improve demographic clarity and editorial consistency.  Thank you. We revised the demographic reporting to improve clarity, including updating the participant characteristics table to present counts and percentages more transparently and noting where data were missing or not disclosed. We also reviewed the manuscript for minor editorial and formatting inconsistencies, including reference formatting.  Responses to Reviewer #2  Comment 1: Recruitment procedures and inclusion/exclusion criteria are insufficiently described.Thank you. We expanded the Methods section to provide additional detail on recruitment procedures, including how participants were identified and invited, the recruitment setting and approach, and the inclusion criteria used for participation.  Comment 2: Demographic reporting is incomplete and percentages do not total 100%. Thank you. We have clarified in the methods section that we do not have consistent demographic information and the totals are not 100% because some participants belonged to multiple groups.  Comment 3: The study does not discuss thematic saturation.  Thank you, we did, but called it “data saturation” so adjusted the wording accordingly. Comment 4: Analytic procedures, including intercoder reliability and resolution of disagreements, are not fully described.  Thank you. We revised the Methods section to better describe the analytic process, including how coding consistency was supported, and how coding disagreements were resolved through iterative discussion and consensus.  Comment 5: Mapping of findings to the NIMHD framework domains is not clearly demonstrated.  Thank you. We strengthened the presentation of the NIMHD framework throughout the manuscript. Specifically, we revised the Results to more explicitly identify how themes align with NIMHD levels and domains, and we clarified this mapping in the text and tables/[figure, if added]. We also revised the Discussion to explain how the framework helped structure interpretation of multilevel determinants of vaccine confidence.  Comment 6: Data were collected before vaccine rollout, so findings reflect anticipated intentions rather than actual confidence or uptake.  Thank you. We agree and revised the manuscript throughout to clarify the temporal context of the data. We now consistently describe the findings as reflecting pre-rollout perceptions, anticipated vaccine intentions, and early confidence concerns, rather than actual uptake behavior.  Comment 7: Data availability is unclear; de-identified qualitative data or excerpts should be clarified. |
| --- | --- |

|  | Thank you. We revised the Data Availability Statement to clarify what materials are available and, where applicable, the reasons full transcripts are not publicly shared due to confidentiality and re-identification concerns. We also ensured that representative excerpts and supporting materials are included as appropriate in the Appendices and that the statement clearly explains the mechanism for accessing data if permissible.  Comment 8: Findings are largely descriptive. Greater analytic depth is needed, including explicit linkage of themes to the framework and illustrative participant quotations.  We agree with the reviewer that the original Results section was largely descriptive. To address this, we adjusted the results and added more interpretation. We also added a cross-domain synthesis paragraph to clarify how biological vulnerability, behavioral risk management, sociocultural trust, healthcare system mistrust, and political context interacted to shape vaccine intention.  Comment 9: Some interpretations overextend the data and should be tempered. Thank you. We carefully reviewed the manuscript and revised interpretations that may have overstated the implications of the findings. We now use more cautious language in the Abstract, Results, Discussion, and Conclusion, particularly regarding subgroup comparisons and implications for behavior.  Comment 10: The discussion could better situate findings in existing vaccine hesitancy literature.  Thank you. We expanded the Discussion to better contextualize our findings within prior literature on vaccine confidence, mistrust. This helps clarify both what is consistent with prior studies and what is distinctive about our findings.  Responses to Reviewer #3  Comment 1: Four independent coders are mentioned, but no inter-rater reliability metric is reported.Thank you for raising this point. We revised the Methods section to more clearly describe our coding process and the procedures used to support analytic consistency.  Comment 2: If focus groups were conducted in Spanish, translation verification or back-translation should be described.  Thank you. We did not have funding for translation verification or back-translation due to the funding limitations for this research, which is why we do not report it. We further acknowledge in the Limitations that formal back-translation was not conducted, which may have affected nuance in interpretation.  Comment 3: Ethical approval and informed consent should appear earlier in the Methods, including IRB protocol number.Thank you. We revised the Methods section so that ethical approval and informed consent are presented clearly and prominently at the beginning of the Methods. The IRB protocol number is provided.  Comment 4: Member checking was not performed, which weakens rigor.  Thank you. We agree that the absence of member checking should be clearly acknowledged. We retained this as a limitation and expanded the manuscript to describe alternative strategies used to support credibility, including multiple coders, iterative discussion, codebook refinement, and team consensus. We also clarify the practical reasons member checking was not feasible in this study.  Comment 5: Percentages are reported without raw numbers, and denominator inconsistency makes interpretation difficult.  Thank you. We have clarified in the methods section that we do not have consistent demographic information and the totals are not 100% because some participants belonged to multiple groups.  Comment 6: Very small subgroup representation limits the ability to draw thematic conclusions for those groups.  Thank you. We agree and have tempered the manuscript accordingly. We revised the Abstract, Results, and Discussion to avoid implying broad generalizability from very small subgroup representation. We now describe these as sample-specific observations and explicitly note this limitation.  Comment 7: The NIMHD framework is referenced, but the mapping to levels of influence is not explicit enough.  Thank you. We strengthened the framework application by explicitly explaining the mapping of themes to relevant NIMHD levels and domains clearer in the Methods. Comment 8: Virtual focus groups may introduce digital divide bias, which should be addressed as a selection bias.  Thank you for this important observation. We added discussion of this issue to the Limitations section. |
| --- | --- |

|  | Comment 9: Thematic overlap between codes may indicate insufficient discriminant validity.  Thank you. We appreciate this point. However, we do not believe there is thematic overlap, since each theme has distinct differences based on the framework and therefore do not believe this is an issue. We discussed the definition of the overlap and came up with specific inclusion criteria during our iterative discussions to ensure validity.  Comment 10: Presence of a stray number such as “940” suggests manuscript cleanup issues.  Thank you for identifying this. We carefully reviewed the manuscript and removed stray metadata, formatting artifacts, and versioning remnants to ensure a clean revised submission. |
| --- | --- |
| **Additional Information:** |  |
| **Question** | **Response** |
| **Financial Disclosure**  Enter a financial disclosure statement that describes the sources of funding for the work included in this submission. Review the [submission guidelines](http://journals.plos.org/plosone/s/submission-guidelines#loc-financial-disclosure-statement) for detailed requirements. View published research articles from [*PLOS ONE*](https://journals.plos.org/plosone/) for specific examples.  This statement is required for submission and **will appear in the published article** if the submission is accepted. Please make sure it is accurate. | Yes |
| **Funded studies**  Enter a statement with the following details:   - Initials of the authors who received each award - Grant numbers awarded to each author - The full name of each funder - URL of each funder website - Did the sponsors or funders play any role in the study design, data collection and analysis, decision to publish, or preparation of the manuscript? |  |
| Did you receive funding for this work? |  |
| Please add funding details.  as follow-up to "**Financial Disclosure**  Enter a financial disclosure statement that describes the sources of funding for the work included in this submission. Review the [submission guidelines](http://journals.plos.org/plosone/s/submission-guidelines#loc-financial-disclosure-statement) for detailed | This study was conducted using PM’s start-up funds. The Pima County Health Department provided some funds to aid data collection. The content is solely the responsibility of the authors and does not necessarily represent the official views of the funders. MBN was supported by the National Institutes of Health, Fogarty International Center/ Global Health Emerging Scholars Fellowship (D43TW010540). There was no additional external funding received for this study. The content is solely the responsibility of the authors and does not necessarily represent the official views of the |

| requirements. View published research articles from [*PLOS ONE*](https://journals.plos.org/plosone/) for specific examples.  This statement is required for submission and **will appear in the published article** if the submission is accepted. Please make sure it is accurate. | funders. |
| --- | --- |
| **Funded studies**  Enter a statement with the following details:   - Initials of the authors who received each award - Grant numbers awarded to each author - The full name of each funder - URL of each funder website - Did the sponsors or funders play any role in the study design, data collection and analysis, decision to publish, or preparation of the manuscript? |  |
| Did you receive funding for this work?" |  |
| Please select the country of your main research funder (please select carefully as in some cases this is used in fee calculation).  as follow-up to "**Financial Disclosure**  Enter a financial disclosure statement that describes the sources of funding for the work included in this submission. Review the [submission guidelines](http://journals.plos.org/plosone/s/submission-guidelines#loc-financial-disclosure-statement) for detailed requirements. View published research articles from [*PLOS ONE*](https://journals.plos.org/plosone/) for specific examples.  This statement is required for submission and **will appear in the published article** if the submission is accepted. Please make sure it is accurate. |  |

| **Funded studies**  Enter a statement with the following details:   - Initials of the authors who received each award - Grant numbers awarded to each author - The full name of each funder - URL of each funder website - Did the sponsors or funders play any role in the study design, data collection and analysis, decision to publish, or preparation of the manuscript? |  |
| --- | --- |
| Did you receive funding for this work?" |  |
| **Competing Interests**  Use the instructions below to enter a competing interest statement for this submission. On behalf of all authors, disclose any [competing interests](http://journals.plos.org/plosone/s/competing-interests) that could be perceived to bias this work—acknowledging all financial support and any other relevant financial or non-financial competing interests.  This statement is required for submission and **will appear in the published article** if the submission is accepted. Please make sure it is accurate and that any funding sources listed in your Funding Information later in the submission form are also declared in your Financial Disclosure statement.  View published research articles from  [*PLOS ONE*](http://journals.plos.org/plosone/) for specific examples. | The authors have declared that no competing interests exist. |

| **NO authors have competing interests**  Enter: *The authors have declared that no competing interests exist.*  **Authors with competing interests**  Enter competing interest details beginning with this statement:  *I have read the journal's policy and the authors of this manuscript have the following competing interests: [insert competing interests here]* |  |
| --- | --- |
| * typeset |  |
| **Data Availability**  Authors are required to make all data underlying the findings described fully available, without restriction, and from the time of publication. PLOS allows rare exceptions to address legal and ethical concerns. See the [PLOS Data Policy](http://journals.plos.org/plosone/s/data-availability) and [FAQ](http://journals.plos.org/plosone/s/data-availability#loc-faqs-for-data-policy) for detailed information.  A Data Availability Statement describing where the data can be found is required at submission. Your answers to this question constitute the Data Availability Statement and **will be published in the article**, if accepted. | Yes - all data are fully available without restriction |
| **Important:** Stating ‘data available on request from the author’ is not sufficient. If your data are only available upon request, select ‘No’ for the first question and explain your exceptional situation in the text box. |  |
| Do the authors confirm that all data underlying the findings described in their manuscript are fully available without restriction? |  |
| Additional data availability information: |  |
| **Ethics Statement**  Enter an ethics statement for this submission. This statement is required if | The Institutional Review Board at the University of Arizona reviewed and approved the study (Protocol ID: 2007796226).All participants provided informed consent before participating in any research activities. |

| the study involved:   - Human participants - Human specimens or tissue - Vertebrate animals or cephalopods - Vertebrate embryos or tissues - Field research   Write "N/A" if the submission does not require an ethics statement.  General guidance is provided below. Consult the [submission guidelines](http://journals.plos.org/plosone/s/submission-guidelines#loc-guidelines-for-specific-study-types) for detailed instructions. **Make sure that all information entered here is included in the Methods section of the manuscript.** |  |
| --- | --- |

| **Format for specific study types**  **Human Subject Research (involving human participants and/or tissue)**   - Give the name of the institutional review board or ethics committee that approved the study - Include the approval number and/or a statement indicating approval of this research - Indicate the form of consent obtained (written/oral) or the reason that consent was not obtained (e.g. the data were analyzed anonymously)   **Animal Research (involving vertebrate animals, embryos or tissues)**   - Provide the name of the Institutional Animal Care and Use Committee (IACUC) or other relevant ethics board that reviewed the study protocol, and indicate whether they approved this research or granted a formal waiver of ethical approval - Include an approval number if one was obtained - If the study involved *non-human primates*, add *additional details* about animal welfare and steps taken to ameliorate suffering - If anesthesia, euthanasia, or any kind of animal sacrifice is part of the study, include briefly which substances and/or methods were applied   **Field Research**  Include the following details if this study involves the collection of plant, animal, or other materials from a natural setting:   - Field permit number - Name of the institution or relevant body that granted permission |  |
| --- | --- |
|  |  |
| <strong>Data Availability</strong><br>  <br>Before publication, Authors are required to make fully available and without restriction all data underlying their findings. Sharing the data underlying your findings increases the visibility, credibility, and impact of your work, while supporting transparency and future scientific discovery. Please see our <a href="<https://journals.plos.org/plosone/s/d> ata-availability" target="_blank">PLOS Data Policy page</a> for detailed information on this policy.</p>  <br>A <strong>Data Availability | Data cannot be shared publicly because they contain potentially identifying or sensitive patient information. Data are available from the University of Arizona Institutional Review Board (contact via [vpr-irb@arizona.edu](mailto:vpr-irb@arizona.edu)) for researchers who meet the criteria for access to confidential data. |

| Statement</strong>, detailing where the data can be accessed, is required at first submission. Insert your Data Availability Statement in the box below.</p>  <br>Please see the <a href="<https://journals.plos.org/plosone/s/s> ubmission-guidelines#loc-data-reporting" target="_blank">data reporting</a> of our submission guidelines for instructions on what you need to include in your Data Availability Statement. Examples of acceptable Data Availability Statements are included here for reference:</p>  <ol>  <li>The minimal data set [and accompanying code, where generated] is available at [repository name] via [DOI hyperlink]</li>  <li>No data was generated by this study. The following existing data sources were used: [dataset name] from [dataset location] available via [web address or DOI hyperlink].</li>  <li>For Study Protocols: No datasets were generated or analysed during the current study. All relevant data from this study will be made available upon study completion.</li>  </ol>  <br><i>PLOS allows rare exemptions to address legal and ethical concerns. If you have legal or ethical restrictions, please use the box below to detail these in full sentences for the Journal team to consider.</i></p>  <br>This statement will be typeset if the manuscript is accepted for publication.</p> |  |
| --- | --- |

Manuscript	[Click here to access/download;Manuscript;Ellis et al Manuscript](https://www2.cloud.editorialmanager.com/pone/download.aspx?id=44690854&guid=2384d3b3-d27e-4e78-b62c-d81beec248a7&scheme=1)[<https://www2.cloud.editorialmanager.com/pone/download.aspx?id=44690854&guid=2384d3b3-d27e-4e78-b62c-d81beec248a7&scheme=1> Ver 4.8_clean.docx](https://www2.cloud.editorialmanager.com/pone/download.aspx?id=44690854&guid=2384d3b3-d27e-4e78-b62c-d81beec248a7&scheme=1)

1

### 1 COVID-19 Vaccine Confidence Among Adults of Pima County using the NIMHD

2 **Minority Health and Health Disparities Research Framework: A Qualitative Analysis**

3

### 4 Short Title: COVID-19 Vaccine Confidence in Pima County Adults: A Qualitative

5 **Study Using the NIMHD Framework**

6

7 Kim Ellis^1^, MSc, MPH; Pamela Hall^2^, PhD; Latrisha Robinson^3^, MSc; Sara Ruiz^1^ Rn-
8 MSN, MPH; Maiya G Block Ngaybe^1^, MPH, PhD; Christopher Lessenich^1^, MPH;
9 Namoonga M. Mantina, MsPH, MBA, PhD^1^; Carlos M Perez-Velez^4,5^, MD; Beatrice
10 Krauss^5^, PhD; Purnima Madhivanan, MBBS, MPH, PhD * 1,7

11

12 ^1^ Mel & Enid Zuckerman College of Public Health, University of Arizona, Tucson, Arizona,
13 USA
14 ^2^ Department of Psychology, College of Arts and Sciences, Barry University, Miami
15 Shores, Florida, USA
16 ^3^ Department of Psychology, College of Arts, Sciences & Education, Florida International
17 University, Miami, Florida, USA
18 ^4^ Division of Epidemiology, Pima County Health Department, Tucson, Arizona, USA
19 ^5^ Division of Infectious Diseases, College of Medicine, University of Arizona, Tucson,
20 Arizona, USA
21 ^5^ Psychology Department, College of Science, University of Arizona, Tucson, Arizona,
22 USA
23 ^6^ Public Health Research Institute of India, Mysore, India

24

### 25 Corresponding Author

26 Purnima Madhivanan, MBBS, MPH, PhD
27 Department of Health Promotion Sciences, Mel & Enid Zuckerman College of Public
28 Health
29 University of Arizona,
30 1295 N Martin Avenue, PO Box 245209, Tucson, AZ 85724-5209
31 (520) 621-5730; [pmadhivanan@arizona.edu](mailto:pmadhivanan@arizona.edu)

32

33	**Word count:** 5494

34

35 **Keywords:** Vaccination Refusal, COVID-19 vaccine, Patient Acceptance of Health Care,
36 Trusted Sources of Information, Health Disparities

### 37 Abstract

38 **Introduction**
39 Vaccinations have been established as one of the most effective strategies to
40 prevent the spread of infectious diseases. However, vaccine hesitancy has been a
41 growing issue that needs to be monitored and understood to ensure successful
42 promotion. Historic structural, social, and health disparities have been demonstrated to
43 have significant impact on many health outcomes including vaccination uptake. This study
44 applied the National Institute on Minority Health and Health Disparities Research
45 Framework to examine factors influencing COVID-19 vaccination intention during the
46 COVID-19 pandemic among adults in Pima County, Arizona.

### 47 Methods

48 Between 22 Aug and 05 Dec 2020, 10 focus group discussions stratified by
49 race/ethnicity, gender and language spoken (FGDs) were conducted virtually among 56
50 adult residents of Pima County, Arizona. After transcription and translation of FGDs
51 conducted in Spanish, transcripts were organized by demographic characteristics and
52 coded in Dedoose software by four independent coders. The NIMHD Framework’s
53 Domains of Influence were used as *a priori* codes in the thematic analysis. All participants
54 completed an informed consent process prior to participation.

### 55 Results

56 Of the 56 participants who took part in the ten focus groups, 27% self-reported
57 being Hispanic or Latinx, 25% were White/non-Hispanic, 9% were Black, and 4% were
58 Asian. The preferred language was most often reported as English. Unique challenges
59 for each demographic group emerged from this analysis, such as language barriers
60 among Hispanic participants, historical mistreatment concerns among Black participants,
61 and conflicting political messages for White participants. Cultural norms, family
62 influences, and attitudes toward autonomy versus community responsibility further
63 shaped vaccine decisions. Effective communication about vaccine safety, clinical trials,
64 and public health messaging were identified as critical to addressing mistrust and vaccine
65 confidence across diverse groups.

### 66 Conclusion

67 Major themes in this study that predict vaccine intention include health literacy
68 challenges, misinformation, mistrust in healthcare systems, and the desire to protect
69 others. Understanding the behaviors and decisions behind vaccination acceptance and
70 receipt is important for developing effective vaccine communication strategies. Identifying
71 health disparities driving vaccination hesitancy may help guide future strategies and
72 policies to improve vaccination rates, especially among minority groups.

### 73 Introduction

74 The World Health Organization (WHO) recognized vaccine hesitancy as one of
75 the top ten global health threats of 2019 since inadequate vaccination rates allow a
76 disease to spread and create new variants [1]. In recent years, COVID-19 has been
77 listed as the third leading cause of death in the United States (US), resulting in more
78 than 6.5 million hospitalizations and about 1.2 million deaths [2].
79 Despite high morbidity and mortality rates, acceptance of COVID-19 vaccine
80 remained low after its initial release. The COVID-19 vaccination program was launched
81 on December 14th, 2020, in the US [3]. Ndugga et al found that 48% of Caucasians had
82 received the vaccine by July 2021, while only 38% of Blacks and 41% of Hispanics had
83 received the vaccine [4]. A study by Nikolovski et al found that 91% of adults aged 65
84 and older were willing to get vaccinated against COVID-19 [5], and Gatwood et al found
85 an acceptance rate of 45.9% in adults aged 18-64 [6]. A review found that COVID-19
86 vaccination rates in the US among various populations ranged from a low of 12% to a
87 high of 91% [7]. The same paper also mentioned that Arizona had recorded one of the
88 highest rates (76%) of COVID-19 vaccine acceptance in the USA [7].
89 The Arizona Department of Health Services (ADHS) noted that the vaccine was
90 available to all adults in Arizona (AZ) in December, 2020 [8]. By April, 2021, about 40%
91 had received at least one dose [8], higher than the national average rate of 29.5% at the
92 time [9]. Disparities among minority races and ethnicities in COVID-19 vaccination
93 coverage persisted nationwide in vaccine rates [10] as well as in Arizona [11].
94 Multiple factors have been found to be associated with an individual’s decision to
95 accept, refuse, or hesitate to get vaccinated, including religion, cultural background,
96 socioeconomic status, political beliefs, the nature of vaccine communication, media
97 consumption, past experiences with vaccination, sociodemographic characteristics and
98 perceived benefits and risks [12-14]. Communities of Color (CoC) have been shown to
99 face disproportionate rates of infection and death due to COVID-19 [15]. Vaccine
100 hesitancy rates have been shown to be the highest among minority groups in the US [6,
101 16, 17]. For example, Ratnayake et al, found that Blacks had double the likelihood of
102 vaccine hesitancy compared to Whites [14]. Similar results were reported by Savoia et al,
103 with rates of vaccine hesitancy among Blacks being 70% in comparison to 22% for Whites
104 [17]. A study by Williams et al, revealed that vaccination coverage is lower among all
105 racial and ethnic minority groups except Asians when compared to Whites [18]. It has
106 also been shown that Hispanics and Blacks had the highest rate of participants that were
107 undecided about vaccination or less likely to be fully vaccinated [4, 16, 18]. While Savoia
108 et al found similar results, their results differed in that they found that Hispanics were
109 slightly less likely to be vaccine hesitant when compared to other races [17]. Though
110 White communities generally fare better across many health measures compared to other
111 racial and ethnic groups, disparities still exist in White communities. Several social
112 determinants of health that impact White communities include socioeconomic status,
113 neighborhood, and built environment [4].
114 In this paper, we analyzed COVID-19 vaccine decision-making at the time when
115 the vaccine was in the initial stages of its rollout. Relying on a health disparities research
116 framework, this qualitative study examined the decisions that impacted vaccine receipt,
117 acceptance, and hesitancy in CoC and White communities in Pima County, AZ.

### 118 Methods

119 *Participants*
120 The study took place in Pima County, Arizona during the COVID-19 pandemic’s
121 onset with recruitment done between 22 Aug and 05 December, 2020, prior to vaccine
122 rollout in the United States. We conducted 10 focus group discussions (FGDs) with a total
123 of 56 participants. Approximately 27 additional participants who had been recruited for
124 the study failed to show up for their scheduled FGD for unknown reasons. We used
125 purposive	sampling	methods	for	our	study	to	ensure	representation	across
126 race/ethnicities (White, Latinx and Black), Language (English and Spanish), and gender
127 (Male and Female) from the networks of the study team. All participants provided
128 informed consent before participating in any research activities. The Institutional Review
129 Board at the University of Arizona reviewed and approved the study (Protocol ID:
130 2007796226).
131 We recruited participants to the study via social media, or email using ethical review
132 board-approved recruitment scripts and digitally shared flyers. Participants often
133 belonged to the same or similar social groups, such as an African American wellness
134 group, or a group of college students familiar with a member of the research team, or a
135 Hispanic activist group. Study team members would contact potential participants to invite
136 them to participate in the study. Participants were included if they were above 18 years
137 of age and were living in Pima County at the time of the study. Upon recruitment,
138 participants were introduced to the research study and its purpose, fore-shadowing
139 informed consent.
140 *Interview Guide Development*
141 The semi-structured focus group discussion guide and prompts were developed
142 iteratively by the team by using established qualitative methods [19]. The team conducted
143 a pilot FGD internally before using the guide with participants; no repeat interviews were
144 conducted.
145 *Interview Setting*
146 FGDs were held on an online virtual Zoom platform and lasted between 60 to 90
147 minutes. All participants were initially let into the Zoom room individually, while others
148 were in the waiting room, to explain that they could change their name displayed on the
149 Zoom call and hide their faces if they desired anonymity on the call. All participants
150 completed a verbal informed consent process prior to the FGD. All sessions were audio
151 and video recorded. A notetaker from the research team attended each session to take
152 notes and provide support for the facilitator during each focus group.
153 *Data Management*
154 Debriefing sessions were held at the end of each FGD to discuss thematic
155 saturation and notes for improvement in future sessions. Transcripts were not returned to
156 participants for comment or correction. The automatic transcription feature of Zoom was
157 used to generate initial transcripts for those held in English and then reviewed while
158 listening to the audio recording by a research team member. Students who were studying
159 Spanish translation were hired to transcribe and translate FGD sessions held in Spanish.
160 For further details about the participants and the recruitment process and the analysis of
161 data collected about seasonal influenza-related outcomes, refer to the article by Mantina
162 et al [20].
163 *Reflexivity Statement*
164 The interviewers were a male Hispanic medical care provider practicing at the
165 Pima County Health Department (CPV), a male Black PhD student (FM), a female
166 professor of Indian origin, who was also the PI of the project with a PhD and medical
167 degree (PM), and a female mixed race MPH student who was acting as study coordinator
168 (MBN). All had some experience in qualitative research and received some additional
169 training from the study team to ensure consistency. BK consulted on qualitative methods,
170 interview design, and analysis. PM and CPV were the members of the team who
171 conceptualized the project and determined that it was important to conduct this research
172 to explore the evolving issues of a possible COVID-19 vaccine and possible factors that
173 could be associated with future vaccine acceptance to inform a promotion strategy for the
174 Pima County Health Department.
175 The qualitative analysis team included the lead MPH student (KE), two PhD
176 students (MBN, LR), and a professor (PH). One of the PhD students (MBN) had been an
177 interviewer, had previous experience in qualitative research, and served as the research
178 project coordinator. One of the PhD students (LR) had limited experience in qualitative
179 research and was studying Applied Social and Cultural Psychology. The professor of
180 psychology (PH) who helped with the data analysis is a PhD holder and experienced
181 researcher in social psychology and minority mental and public health research.	All
182 analysts identified as female, two as black (LR, PH), one as mixed race (MBN), and one
183 as white (KE). Researchers who provided feedback and guidance on the qualitative
184 research included one white male MPH student with more than three years of public
185 health research experience (CL), a black female PhD student with at least 3 years of
186 experience in qualitative research (NM), and a white female researcher with decades of
187 experience in community-based qualitative research (BK).
188 *Theoretical Framework*
189 The National Institute on Minority Health and Health Disparities (NIMHD)
190 framework accounts for social factors such as education and income [21]. In this study,
191 we utilized this framework since it focused on Black, White, and Latinx communities.
192 The NIMHD research framework is broadly used in the literature to explain the root
193 causes of health disparities and social determinants of health in CoCs [21]. Building on
194 the ideas of the socio-ecological model’s levels and concepts from a similar health
195 disparities research framework from the National Institute of Aging (NIA), the NIMHD
196 research framework is a “tool for conceptualizing and depicting the wide array of
197 determinants that promote or worsen minority health or cause, sustain, or reduce health
198 disparities” [21]. The framework was intended to help guide health disparities research by
199 demonstrating different factors at different levels of society that may contribute to the risk
200 and resilience factors related to health equity and inequities. The framework has two
201 axes, with domains of health (biological, behavioral, physical and built environment, socio
202 cultural environment, and healthcare system) depicted on the x-axis and levels of
203 influence on health (individual, interpersonal, community, and societal) mapped onto the
204 y-axis. This visual representation of the NIMHD framework also serves as a guide for
205 research on health disparities, though it has yet to be used widely. It will be referred to as
206 “the framework” henceforth. There is a more detailed description of the framework in

### 207 Appendix 1.

208

209 *Data Analysis*
210 The qualitative analysis team began by familiarizing themselves with the FGD
211 transcript data. A framework content analysis was using primarily a deductive approach
212 when identifying themes, mapping them onto the framework as predetermined codes,
213 which were applied in Dedoose. The team created a codebook of the Domains of
214 Influence with definitions for each domain, including biological, social, psychological, etc.
215 They collaboratively created matrices with definitions and examples to easily determine
216 which type of data would be coded under each Domain of Influence. The Levels of
217 Influence were included within each definition of the Domain codes. Emerging themes
218 which may not have easily fit clearly under one Domain were additionally identified
219 inductively and included as their own separate codes. When developing the codebook,
220 the team discussed the overlap in the framework and determined through consensus
221 which information was included within each code.
222 The transcripts were each coded by mapping the content from each transcript from
223 each group onto the framework matrix by one person on the team. Subsequently, a
224 second coder from the team reviewed the matrices for each group to verify for accuracy,
225 taking notes and making tracked changes to the codes as necessary, reviewing the
226 transcript as they reviewed the coded data. The team held regular discussions to discuss
227 the codes that each team member applied to the transcript and resolved conflicts as a
228 group through iterative discussion and consensus. During these meetings, suggested
229 changes to the way the data were organized or “coded” onto the NIMHD framework
230 matrices were discussed. We did not calculate inter coder reliability because conflicts
231 were consistently resolved as a group and the dataset was smaller [22, 23]. In order to
232 ensure sufficient rigor, many discussions were held to ensure data were coded
233 accurately, iteratively until all team members were satisfied. Each team member
234 additionally took notes on the transcripts to further identify meanings, patterns, and
235 comparisons among the data. The last step of the analysis involved in-depth discussion
236 of each code applied in the transcript, assessing existing patterns and determining
237 relevance to sociocultural factors, personal experience, medical history, and other
238 relevant factors.
239 To display the examples of the themes that emerged, tables were created for each
240 of the focus groups. While analyzing the data and developing themes, one additional code
241 was added inductively to capture politics. The team created matrices and wrote
242 summaries of findings for each racial/ethnic category of focus group and used these to
243 create the results presented in this manuscript. See **Supplemental File 1** for the initial
244 summaries made from each racial/ethnic category of focus groups. The codebook created
245 based on the framework and its domains and corresponding definitions are included in
246 **Appendix 1.** The Consolidated criteria for reporting qualitative research (COREQ) was
247 used to inform how information was reported in this manuscript.

248

### 249 Results

250 Of the 56 participants who took part in the ten focus groups, 34% were female.
251 While 27% self-reported being Hispanic or Latinx, 25% were White, 9% were Black, and
252 4% were Asian, noting that some participants may have fallen under multiple categories.
253 The preferred language was most frequently reported to be English (24, 43%) followed
254 by Spanish (11, 20%). Demographic information was not reported for almost half of the
255 participants (21, 38%). Although participants were grouped into focus groups based on
256 their social groups, since they did not disclose their demographic information consistently,
257 we will not report it at an individual level. It is important to note that some groups had
258 small representation, limiting the interpretation of subgroup comparisons. The themes
259 identified and mapped onto the NIMHD framework are reported in Table 1.

260

### 261 Table 1: NIMHD Framework-Aligned Themes on Vaccine Perceptions and

262 **Calculated Risks by Racial/Ethnic Group**

263

| **Domain** | **Black Focus Groups** | **Hispanic/Latino Focus Groups** | **White Focus Groups** |
| --- | --- | --- | --- |
| **Biological Domain** | Family vulnerabilities (e.g., heart conditions) | Fear of infecting elderly parents | Individual immunity, calculated risk |
| **Behavioral Domain** | Concerns about long-term effects, protecting others | Self-isolation and strict precautions | Social responsibility, protecting others |
| **Physical/Buil t Environment** | Environmental hygiene (e.g., mask, gloves, cleaning car) | Room isolation, household distancing | Institutional concerns (school outbreaks) |
| **Sociocultural Environment** | Historical mistrust (Tuskegee) | Community-based trust and privacy | Trust in close circles, confusion over messaging |
| **Healthcare System** | Distrust due to testing bias & lack of representation | Conflicting medical advice & uncertainty | Concerns about rushed trials, desire for clear information |
| **Politics (Inductive Theme)** | Mistrust of CDC, trust in local health officials, distrust of political media | Belief that COVID is politically motivated | Distrust of political media, concern about rushed rollout |

264

265 *Biological Domain*
266 Participants across racial groups highlighted biological factors affecting their
267 vaccine-related	decisions.	Biological	vulnerabilities,	such	as	chronic	conditions,
268 significantly shaped attitudes and behaviors towards vaccination. For instance, a White
269 male participant was concerned about the possibility of being infected while in public
270 areas,
271 *“I think my risks of getting it are high. And I think I take precaution, and I am pretty*
272 *proactive on taking care of myself. But even with me taking those steps and*
273 *working, for I have the privilege of working from home, I think I still-- anytime you're*
274 *in public, I think you're putting yourself at risk. So, I think there's a high probability*
275 *of either encountering someone who has COVID-19 or is either connected. So, I*
276 *think- my framework that I use is anytime you're out in public or outside you're at*
277 *risk of getting it.” (p25)*
278 Black participants expressed awareness of familial vulnerabilities, illustrating heightened
279 caution due to chronic illnesses among family members:
280 *“So, I just try to protect my grandchildren and my daughter, who has a bad heart.*
281 *I tried to pick those, you know, things like that.” (p29)*
282 A Hispanic participant similarly emphasized maintaining health and protecting loved ones:
283 *“Immediately have hand sanitizer, or wash [my daughter’s] hands, and don’t touch*
284 *anything, don’t touch your face […] You don’t pay attention to what you are going*
285 *to do because you’re watching them. Because there is fear-- my fear is that she*
286 *infects my parents because they are vulnerable.” (p36)*
287 Participants collectively recognized that individual health directly influenced decisions to
288 vaccinate for broader community protection, with a Black male participant noting
289 communal responsibility said:
290 *“I feel like it’s just my responsibility to the rest of the community” (p53).*
291 Black and Hispanic participants prominently emphasized familial and community
292 biological vulnerabilities, contrasting with White participants, who focused more on
293 individual health and robust immunity.

294

295 *Behavioral Domain*
296 Behavioral factors influencing preventive practices and vaccine hesitancy were
297 articulated across groups. Hispanic participants frequently described cautious preventive
298 measures at home and working on proactive strategies*.* One Hispanic participant added,
299 *“What I would do is isolate myself, you understand me, from my family as well to*
300 *not get other people sick and that’s what we are doing right now” (p50).*
301 A Black participant expressed significant concerns regarding vaccine uncertainties,
302 stating,
303 *“Long term health effects…they can't tell us what it does to us five years in the*
304 *future, until it starts popping up five years in the future. So that's the biggest worry”*
305 *(p53).*
306 White participants framed their decisions around familial responsibilities, stating,
307 “*Yeah, protect other people...my motivator is to protect them…it's my social*
308 *responsibility not to be a vector, if possible” (p9)*.

| 309 | Across groups, community responsibilities emerged as a common theme. Societal |
| --- | --- |
| 310 | mandates played a significant role in shaping behaviors, particularly in cases where |
| 311 | vaccination was required for immigration or employment purposes. Behavioral responses |
| 312 | differed across racial and ethnic groups in our study: Black and Hispanic participants |
| 313 | tended to express more cautious approaches influenced by uncertainties around the |
| 314 | vaccine, while White participants more frequently emphasized responsibility to protect |
| 315 | others in their social and family circles: |
| 316 | *“But that's like how I think about it like I don't want to get my parents sick. You* |
| 317 | *know my grandmas are both 87 they would die” (p18).* |
| 318 |  |
| 319 | *Physical/Built Environment* |
| 320 | Physical and built environmental contexts influenced health behaviors among |
| 321 | participants. A Black participant particularly emphasized rigorous environmental hygiene |
| 322 | practices as critical preventive measures: |
| 323 | *“I don't go out to the malls and family gatherings and stuff where there's a lot of* |
| 324 | *people, so therefore I go out, mainly when there's an essential reason and I try to* |
| 325 | *do all those things. So, to distance myself, I use a mask and gloves if necessary.* |
| 326 | *Then I pull them off and dispose of those gloves there at the store or where I am.I* |
| 327 | *don't want any germs in my car. And when I pick up my groceries, I call them and* |
| 328 | *pick them up from the store. I have to put it in place into my trunk because again I* |
| 329 | *want nothing near me.” (p32)* |
| 330 | Similarly, a Hispanic male participant detailed significant home isolation strategies to |
| 331 | prevent viral transmission within families, describing actions such as |

332 *“She [my daughter] calls us from her work that she did not want to have direct*
333 *contact with us, because a person came out positive, then what we did was that*
334 *we started fixing the room and leaving the house to go live with another daughter*
335 *in the same place.” (p55)*
336 A	few White	male	participants	briefly	mentioned	concerns	about	professional
337 environments like schools, acknowledging risks within institutional settings, such as,
338 *" New cases in Tucson are being driven by the university student population,*
339 *particularly the large student housing complexes. And so, I have concern for, for*
340 *my colleagues who are teaching and interacting with those students.” (p24)*.
341 This domain revealed focused personal and familial environmental adjustments
342 predominantly among Black and Hispanic participants. Black and Hispanic participants
343 provided more detailed descriptions of proactive ecological strategies than White
344 participants, who mainly acknowledged broader institutional environmental risks.
345 Participants were more concerned about bringing the virus into their physical/built
346 environments.

347

348 *Sociocultural Environment*
349 Sociocultural factors profoundly influenced participant perspectives, including
350 historical mistrust, cultural norms, and social networks. Black participants notably
351 referenced historical injustices, exemplified by the Tuskegee Syphilis Study. One
352 participant emotionally stated,
353 *“Getting ready to get a little deep for a moment. Oh, the Tuskegee Syphilis Study*
354 *done on African American. I want to say with all my goodness, maybe the 20s,*
355 *30s, 40s. I don't know the exact date, but that sticks in my mind, of giving People*
356 *of color placebo … Oh, it makes me cry sometimes because it's so sad… I am*
357 *hesitant to go to the doctors. There’s been [a] long line and a long history of African*
358 *American people, whether we are older or younger, or female or male not being*
359 *treated right in the health care system.” (p43)*
360 Some Hispanic participants placed significant trust in local communities, describing
361 cultural privacy and preventive practices. As one participant said,
362 *“I think that in the Hispanic environment, or so to speak, there is a little more*
363 *privacy in terms of people taking into account... Well, I am talking about the circle*
364 *closest to me, and they take into account that we must follow the rules precisely*
365 *so as not to become infected. For example, there are not so many parties, I don't*
366 *know if they go to so many bars and all that.” (p55).*
367 A White male participant emphasized interpersonal trust within immediate social groups
368 as pivotal:
369 *“I think … from my perspective, I think we've kept our circle pretty tight. And I think*
370 *we've kind of been- especially during these times- we've kept it... Well, I think, trust,*
371 *trust, trust in each other. And I think we've been hanging out a lot with like-minded*
372 *people that kind of see this in the same light. So, trust really has played a big part*
373 *in who we spend time with, who we hang out [with]. So, I think that has been a*
374 *huge part in our, in our circle.” (p25)*
375 Societal influences also emerged clearly, with participants across groups
376 expressing deep frustration over politicized and inconsistent health information: *“[T]hey*
377 *confuse people more like that, and they scare them more because they are the experts;*
378 *they should be constant” (p37).*	Some Black	participants highlighted systemic
379 discrimination and historical mistrust. Hispanic participants often demonstrated strong
380 community-centered trust, and White participants often focused on confusion due to
381 societal misinformation.
382 These findings illustrate how vaccine decision-making was shaped not only by
383 individual beliefs but also by interpersonal trust, community norms, and broader societal
384 narratives. Within the NIMHD framework, sociocultural influences operated across levels:
385 family and close social networks shaped perceived responsibility, community histories
386 shaped mistrust, and politicized national discourse shaped confidence in public health
387 institutions.

388

389 *Health Care System*
390 Participants expressed widespread skepticism and mistrust regarding healthcare
391 systems, highlighting communication, accessibility, representation, and care quality
392 concerns. Some Hispanic participants notably voiced confusion due to inconsistent
393 messaging from healthcare authorities:
394 *“The ones who did the exam said she should stay seven more days after the test.*
395 *The pediatrician tells me that she should stay ten more days. Who is right? They*
396 *told me, ‘Well, in reality we don’t know.’ So, if you are trusting someone and they*
397 *tell you ‘well we don’t know because it’s new.’ Who are you going to trust?” (p36)*
398 Some Black participants articulated specific apprehensions regarding different cultural
399 representations in vaccine trials*.* A participant expressed broader mistrust:
400 *“Medications are done on white people and maybe white men mostly. Yeah, just*
401 *save it. And so, we are in the minority when they are testing out these new*
402 *medications, and then they are being used on you. Used in a negative way. A lot*
403 *of work is done in Caribbean and African countries, and people are not well*
404 *informed.” (p45)*
405 A White participant emphasized concerns about rapid vaccine development:
406 *“One of my concerns is because it has a profit motive, but also because it's a race*
407 *right now. Not just cutting corners, but just efficacy may not be measured as*
408 *stringent as it should be. And so, the first-generation vaccines may or may not be*
409 *effective, but they also probably [have] been run through as much rigorous testing*
410 *in order to have a time frame and that worries me.” (p8)*
411 Additionally, another White participant highlighted the need to understand vaccine
412 implications clearly:
413 *“I'd want to know the effects of it. Is it you know, something, you get the vaccine,*
414 *but you still have, you know, some odd percentage or chance of being exposed to*
415 *it and getting it? It may just be mild or? I just want to understand If I did get the*
416 *vaccine, what does that mean as far as risk of exposure, and if I got sick, what that*
417 *means.” (p20)*
418 While mistrust towards healthcare systems was common across all groups, Black
419 participants particularly emphasized representation issues and systemic barriers,
420 Hispanic participants focused on confusion from inconsistent messaging, and White
421 participants emphasized trust in interpersonal healthcare relationships.

422

423 Inductive Theme: Politics
424 In our analysis, we identified a significant theme not captured in the NIMHD
425 Research Framework: the role of the political environment in influencing COVID-19
426 vaccine hesitancy. Participants across racial and ethnic groups expressed concerns
427 about political interference potentially compromising the integrity of vaccine development.
428 Some of the Hispanic/Latino participants felt that COVID-19 was fake, and being used by
429 the government to cover something up and expressed a fear of what was being put into
430 the vaccine.	Participants from this group tended to not trust President Trump and
431 reported that if the vaccine were to come out before the election, they would not trust the
432 COVID-19 vaccine. Many participants also felt that scientists were disagreeing with each
433 other, and that countries were competing instead of trying to develop a vaccine that would
434 collectively help humanity.	Similarly, a Hispanic female voiced frustration over the
435 politicization of vaccine efforts, commenting,
436 *“I see more political taint in the countries, more than the desire… for curing this*
437 *sickness” (p4).*
438 Some of the Black participants felt that those in power were able to influence what
439 is being said in the media. Participants in this group mentioned that the COVID-19 vaccine
440 was being too politicized. Distrust in federal health institutions was common. However,
441 many did trust their local government officials. A Black mixed gender group participant
442 noted,
443 *“I would not trust the CDC, the NIH, or any public health [officials] that he has*
444 *control over… There's just too much politics and political pressure. But at the local*
445 *level, like Dr. Garcia…I trust him…”* (p30).
446 Some of the White participants felt that more people would be willing to receive the
447 COVID-19 vaccine if information was given via a non-political platform. They note that
448 CNN, Fox News and MSNBC are very political. In line with the Hispanic/Latino and Black
449 participants, White participants also felt that the vaccine was being rushed, and that
450 President Trump was using the vaccine to grow his popularity.
451 *“I think it is completely politicized and that would be the only reason that it would*
452 *be available is because Trump has gone through all the chains of commands to*
453 *make that happen … in order to grow his base. I'm completely pro vaccine, but I*
454 *really don't think I could get behind that.”* (p18)
455 For some, political views extended to personal beliefs, as shared by a White female
456 participant:
457 *“My father… bought into the idea that COVID was a hoax… mainly because he*
458 *didn’t know anyone who had gotten it”* (p10).
459 This theme highlights the importance of recognizing how political dynamics can shape
460 public trust in health initiatives.
461 While all groups expressed concern about rushed vaccine development, the basis
462 of concern differed. Black participants more often linked vaccine concerns to historical
463 mistreatment and underrepresentation in clinical trials; Hispanic participants emphasized
464 uncertainty, family protection, and inconsistent medical guidance; and White participants
465 more often emphasized political interference, individual risk calculation, and the need for
466 clear evidence. These differences suggest that similar expressions of hesitancy may arise
467 from distinct social and structural contexts.
468 Across domains, vaccine intention was shaped by the interaction of perceived
469 biological vulnerability, behavioral risk management, sociocultural trust, healthcare
470 system mistrust, and political context. Participants did not describe vaccine decisions as
471 isolated individual choices; rather, they framed decisions as negotiated through family
472 responsibility, prior experiences with healthcare institutions, perceived credibility of
473 messengers, and uncertainty about the speed and transparency of vaccine development.
474 These findings suggest that vaccine hesitancy in this sample was best understood as a
475 multilevel process rather than a single attitude or knowledge deficit.

476

### 477 Discussion

478 By applying the National Institute on Minority Health and Health Disparities
479 Research Framework in this study, we were able to highlight many factors that influenced
480 adults in Pima County, Arizona to choose to vaccinate or not to vaccinate against COVID-
481 19. There were many common themes among all FGDs, such as participants choosing
482 to take the flu vaccine due to COVID-19 when they might otherwise have not received the
483 flu vaccine. Another common theme shared across the FGDs was wanting to protect
484 others, especially those close to them. A fear that the COVID-19 vaccine was being
485 developed too fast was also mentioned across all FGDs.
486 Health literacy and misinformation were major concerns mentioned across groups,
487 as participants voiced doubts about the accuracy of vaccine information and were
488 skeptical about the vaccine’s effectiveness and safety due to conflicting messages.
489 Concern about safety and side effects has also been mentioned in the literature [24].
490 Historical and sociopolitical mistrust were also common themes, especially concerning
491 the government’s handling of the pandemic. Mistrust in healthcare systems and public
492 health authorities also surfaced as a significant theme. Washington suggests that many
493 in the Black community and possibly other CoC have a fear of the “white coat” or what is
494 called iatrophobia [25]. Iatrophobia is associated with historical events that create what
495 Grier and Cobbs long ago described as a type of cultural paranoia [26]. Vazquez et al.
496 found that historical, cultural, and social trauma induce fear and mistrust in public health
497 and medical institutions, influencing COVID-19 testing and vaccination decisions among
498 Latinx, Indigenous Americans, and Black Americans [27].	Lastly, attitudes toward
499 behavioral protective practices, such as masking and social distancing, also varied
500 significantly across groups with Hispanic participants, especially women, appearing to be
501 more inclined to view vaccination as part of a comprehensive approach necessary for
502 their family’s safety.
503 While there were some similarities across all the FGDs, we found a few decisions
504 surrounding vaccination that were specific to certain groups. It appeared that White
505 participants noted the importance of effective messaging. They also appeared more likely
506 to discuss nurturing one’s own immunity. In contrast, Black and Hispanics appeared more
507 concerned about protecting friends and family. Black participants at times demonstrated
508 a concern about clinical trials, in particular mentioning worries about if they were ethical
509 and inclusive. They also were the only group to mention the Tuskegee Project as their
510 reason to not trust the medical community, similar to other studies from the literature [24,
511 28]. A qualitative study by Casselman-Hontalas et al found several participants referring
512 to the Tuskegee syphilis study and felt that COVID-19 was being experimented on Blacks
513 [29]. This demonstrates a similar need for better communication from researchers to the
514 public regarding how these trials are being conducted. This emphasizes the importance
515 of communicating everything from how a vaccine works to the extensive research and
516 clinical trials involved in making a new vaccine.
517 As was mentioned in our study by Black participants, faith and religion appeared
518 important to them in this context. One way to build trust in the Black community and
519 increase vaccination rates is to involve those they trust such as faith leaders [30].
520 Participants in our study recommended implementing a faith-based approach, and the
521 first step was to listen and get to know the community [30]. Many Black communities have
522 been shown to trust their pastors in the community; therefore, these pastors may be
523 effective messengers for vaccine-related information [30].
524 Our findings align with established vaccine hesitancy frameworks, including the
525 “3Cs” model of confidence, complacency, and convenience and the expanded “5Cs”
526 model, which includes confidence, complacency, constraints, calculation, and collective
527 responsibility [31, 32]. Participants’ concerns about rushed vaccine development,
528 inconsistent	messaging,	and	political	interference	reflect	challenges	related	to
529 confidence. Their questions about personal risk, immunity, and vaccine safety reflect
530 calculation, while repeated references to protecting family members and community
531 members reflect collective responsibility. However, our findings also suggest that these
532 constructs are experienced through racialized and sociopolitical contexts, particularly
533 among	participants	who	linked	vaccine	concerns	to	historical	mistreatment,
534 underrepresentation in clinical trials, and mistrust of federal institutions.
535 Importantly,	participants’	concerns	should	not	be	interpreted	solely	as
536 misinformation or lack of health literacy. Rather, many concerns reflected reasonable
537 responses	to	rapidly	changing	guidance,	limited	transparency	about	vaccine
538 development, prior experiences of discrimination, and broader political mistrust. This
539 distinction is important because interventions focused only on correcting misinformation
540 may fail if they do not also address trust, representation, and institutional accountability.
541 Taken together, these findings suggest that vaccine communication strategies
542 should be tailored not only by demographic group but also by the underlying source of
543 concern. Messages addressing safety and trial transparency may be particularly
544 important when concerns are rooted in mistrust of research. Language-concordant,
545 family-centered communication may be especially useful when concerns involve
546 protecting elders and navigating conflicting guidance. Nonpartisan, locally grounded
547 messaging may be critical when hesitancy is shaped by political distrust. Across groups,
548 trusted messengers, transparency about uncertainty, and acknowledgment of historical
549 and contemporary inequities may be more effective than information-only approaches.
550 A strength of this study is the use of a nationally recognized framework on health
551 disparities to determine factors affecting vaccine attitudes among a purposively sampled
552 group of participants. Additionally, this is the first article to use the NIMHD framework in
553 the field of vaccine hesitancy to date to our knowledge. This sample provided diverse
554 perspectives from different communities within Pima County. The information is very
555 locally relevant, making the findings internally valid, especially during this unique period
556 during the COVID-19 pandemic. The findings could help to inform future health promotion
557 campaigns for novel vaccines, especially during a pandemic. Additionally, it is important
558 to emphasize the value of this study applying the NIMHD Minority Health and Health
559 Disparities Research Framework to pre-rollout vaccine confidence perceptions in a
560 diverse county-level context, showing how concerns about trust, health literacy, historical
561 marginalization, access, and information environments operate across multiple levels of
562 influence.
563 Nevertheless, we recognize that the study has its limitations. It is important to note
564 that the findings are from data collected prior to vaccine rollout in the United States, and
565 therefore reflect anticipated perceptions, concerns, and stated intentions under
566 uncertainty rather than actual vaccine uptake behavior. The data were not collected with
567 the NIMHD research framework in mind, which may mean that some of the information
568 related to the framework may have been missing not due to the participants not
569 mentioning it, but rather due to the data collection tool and facilitator not being oriented
570 towards the framework and its domains. Additionally, there was some possibility of
571 misinterpretation of the data, since the analysts were mostly not involved with the data
572 collection, member checking was not performed due to the delay of the analysis post data
573 collection, and many were not from Pima County as well. We acknowledge that formal
574 back-translation was not conducted for Spanish focus groups, which may have affected
575 nuance in interpretation. Zoom and technological issues additionally made it challenging
576 for some participants to actively and comfortably engage in discussion which may have
577 made it more challenging to have free and open dialogue or share accurate and complete
578 information. The format may also have introduced digital divide bias, which could have
579 caused a selection bias. This discomfort with the new technology, changing the format of
580 the discussions from a focus group discussion to more of a group interview format,
581 unintentionally, and also additionally making it more challenging to collect socio
582 demographic data leading to some gaps in the demographic data collected. We also had
583 a limited sample size, so while we made comparison between racial ethnic groups and
584 participants, these participants do not represent everyone with the same race or ethnicity
585 and so it is important to note that findings may not be generalizable. No member checking
586 was conducted in this study which limits its validity, but this was not feasible since this
587 was a secondary analysis conducted on the data years after the data were collected so
588 the participants themselves would be removed from their thoughts from this specific time
589 in their lives and therefore it would not have made sense to conduct. One final limitation
590 of this study is that the use of a disparities framework may have made the analysis biased
591 towards trying to find disparities rather than recognizing similarities too.
592 Scientists at the National Institute on Minority Health and Health Disparities are
593 devoted to addressing and conducting research on health disparities and inequities. [33].
594 To help bridge the gap between health disparities and to improve vaccination uptake
595 among minority populations, it will be important to have access to data with accurate
596 reporting on vaccination status. Gaps in vaccine uptake will persist until we work towards
597 addressing the health disparities and the drivers behind them. Future studies in Pima
598 County may benefit from a follow-up study to determine how attitudes may have changed
599 regarding these vaccines post-pandemic. It would additionally be important to conduct
600 similar studies on other important emerging vaccines such as the human papillomavirus
601 (HPV), human immunodeficiency virus (HIV) and malaria vaccines to determine how
602 vaccine attitudes vary depending on the type of vaccine.

603

### 604 Conclusion

605 This study adds to the health disparities literature, demonstrating how the COVID-
606 19 pandemic exacerbated health disparities in this country. It is vital that policies should
607 address socioeconomic factors to help reduce these disparities [18]. A paper by Strully et
608 al mentioned that some states had put into place strategies to increase equity while
609 distributing vaccines [28]. However, they note that for this to work, stakeholders need to
610 engage the community and gain their trust [28]. In summary, the driving forces behind
611 vaccine hesitancy may be better understood when categorized into behavioral, biological,
612 sociocultural, and healthcare, and political domains. By applying the NIMHD research
613 framework lens to vaccine hesitancy, it is apparent that there are many opportunities for
614 policymakers and other stakeholders to implement interventions that address different
615 racial/ethnic issues among vaccine uptakes. We encourage future studies to explore the
616 intersectionality of different sociodemographic variables and their impacts on health
617 disparities regarding vaccine hesitancy.

618

619

620

621

622

623

624

625

626

627

628

629

630

631 **Acknowledgements:** We recognize and acknowledge all of the hard work of the entire
632 Alliance for Vaccine Literacy team, including: Brianne Vaught, Sage Vu, Meccah Jarrah,
633 Forest Melton, Veena Raghuraman, Kerry Johnson, Adria Vasquez, Gloria Villa Barbosa,
634 Shailesh Advani, Benjamin Pope, Abidemi Olubukunmi Okechukwu, Anna Karina Ahmed,
635 Anna Teresa Valencia, Ava Neddermeyer, Beatrice Krauss, Bianca Patricia Carrasco,
636 Brianna Catherine Rooney, Carlee May-Qing Bettermann, Cori Cantin, Danielle
637 Hutchens, Deepa Katuri, Dominique Marie Jordan, Elena Hortencia Campos, Griselda
638 Ruiz-Braun, Harrison Schmitt, Jacob Marczak, Kai (Xiao) Glahn, Karl Krupp, Kelly
639 Wentzel, Lady Dorothy Avila Elli, Mary Kinkade, Maryam Reyad, Miguel Lopez, Pati
640 Monterrey, Paula Garcia, Priscilla McGrath, Purnima Madhivanan, Rachel Cummings,
641 Rachel Zambrano, Ryan Jasareno Gonzales, Samantha Joanne Werts, Sonja Velickovic,
642 Stephanie Mallahan, Riley Sena, Kate Chambers, Kaela Liddle, Katie Donovan, Makayla
643 Lang, Nadia Flores, Martha Moore-Monroy, and Elsa Claire Bautista.

### 644 Funding

645 This study was conducted using PM’s start-up funds. The Pima County Health
646 Department provided some funds to aid data collection. The content is solely the
647 responsibility of the authors and does not necessarily represent the official views of the
648 funders. MBN was supported by the National Institutes of Health, Fogarty International
649 Center/ Global Health Emerging Scholars Fellowship (D43TW010540). There was no
650 additional external funding received for this study. The content is solely the
651 responsibility of the authors and does not necessarily represent the official views of the
652 funders.

653

654

### 655 References

656 1.	Organization WH. Top Ten Threats to Global Health in 2019WHO 2019 [cited 2025].
657 Available from: [https://www.who.int/news-room/spotlight/ten-threats-to-global-health-in-2019.](https://www.who.int/news-room/spotlight/ten-threats-to-global-health-in-2019)
658 2.	Prevention CfDCa. Trends in United States COVID-19 Deaths, Emergency Department
659 (ED) Visits, and Test Positivity by Geographic Area: Center for Disease Control and Prevention;
660 2025. Available from: [https://covid.cdc.gov/covid-data-tracker/?#trends_totaldeaths_select_00.](https://covid.cdc.gov/covid-data-tracker/?&trends_totaldeaths_select_00)
661 3.	Prevention CfDCa. CDC Museum Covid-19 Timeline: Center for Disease Control and
662 Prevention;	2025	[cited	2025].	Available	from:	[https://www-cdc-](https://www-cdc-gov.translate.goog/museum/timeline/covid19.html?_x_tr_hl=id&_x_tr_pto=tc&_x_tr_sl=en&_x_tr_tl=id)
663 [gov.translate.goog/museum/timeline/covid19.html?_x_tr_hl=id&_x_tr_pto=tc&_x_tr_sl=en&_x_tr](https://www-cdc-gov.translate.goog/museum/timeline/covid19.html?_x_tr_hl=id&_x_tr_pto=tc&_x_tr_sl=en&_x_tr_tl=id)
664 [_tl=id.](https://www-cdc-gov.translate.goog/museum/timeline/covid19.html?_x_tr_hl=id&_x_tr_pto=tc&_x_tr_sl=en&_x_tr_tl=id)
665 4.	Ndugga N, Hill L, Artiga S, Haldar S. Latest data on COVID-19 vaccinations by
666 race/ethnicity. Kais Family Found. 2021.
667 5.	Nikolovski J, Koldijk M, Weverling GJ, Spertus J, Turakhia M, Saxon L, et al. Factors
668 indicating intention to vaccinate with a COVID-19 vaccine among older US adults. PloS one.
669 2021;16(5):e0251963.
670 6.	Gatwood J, McKnight M, Fiscus M, Hohmeier KC, Chisholm-Burns M. Factors influencing
671 likelihood of COVID-19 vaccination: a survey of Tennessee adults. American Journal of Health-
672 System Pharmacy. 2021;78(10):879-89.
673 7.	Yasmin F, Najeeb H, Moeed A, Naeem U, Asghar MS, Chughtai NU, et al. COVID-19
674 vaccine hesitancy in the United States: a systematic review. Frontiers in public health.
675 2021;9:770985.
676 8.	Christ C. Vaccine Prioritization in Arizona: Arizona Department of Health Services; 2020
677 [cited 2026].	Available	from:	[https://directorsblog.health.azdhs.gov/vaccine-prioritization-in-](https://directorsblog.health.azdhs.gov/vaccine-prioritization-in-arizona/)
678 [arizona/.](https://directorsblog.health.azdhs.gov/vaccine-prioritization-in-arizona/)
679 9.	Current Status of COVID-19 Vaccines Rollout Nationwide: April 2021 Update for the
680 Attorney General Community: National Assocation of Attorneys General; 2021 [cited 2026].
681 Available	from:	[https://www.naag.org/attorney-general-journal/current-status-of-covid-19-](https://www.naag.org/attorney-general-journal/current-status-of-covid-19-vaccines-rollout-nationwide-april-2021-update-for-the-attorney-general-community/)
682 [vaccines-rollout-nationwide-april-2021-update-for-the-attorney-general-community/.](https://www.naag.org/attorney-general-journal/current-status-of-covid-19-vaccines-rollout-nationwide-april-2021-update-for-the-attorney-general-community/)
683 10.	Kriss JL. COVID-19 vaccination coverage, by race and ethnicity—national immunization
684 survey adult COVID module, United States, December 2020–November 2021. MMWR Morbidity
685 and Mortality Weekly Report. 2022;71.
686 11.	Sypher K. Southern u.s. includes some of the nation’s best and worst COVID-19
687 vaccination rates: here’s why 2921. Available from: [https://www.apmresearchlab.org/ten-across-](https://www.apmresearchlab.org/ten-across-covid-vaccine)
688 [covid-vaccine.](https://www.apmresearchlab.org/ten-across-covid-vaccine)
689 12.	Beleche T, Ruhter J, Kolbe A, Marus J, Bush L, Sommers B. COVID-19 vaccine hesitancy:
690 demographic factors, geographic patterns, and changes over time. Published online. 2021;27.
691 13.	Soares P, Rocha JV, Moniz M, Gama A, Laires PA, Pedro AR, et al. Factors associated
692 with COVID-19 vaccine hesitancy. Vaccines. 2021;9(3):300.
693 14.	Rathnayake D, Bannon F, Clarke M, Jayasinghe V, Udayanga V. Descriptive analysis of
694 determinants leading to COVID-19 vaccine hesitancy of healthcare workers in Sri Lanka.
695 Population Medicine. 2025;7(March):1-6.
696 15.	Bambra C, Lynch J, Smith KE. The unequal pandemic: COVID-19 and health inequalities.
697 The Unequal Pandemic: Policy press; 2021.
698 16.	Charura D, Hill AP, Etherson ME. COVID-19 vaccine hesitancy, medical mistrust, and
699 mattering in ethnically diverse communities. Journal of racial and ethnic health disparities.
700 2023;10(3):1518-25.
701 17.	Savoia E, Piltch-Loeb R, Goldberg B, Miller-Idriss C, Hughes B, Montrond A, et al.
702 Predictors of COVID-19 vaccine hesitancy: socio-demographics, co-morbidity, and past
703 experience of racial discrimination. Vaccines. 2021;9(7):767.
704 18.	Williams AM, Clayton HB, Singleton JA. Racial and ethnic disparities in COVID-19
705 vaccination coverage: the contribution of socioeconomic and demographic factors. American
706 journal of preventive medicine. 2022;62(4):473-82.
707 19.	Clarke V, Braun V. Thematic analysis. The journal of positive psychology. 2017;12(3):297-
708 8.
709 20.	Mantina NM, Block Ngaybe M, Johnson K, Velickovic S, Magrath P, Gerald LB, et al.
710 Racial/ethnic disparities in influenza risk perception and vaccination intention among Pima County
711 residents in Arizona. Human vaccines & immunotherapeutics. 2022;18(7):2154506.
712 21.	Alvidrez J, Castille D, Laude-Sharp M, Rosario A, Tabor D. The national institute on
713 minority health and health disparities research framework. American journal of public health.
714 2019;109(S1):S16-S20.
715 22.	Oktay JS. Grounded theory: Oxford University Press; 2012.
716 23.	O’Connor C, Joffe H. Intercoder reliability in qualitative research: Debates and practical
717 guidelines. International journal of qualitative methods. 2020;19:1609406919899220.
718 24.	Zimmerman T, Shiroma K, Fleischmann KR, Xie B, Jia C, Verma N, et al. Misinformation
719 and COVID-19 vaccine hesitancy. Vaccine. 2023;41(1):136-44.
720 25.	Washington HA. Medical apartheid: The dark history of medical experimentation on Black
721 Americans from colonial times to the present: Doubleday Books; 2006.
722 26.	Grier WH, Cobbs PM. Black rage: Wipf and Stock Publishers; 2000.
723 27.	Vázquez E, Juturu P, Burroughs M, McMullin J, Cheney AM. Continuum of trauma: fear
724 and mistrust of institutions in communities of color during the COVID-19 pandemic. Culture,
725 Medicine, and Psychiatry. 2024;48(2):290-309.
726 28.	Strully KW, Harrison TM, Pardo TA, Carleo-Evangelist J. Strategies to address COVID-19
727 vaccine hesitancy and mitigate health disparities in minority populations. Frontiers in Public
728 Health. 2021;9:645268.
729 29.	Casselman-Hontalas A, Adams-Santos D, Watkins-Hayes C. Discourses of distrust: how
730 lack of trust in the US health-care system shaped COVID-19 vaccine hesitancy. RSF: The Russell
731 Sage Foundation Journal of the Social Sciences. 2024;10(4):154-72.
732 30.	Privor-Dumm L, King T. Community-based strategies to engage pastors can help address
733 vaccine hesitancy and health disparities in black communities. Journal of health communication.
734 2020;25(10):827-30.
735 31.	Betsch C, Bach Habersaat K, Deshevoi S, Heinemeier D, Briko N, Kostenko N, et al.
736 Sample study protocol for adapting and translating the 5C scale to assess the psychological
737 antecedents of vaccination. BMJ open. 2020;10(3):e034869.
738 32.	Betsch C, Schmid P, Heinemeier D, Korn L, Holtmann C, Böhm R. Beyond confidence:
739 Development of a measure assessing the 5C psychological antecedents of vaccination. PloS one.
740 2018;13(12):e0208601.
741 33.	Webb Hooper M, Nápoles AM, Pérez-Stable EJ. No populations left behind: vaccine
742 hesitancy and equitable diffusion of effective COVID-19 vaccines. Journal of General Internal
743 Medicine. 2021;36(7):2130-3.

744

[Click here to access/download](https://www2.cloud.editorialmanager.com/pone/download.aspx?id=44841569&guid=6ae9ae4f-85a1-449e-acaa-04127980ffe8&scheme=1)

[**Supporting Information** IRB Approval Letter.pdf](https://www2.cloud.editorialmanager.com/pone/download.aspx?id=44841569&guid=6ae9ae4f-85a1-449e-acaa-04127980ffe8&scheme=1)

[Click here to access/download](https://www2.cloud.editorialmanager.com/pone/download.aspx?id=44841570&guid=adecd000-0ac4-4e84-8c2d-1058a0148029&scheme=1)

# [Supporting Information](https://www2.cloud.editorialmanager.com/pone/download.aspx?id=44841570&guid=adecd000-0ac4-4e84-8c2d-1058a0148029&scheme=1)

## [Appendix K.2 Consent form for focus group.pdf](https://www2.cloud.editorialmanager.com/pone/download.aspx?id=44841570&guid=adecd000-0ac4-4e84-8c2d-1058a0148029&scheme=1)

[Click here to access/download](https://www2.cloud.editorialmanager.com/pone/download.aspx?id=44841571&guid=cf17c2cd-a229-41ef-be4f-3ecba561b787&scheme=1)

# [Supporting Information](https://www2.cloud.editorialmanager.com/pone/download.aspx?id=44841571&guid=cf17c2cd-a229-41ef-be4f-3ecba561b787&scheme=1)

## [Appendix K.2.1 SPA Consent form for Focus Groups Spanish.pdf](https://www2.cloud.editorialmanager.com/pone/download.aspx?id=44841571&guid=cf17c2cd-a229-41ef-be4f-3ecba561b787&scheme=1)

1

#### 1 COVID-19 Vaccine Confidence Among Adults of Pima County using the NIMHD Minority

2 **Health and Health Disparities Research Framework: A Qualitative Analysis**

3

#### 4 Short Title: COVID-19 Vaccine Confidence in Pima County Adults: A Qualitative Study

5 **Using the NIMHD Framework**

6

7 Kim Ellis^1^, MSc, MPH; Pamela Hall^2^, PhD; Latrisha Robinson^3^, MSc; Sara Ruiz^1^ Rn-MSN,
8 MPH; Maiya G Block Ngaybe^1^, MPH, PhD; Christopher Lessenich^1^, MPH; Namoonga M.
9 Mantina, MsPH, MBA, PhD^1^; Carlos M Perez-Velez^4,5^, MD; Beatrice Krauss^5^, PhD; Purnima
10 Madhivanan, MBBS, MPH, PhD ^*^ ^1,7^

11

12 ^1^ Mel & Enid Zuckerman College of Public Health, University of Arizona, Tucson, Arizona, USA
13 ^2^ Department of Psychology, College of Arts and Sciences, Barry University, Miami Shores,
14 Florida, USA
15 ^3^ Department of Psychology, College of Arts, Sciences & Education, Florida International
16 University, Miami, Florida, USA
17 ^4^ Division of Epidemiology, Pima County Health Department, Tucson, Arizona, USA
18 ^5^ Division of Infectious Diseases, College of Medicine, University of Arizona, Tucson, Arizona,
19 USA
20 ^5^ Psychology Department, College of Science, University of Arizona, Tucson, Arizona, USA
21 ^6^ Public Health Research Institute of India, Mysore, India

22

#### 23 Corresponding Author

24 Purnima Madhivanan, MBBS, MPH, PhD
25 Department of Health Promotion Sciences, Mel & Enid Zuckerman College of Public Health
26 University of Arizona,
27 1295 N Martin Avenue, PO Box 245209, Tucson, AZ 85724-5209
28 (520) 621-5730; [pmadhivanan@arizona.edu](mailto:pmadhivanan@arizona.edu)

29

30 **Word count:** 5494
31 **To submit for publication at PLOS ONE**

32

33 **Keywords:** Vaccination Refusal, COVID-19 vaccine, Patient Acceptance of Health Care, Trusted
34 Sources of Information, Health Disparities

#### 35 Abstract

36 **Introduction**
37 Vaccinations have been established as one of the most effective strategies to prevent the
38 spread of infectious diseases. However, vaccine hesitancy has been a growing issue that needs to
39 be monitored and understood to ensure successful promotion. Historic structural, social, and health
40 disparities have been demonstrated to have significant impact on many health outcomes including
41 vaccination uptake. This study applied the National Institute on Minority Health and Health
42 Disparities Research Framework to examine factors influencing COVID-19 vaccination intention
43 during the COVID-19 pandemic among adults in Pima County, Arizona.

#### 44 Methods

45 Between 22 Aug and 05 Dec 2020, 10 focus group discussions stratified by race/ethnicity,
46 gender and language spoken (FGDs) were conducted virtually among 56 adult residents of Pima
47 County, Arizona. After transcription and translation of FGDs conducted in Spanish, transcripts
48 were organized by demographic characteristics and coded in Dedoose software by four
49 independent coders. The NIMHD Framework’s Domains of Influence were used as *a priori* codes
50 in the thematic analysis. All participants completed an informed consent process prior to
51 participation.

#### 52 Results

53 Of the 56 participants who took part in the ten focus groups, 27% self-reported being
54 Hispanic or Latinx, 25% were White/non-Hispanic, 9% were Black, and 4% were Asian. The
55 preferred language was most often reported as English. Unique challenges for each demographic
56 group emerged from this analysis, such as language barriers among Hispanic participants,
57 historical mistreatment concerns among Black participants, and conflicting political messages for
58 White participants. Cultural norms, family influences, and attitudes toward autonomy versus
59 community responsibility further shaped vaccine decisions. Effective communication about
60 vaccine safety, clinical trials, and public health messaging were identified as critical to addressing
61 mistrust and vaccine confidence across diverse groups.

#### 62 Conclusion

63 Major themes in this study that predict vaccine intention include health literacy challenges,
64 misinformation, mistrust in healthcare systems, and the desire to protect others. Understanding the
65 behaviors and decisions behind vaccination acceptance and receipt is important for developing
66 effective vaccine communication strategies. Identifying health disparities driving vaccination
67 hesitancy may help guide future strategies and policies to improve vaccination rates, especially
68 among minority groups.

#### 69 Introduction

70 The World Health Organization (WHO) recognized vaccine hesitancy as one of the top
71 ten global health threats of 2019 since inadequate vaccination rates allow a disease to spread and
72 create new variants [1]~~(WHO, 2019)~~. In recent years, COVID-19 has been listed as the third
73 leading cause of death in the United States (US), resulting in more than 6.5 million
74 hospitalizations and about 1.2 million deaths [2]~~(CDC, 2025)~~.
75 Despite high morbidity and mortality rates, acceptance of COVID-19 vaccine remained
76 low after its initial release. The COVID-19 vaccination program was launched on December 14^th^,
77 2020, in the US [3]~~(CDC, 2024)~~. Ndugga et al*~~et al (~~*~~2021)~~ found that 48% of Caucasians had
78 received the vaccine by July 2021, while only 38% of Blacks and 41% of Hispanics had received
79 the vaccine [4]. A study by Nikolovski et al ~~(2021)~~ found that 91% of adults aged 65 and older

**Formatted:** Font: Not Italic

80 were willing to get vaccinated against COVID-19 [5], and Gatwood et al ~~(2021)~~ found an

**Formatted:** Font: Not Italic

81 acceptance rate of 45.9% in adults aged 18-64 [6]. A review found that COVID-19 vaccination
82 rates in the US among various populations ranged from a low of 12% to a high of 91%
83 [7]~~(Yasmin et al., 2021)~~. The same paper also mentioned that Arizona had recorded one of the
84 highest rates (76%) of COVID-19 vaccine acceptance in the USA [7].~~(Yasmin et al, 2021).~~
85 The Arizona Department of Health Services (ADHS) noted that the vaccine was available
86 to all adults in Arizona (AZ) ~~by April 11~~^th^~~, 2021, and about 40% had received at least one dose~~in
87 December, 2020 [8]~~(2021)~~. ~~To date, 80% of the population has been vaccinated with at least one~~
88 ~~dose of the COVID-19 vaccine in the state of AZ (CDC, 2024). This is close to the national~~
89 ~~percentage of 82.3% (MMWR, 2024).~~ By April ~~11~~^th^, 2021, about 40~~5~~% had received at least one
90 dose [8], higher than the national average rate of 29.5% at the time [9]., Disparities among

**Formatted:** Font: (Default) Times New Roman

91 minority races and ethnicities in COVID-19 vaccination coverage persisted nationwide in

**Formatted:** Font: (Default) Times New Roman

**Formatted:** Font: (Default) Times New Roman

92 vaccine rates [10] as well as in Arizona [11].~~and by July 10~~^th^~~, 2022, 79% were vaccinated with~~
93 ~~one dose (ADHS, 2022). According to the CDC, by April 18~~^th^~~, 2021, 30% of adults in Pima~~
94 ~~County were fully vaccinated against COVID-19, and this increased to 67% just over one year~~
95 ~~after the launch of the COVID-19 vaccination program (CDC, 2022). More than 14 million~~
96 ~~COVID-19 vaccine doses had been given in Pima County with 77% having received at least one~~
97 ~~dose (ADHS, 2023). Whites comprised 44% of one dose received, while only 21% were~~
98 ~~Hispanic or Latino. A significant disparity in vaccine rates was observed in Pima County with~~
99 ~~only 3% of Black residents having received a single dose (ADHS, 2023).~~
100 Multiple factors have been found to be associated with an individual’s decision to accept,
101 refuse, or hesitate to get vaccinated, including religion, cultural background, socioeconomic status,
102 political beliefs, the nature of vaccine communication, media consumption, past experiences with
103 vaccination, sociodemographic characteristics and perceived benefits and risks [12-14]~~(Beleche,~~
104 ~~2021; Soares et al., 2021; Ratnayake et al., 2024)~~. Communities of Color (CoC) have been shown
105 to face disproportionate rates of infection and death due to COVID-19 [15]~~(Bambra et al., 2010)~~.
106 Vaccine hesitancy rates have been shown to be the highest among minority groups in the US [6,
107 16, 17]. ~~(Charura et al., 2022; Gatwood et al., 2021; Savoia et al., 2021; Reese et al., 2023).~~ For
108 example, Ratnayake et al., ~~(2024)~~ found that Blacks had double the likelihood of vaccine hesitancy
109 compared to Whites [14]. Similar results were reported by Savoia et al~~.~~, ~~(2021) and Reese et al.,~~
110 ~~(2023)~~ with rates of vaccine hesitancy among Blacks being 70% in comparison to 22% for Whites
111 [17]. A study by Williams et al., ~~(2022)~~ revealed that vaccination coverage is lower among all
112 racial and ethnic minority groups except Asians when compared to Whites [18]. It has also been
113 shown that Hispanics and Blacks had the highest rate of participants that were undecided about
114 vaccination or less likely to be fully vaccinated [4, 16, 18]. ~~(Williams et al., 2022; Charura et al.,~~
115 ~~2022; Ndugga et al., 2021).~~ While Savoia et al~~. (2021)~~ found similar results, their results differed
116 in that they found that Hispanics were slightly less likely to be vaccine hesitant when compared to
117 other races [17]. Though White communities generally fare better across many health measures
118 compared to other racial and ethnic groups, disparities still exist in White communities. Several
119 social determinants of health that impact White communities include socioeconomic status,
120 neighborhood, and built environment [4].~~(Ndugga, et al. 2024; Lasser, et al 2024).~~
121 In this paper, we analyzed COVID-19 vaccine decision-making at the time when the
122 vaccine was in the initial stages of its rollout. Relying on a health disparities research framework,
123 this qualitative study examined the decisions that impacted vaccine receipt, acceptance, and
124 hesitancy in CoC and White communities in Pima County, AZ.

#### 125 Methods

126 *Participants*
127 The study took place in Pima County, Arizona during the COVID-19 pandemic’s onset
128 with recruitment done between 22 Aug and 05 December, 2020, prior to vaccine rollout in the
129 United States. ~~.~~ We conducted 10 focus group discussions (FGDs) with a total of 56 participants.
130 Approximately 27 additional participants who had been recruited for the study failed to show up
131 for their scheduled FGD for unknown reasons. We used purposive sampling methods for our study
132 to ensure representation across race/ethnicities (White, Latinx and Black), Language (English and
133 Spanish), and gender (Male and Female) from the networks of the study team. All participants
134 provided informed consent before participating in any research activities. The Institutional Review
135 Board at the University of Arizona reviewed and approved the study (Protocol ID: 2007796226).
136 We recruited participants to the study via social media, or email using ~~IRB~~ ethical review board-
137 approved recruitment scripts and digitally shared flyers. Participants often belonged to the same
138 or similar social groups, such as an African American wellness group, or a group of college
139 students familiar with a member of the research team, or a Hispanic activist group. Study team
140 members would contact potential participants to invite them to participate in the study. Participants
141 were included if they were above 18 years of age and were living in Pima County at the time of
142 the study. Upon recruitment, participants were introduced to the research study and its purpose,
143 fore-shadowing informed consent. ~~All participants provided informed consent before participating~~
144 ~~in any research activities. The Institutional Review Board at the University of Arizona reviewed~~
145 ~~and approved the study (Protocol ID: 2007796226).~~
146 *Interview Guide Development*
147 The semi-structured focus group discussion guide and prompts were developed iteratively
148 by the team by using established qualitative methods [19]. ~~(Braune & Clarke, 2017).~~ The team
149 conducted a pilot FGD internally before using the guide with participants; no repeat interviews
150 were conducted.
151 *Interview Setting*
152 FGDs were held on an online virtual Zoom platform and lasted between 60 to 90 minutes.
153 All participants were initially let into the Zoom room individually, while others were in the waiting
154 room, to explain that they could change their name displayed on the Zoom call and hide their faces
155 if they desired anonymity on the call. All participants completed a verbal informed consent process
156 prior to the FGD. All sessions were audio and video recorded. A notetaker from the research team
157 attended each session to take notes and provide support for the facilitator during each focus group.
158 *Data Management*
159 Debriefing sessions were held at the end of each FGD to discuss ~~data~~ thematic saturation
160 and notes for improvement in future sessions. Transcripts were not returned to participants for
161 comment or correction. The automatic transcription feature of Zoom was used to generate initial
162 transcripts for those held in English and then reviewed while listening to the audio recording by a
163 research team member. Students who were studying Spanish translation were hired to transcribe
164 and translate FGD sessions held in Spanish. For further details about the participants and the
165 recruitment process and the analysis of data collected about seasonal influenza-related outcomes,
166 refer to the article by Mantina et al [20]~~. (2022)~~.
167 *Reflexivity Statement*
168 The interviewers were a male Hispanic medical care provider practicing at the Pima County
169 Health Department (CPV), a male Black PhD student (FM), a female professor of Indian origin,
170 who was also the PI of the project with a PhD and medical degree (PM), and a female mixed race
171 MPH student who was acting as study coordinator (MBN). All had some experience in qualitative
172 research and received some additional training from the study team to ensure consistency. BK
173 consulted on qualitative methods, interview design, and analysis. PM and CPV were the members
174 of the team who conceptualized the project and determined that it was important to conduct this
175 research to explore the evolving issues of a possible COVID-19 vaccine and possible factors that
176 could be associated with future vaccine acceptance to inform a promotion strategy for the Pima
177 County Health Department.
178 The qualitative analysis team included the lead MPH student (KE), two PhD students
179 (MBN, LR), and a professor (PH). One of the PhD students (MBN) had been an interviewer, had
180 previous experience in qualitative research, and served as the research project coordinator. One of
181 the PhD students (LR) had limited experience in qualitative research and was studying Applied
182 Social and Cultural Psychology. The professor of psychology (PH) who helped with the data
183 analysis is a PhD holder and experienced researcher in social psychology and minority mental and
184 public health research. All analysts identified as female, two as black (LR, PH), one as mixed race
185 (MBN), and one as white (KE). Researchers who provided feedback and guidance on the
186 qualitative research included one white male MPH student with more than three years of public
187 health research experience (CL), a black female PhD student with at least 3 years of experience in
188 qualitative research (NM), and a white female researcher with decades of experience in
189 community-based qualitative research (BK).
190 *Theoretical Framework*
191 The National Institute on Minority Health and Health Disparities (NIMHD) framework
192 accounts for social factors such as education and income[21]. In this study, we utilized this
193 framework since it focused on Black, White, and Latinx communities. The NIMHD research
194 framework is broadly used in the literature to explain the root causes of health disparities and social
195 determinants of health in CoCs [21]~~(Alvidrez et al., 2019)~~. Building on the ideas of the socio-
196 ecological model’s levels and concepts from a similar health disparities research framework from
197 the National Institute of Aging (NIA), the NIMHD research framework is a “tool for
198 conceptualizing and depicting the wide array of determinants that promote or worsen minority
199 health or cause, sustain, or reduce health disparities.” [21]. The framework was intended to help
200 guide health disparities research by demonstrating different factors at different levels of society
201 that may contribute to the risk and resilience factors related to health equity and inequities. The
202 framework has two axes, with domains of health (biological, behavioral, physical and built
203 environment, socio cultural environment, and healthcare system) depicted on the x-axis and levels
204 of influence on health (individual, interpersonal, community, and societal) mapped onto the y-axis.
205 This visual representation of the NIMHD framework also serves as a guide for research on health
206 disparities, though it has yet to be used widely. It will be referred to as “the framework” henceforth.
207 There is a more detailed description of the framework in **Appendix 1**.

208

209 *Data Analysis*
210 The qualitative analysis team began by familiarizing themselves with the FGD transcript
211 data. A framework content analysis was using primarily a deductive approach when identifying
212 themes, mapping them onto the ~~NIMHD Research Framework’s Domains of Influence~~framework
213 as predetermined codes, which were applied in Dedoose. The team created a codebook of the
214 Domains of Influence with definitions for each domain, including biological, social, psychological,
215 etc. They collaboratively created matrices with definitions and examples to easily determine which
216 type of data would be coded under each Domain of Influence. The Levels of Influence were
217 included within each definition of the Domain codes. Emerging themes which may not have easily
218 fit clearly under one Domain were additionally identified inductively and included as their own
219 separate codes. When developing the codebook, the team discussed the overlap in the framework
220 and determined through consensus which information was included within each code.
221 The transcripts were each coded by mapping the content from each transcript from each
222 group onto the framework matrix by one person on the team. ~~and then~~Subsequently, a second coder
223 from the team reviewed the ~~codes~~ matrices for each group to verify for accuracy, taking notes and
224 making tracked changes to the codes as necessary, reviewing the transcript as they reviewed the
225 coded data. The team held regular discussions to discuss the codes that each team member applied
226 to the transcript and reso ~~[22]~~lved conflicts as a group through iterative discussion and consensus.
227 During these meetings, suggested changes to the way the data were organized or “coded” onto the
228 NIMHD framework matrices were discussed. We did not calculate inter coder reliability because
229 conflicts were consistently resolved as a group and the dataset was smaller [22, 23]. In order to
230 ensure sufficient rigor, many discussions were held to ensure data were coded accurately,
231 iteratively until all team members were satisfied. Each team member additionally took notes on
232 the transcripts to further identify meanings, patterns, and comparisons among the data. The last
233 step of the analysis involved in-depth discussion of each code applied in the transcript, assessing
234 existing patterns and determining relevance to sociocultural factors, personal experience, medical
235 history, and other relevant factors. ~~Each team member took notes on the transcripts to further~~
236 ~~identify meanings, patterns, and comparisons among the data.~~
237 To display the examples of the themes that emerged, tables were created for each of the
238 focus groups. While analyzing the data and developing themes, one additional code was added
239 inductively to capture politics. The team created matrices and wrote summaries of findings for
240 each racial/ethnic category of focus group and used these to create the results presented in this
241 manuscript. See **Supplemental File 1** for the initial summaries made from each racial/ethnic
242 category of focus groups. The codebook created based on the framework and its domains and
243 corresponding definitions are included in **Appendix 1.** The Consolidated criteria for reporting
244 qualitative research (COREQ) was used to inform how information was reported in this
245 manuscript.

246

#### 247 Results

248 Of the 56 participants who took part in the ten focus groups, 34% were female. While 27%
249 self-reported being Hispanic or Latinx, 25% were White, 9% were Black, and 4% were Asian,
250 noting that some participants may have fallen under multiple categories. The preferred language
251 was most frequently reported to be English (24, 43%) followed by Spanish (11, 20%).
252 Demographic information was not reported for almost half of the participants (21, 38%). Although
253 participants were grouped into focus groups based on their social groups, since they did not
254 disclose their demographic information consistently, we will not report it at an individual level. It
255 is important to note that some groups had small representation, limiting the interpretation of
256 subgroup comparisons. The themes identified and mapped onto the NIMHD framework are
257 reported in Table 1.

258

#### 259 Table 1: NIMHD Framework-Aligned Themes on Vaccine Perceptions and Calculated

260 **Risks by Racial/Ethnic Group**

261

| **Domain** | **Black Focus Groups** | **Hispanic/Latino Focus Groups** | **White Focus Groups** |
| --- | --- | --- | --- |
| **Biological Domain** | Family vulnerabilities (e.g., heart conditions) | Fear of infecting elderly parents | Individual immunity, calculated risk |
| **Behavioral Domain** | Concerns about long-term effects, protecting others | Self-isolation and strict precautions | Social responsibility, protecting others |
| **Physical/Built Environment** | Environmental hygiene (e.g., mask, gloves, cleaning car) | Room isolation, household distancing | Institutional concerns (school outbreaks) |
| **Sociocultural Environment** | Historical mistrust (Tuskegee) | Community-based trust and privacy | Trust in close circles, confusion over messaging |
| **Healthcare System** | Distrust due to testing bias & lack of representation | Conflicting medical advice & uncertainty | Concerns about rushed trials, desire for clear information |
| **Politics (Inductive Theme)** | Mistrust of CDC, trust in local health officials, distrust of political media | Belief that COVID is politically motivated | Distrust of political media, concern about rushed rollout |

262

263 *Biological Domain*
264 Participants across racial groups highlighted biological factors affecting their vaccine-
265 related decisions. Biological vulnerabilities, such as chronic conditions, significantly shaped
266 attitudes and behaviors towards vaccination. For instance, a White male participant was concerned
267 about the possibility of being infected while in public areas,
268 *“I think my risks of getting it are high. And I think I take precaution, and I am pretty*
269 *proactive on taking care of myself. But even with me taking those steps and working, for I*
270 *have the privilege of working from home, I think I still-- anytime you're in public, I think*
271 *you're putting yourself at risk. So, I think there's a high probability of either encountering*
272 *someone who has COVID-19 or is either connected. So, I think- my framework that I use*
273 *is anytime you're out in public or outside you're at risk of getting it.” (p25)*
274 Black participants expressed awareness of familial vulnerabilities, illustrating heightened caution
275 due to chronic illnesses among family members:
276 *“So, I just try to protect my grandchildren and my daughter, who has a bad heart. I tried*
277 *to pick those, you know, things like that.” (p29)*
278 A Hispanic participant similarly emphasized maintaining health and protecting loved ones:
279 *“Immediately have hand sanitizer, or wash [my daughter’s] hands, and don’t touch*
280 *anything, don’t touch your face […] You don’t pay attention to what you are going to do*
281 *because you’re watching them. Because there is fear-- my fear is that she infects my parents*
282 *because they are vulnerable.” (p36)*
283 Participants collectively recognized that individual health directly influenced decisions to
284 vaccinate for broader community protection, with a Black male participant noting communal
285 responsibility said:
286 *“I feel like it’s just my responsibility to the rest of the community” (p53).*
287 Black and Hispanic participants prominently emphasized familial and community biological
288 vulnerabilities, contrasting with White participants, who focused more on individual health and
289 robust immunity.

290

291 *Behavioral Domain*
292 Behavioral factors influencing preventive practices and vaccine hesitancy were articulated
293 across groups. Hispanic participants frequently described cautious preventive measures at home
294 and working on proactive strategies*.* One Hispanic participant added,
295 *“What I would do is isolate myself, you understand me, from my family as well to not get*
296 *other people sick and that’s what we are doing right now” (p50).*
297 A Black participant expressed significant concerns regarding vaccine uncertainties, stating,
298 *“Long term health effects…they can't tell us what it does to us five years in the future,*
299 *until it starts popping up five years in the future. So that's the biggest worry” (p53).*
300 White participants framed their decisions around familial responsibilities, stating,
301 “*Yeah, protect other people...my motivator is to protect them…it's my social responsibility*
302 *not to be a vector, if possible” (p9)*.
303 Across groups, community responsibilities emerged as a common theme. Societal
304 mandates played a significant role in shaping behaviors, particularly in cases where vaccination
305 was required for immigration or employment purposes. Behavioral responses differed across racial
306 and ethnic groups in our study: Black and Hispanic participants tended to express more cautious
307 approaches influenced by uncertainties around the vaccine, while White participants more
308 frequently emphasized responsibility to protect others in their social and family circles:

| 309 | *“But that's like how I think about it like I don't want to get my parents sick. You know my* |
| --- | --- |
| 310 | *grandmas are both 87 they would die” (p18).* |
| 311 |  |
| 312 | *Physical/Built Environment* |
| 313 | Physical and built environmental contexts influenced health behaviors among participants. |
| 314 | A Black participant particularly emphasized rigorous environmental hygiene practices as critical |
| 315 | preventive measures: |
| 316 | *“I don't go out to the malls and family gatherings and stuff where there's a lot of people,* |
| 317 | *so therefore I go out, mainly when there's an essential reason and I try to do all those* |
| 318 | *things. So, to distance myself, I use a mask and gloves if necessary. Then I pull them off* |
| 319 | *and dispose of those gloves there at the store or where I am.I don't want any germs in my* |
| 320 | *car. And when I pick up my groceries, I call them and pick them up from the store. I have* |
| 321 | *to put it in place into my trunk because again I want nothing near me.” (p32)* |
| 322 | Similarly, a Hispanic male participant detailed significant home isolation strategies to prevent viral |
| 323 | transmission within families, describing actions such as |
| 324 | *“She [my daughter] calls us from her work that she did not want to have direct contact* |
| 325 | *with us, because a person came out positive, then what we did was that we started fixing* |
| 326 | *the room and leaving the house to go live with another daughter in the same place.” (p55)* |
| 327 | A few White male participants briefly mentioned concerns about professional environments like |
| 328 | schools, acknowledging risks within institutional settings, such as, |
| 329 | *" New cases in Tucson are being driven by the university student population, particularly* |
| 330 | *the large student housing complexes. And so, I have concern for, for my colleagues who* |
| 331 | *are teaching and interacting with those students.” (p24)*. |

332 This domain revealed focused personal and familial environmental adjustments
333 predominantly among Black and Hispanic participants. Black and Hispanic participants provided
334 more detailed descriptions of proactive ecological strategies than White participants, who mainly
335 acknowledged broader institutional environmental risks. Participants were more concerned about
336 bringing the virus into their physical/built environments.

337

338 *Sociocultural Environment*
339 Sociocultural factors profoundly influenced participant perspectives, including historical
340 mistrust, cultural norms, and social networks. Black participants notably referenced historical
341 injustices, exemplified by the Tuskegee Syphilis Study. One participant emotionally stated,
342 *“Getting ready to get a little deep for a moment. Oh, the Tuskegee Syphilis Study done on*
343 *African American. I want to say with all my goodness, maybe the 20s, 30s, 40s. I don't*
344 *know the exact date, but that sticks in my mind, of giving People of color placebo … Oh, it*
345 *makes me cry sometimes because it's so sad… I am hesitant to go to the doctors. There’s*
346 *been [a] long line and a long history of African American people, whether we are older or*
347 *younger, or female or male not being treated right in the health care system.” (p43)*
348 Some Hispanic participants placed significant trust in local communities, describing cultural
349 privacy and preventive practices. As one participant said,
350 *“I think that in the Hispanic environment, or so to speak, there is a little more privacy in*
351 *terms of people taking into account... Well, I am talking about the circle closest to me, and*
352 *they take into account that we must follow the rules precisely so as not to become infected.*
353 *For example, there are not so many parties, I don't know if they go to so many bars and all*
354 *that.” (p55).*
355 A White male participant emphasized interpersonal trust within immediate social groups as
356 pivotal:
357 *“I think … from my perspective, I think we've kept our circle pretty tight. And I think we've*
358 *kind of been- especially during these times- we've kept it... Well, I think, trust, trust, trust*
359 *in each other. And I think we've been hanging out a lot with like-minded people that kind*
360 *of see this in the same light. So, trust really has played a big part in who we spend time*
361 *with, who we hang out [with]. So, I think that has been a huge part in our, in our circle.”*
362 *(p25)*
363 Societal influences also emerged clearly, with participants across groups expressing deep
364 frustration over politicized and inconsistent health information: *“[T]hey confuse people more like*
365 *that, and they scare them more because they are the experts; they should be constant” (p37).* Some
366 Black participants ~~distinctly~~ highlighted systemic discrimination and historical mistrust., Hispanic
367 participants often demonstrated strong community-centered trust, and White participants often
368 focused on confusion due to societal misinformation.
369 These findings illustrate how vaccine decision-making was shaped not only by individual

**Formatted:** Font: (Default) Times New Roman, 12 pt

370 beliefs but also by interpersonal trust, community norms, and broader societal narratives. Within
371 the NIMHD framework, sociocultural influences operated across levels: family and close social
372 networks shaped perceived responsibility, community histories shaped mistrust, and politicized
373 national discourse shaped confidence in public health institutions.

374

375 *Health Care System*
376 Participants expressed widespread skepticism and mistrust regarding healthcare systems,
377 highlighting communication, accessibility, representation, and care quality concerns. Some
378 Hispanic participants notably voiced confusion due to inconsistent messaging from healthcare
379 authorities:
380 *“The ones who did the exam said she should stay seven more days after the test. The*
381 *pediatrician tells me that she should stay ten more days. Who is right? They told me, ‘Well,*
382 *in reality we don’t know.’ So, if you are trusting someone and they tell you ‘well we don’t*
383 *know because it’s new.’ Who are you going to trust?” (p36)*
384 Some Black participants articulated specific apprehensions regarding different cultural
385 representations in vaccine trials*.* A participant expressed broader mistrust:
386 *“Medications are done on white people and maybe white men mostly. Yeah, just save it.*
387 *And so, we are in the minority when they are testing out these new medications, and then*
388 *they are being used on you. Used in a negative way. A lot of work is done in Caribbean*
389 *and African countries, and people are not well informed.” (p45)*
390 A White participant emphasized concerns about rapid vaccine development:
391 *“One of my concerns is because it has a profit motive, but also because it's a race right*
392 *now. Not just cutting corners, but just efficacy may not be measured as stringent as it*
393 *should be. And so, the first-generation vaccines may or may not be effective, but they also*
394 *probably [have] been run through as much rigorous testing in order to have a time frame*
395 *and that worries me.” (p8)*
396 Additionally, another White participant highlighted the need to understand vaccine implications
397 clearly:
398 *“ I'd want to know the effects of it. Is it you know, something, you get the vaccine, but you*
399 *still have, you know, some odd percentage or chance of being exposed to it and getting it?*
400 *It may just be mild or? I just want to understand If I did get the vaccine, what does that*
401 *mean as far as risk of exposure, and if I got sick, what that means.” (p20)*
402 While mistrust towards healthcare systems was common across all groups, Black participants
403 particularly emphasized representation issues and systemic barriers, Hispanic participants focused
404 on confusion from inconsistent messaging, and White participants emphasized trust in
405 interpersonal healthcare relationships.

406

407 Inductive Theme: Politics
408 In our analysis, we identified a significant theme not captured in the NIMHD Research
409 Framework: the role of the political environment in influencing COVID-19 vaccine hesitancy.
410 Participants across racial and ethnic groups expressed concerns about political interference
411 potentially compromising the integrity of vaccine development. Some of the Hispanic/Latino
412 participants felt that COVID-19 was ~~a~~ fake, and being used by the government to cover something
413 up, and expressed a fear of what was being put into the vaccine. Participants from this group
414 tended to not trust President Trump and reported that if the vaccine were to come out before the
415 election, they would not trust the COVID-19 vaccine. Many participants also felt that scientists
416 were disagreeing with each other, and that countries were competing instead of trying to develop
417 a vaccine that would collectively help humanity. Similarly, a Hispanic female voiced frustration
418 over the politicization of vaccine efforts, commenting,
419 *“I see more political taint in the countries, more than the desire… for curing this sickness”*
420 *(p4).*
421 Some of the Black participants felt that those in power were able to influence what is being
422 said in the media. Participants in this group mentioned that the COVID-19 vaccine was being too
423 politicized. Distrust in federal health institutions was common. However, many did trust their local
424 government officials. A~~n~~ Black mixed gender group participant noted,
425 *“I would not trust the CDC, the NIH, or any public health [officials] that he has control*
426 *over… There's just too much politics and political pressure. But at the local level, like Dr.*
427 *Garcia…I trust him…”* (p30).
428 Some of the White participants felt that more people would be willing to receive the
429 COVID-19 vaccine if information was given via a non-political platform. They note that CNN,
430 Fox News and MSNBC are very political. In line with the Hispanic/Latino and Black participants,
431 White participants also felt that the vaccine was being rushed, and that President Trump was using
432 the vaccine to grow his popularity.
433 *“I think it is completely politicized and that would be the only reason that it would be*
434 *available is because Trump has gone through all the chains of commands to make that*
435 *happen … in order to grow his base. I'm completely pro vaccine, but I really don't think I*
436 *could get behind that.”* (p18)
437 For some, political views extended to personal beliefs, as shared by a White female participant:
438 *“My father… bought into the idea that COVID was a hoax… mainly because he didn’t*
439 *know anyone who had gotten it”* (p10).
440 This theme highlights the importance of recognizing how political dynamics can shape public trust
441 in health initiatives.
442 While all groups expressed concern about rushed vaccine development, the basis of

**Formatted:** Font: (Default) Times New Roman, 12 pt

443 concern differed. Black participants more often linked vaccine concerns to historical mistreatment
444 and underrepresentation in clinical trials; Hispanic participants emphasized uncertainty, family
445 protection, and inconsistent medical guidance; and White participants more often emphasized

**Formatted:** Font: (Default) Times New Roman, 12 pt

**Formatted:** Indent: First line: 0.5"

446 political interference, individual risk calculation, and the need for clear evidence. These
447 differences suggest that similar expressions of hesitancy may arise from distinct social and
448 structural contexts.
449 Across domains, vaccine intention was shaped by the interaction of perceived biological
450 vulnerability, behavioral risk management, sociocultural trust, healthcare system mistrust, and
451 political context. Participants did not describe vaccine decisions as isolated individual choices;
452 rather, they framed decisions as negotiated through family responsibility, prior experiences with
453 healthcare institutions, perceived credibility of messengers, and uncertainty about the speed and
454 transparency of vaccine development. These findings suggest that vaccine hesitancy in this sample
455 was best understood as a multilevel process rather than a single attitude or knowledge deficit.

456

#### 457 Discussion

458 By applying the National Institute on Minority Health and Health Disparities Research
459 Framework in this study, we were able to highlight many factors that influenced adults in Pima
460 County, Arizona to choose to vaccinate or not to vaccinate against COVID-19. There were many
461 common themes among all FGDs, such as participants choosing to take the flu vaccine due to
462 COVID-19 when they might otherwise have not received the flu vaccine. Another common theme
463 shared across the FGDs was wanting to protect others, especially those close to them. A fear that
464 the COVID-19 vaccine was being developed too fast was also mentioned across all FGDs.
465 Health literacy and misinformation were major concerns mentioned across groups, as
466 participants voiced doubts about the accuracy of vaccine information and were skeptical about the
467 vaccine’s effectiveness and safety due to conflicting messages. Concern about safety and side
468 effects has also been mentioned in the literature [24]~~(Zimmerman et al. 2023)~~. Historical and
469 sociopolitical mistrust were also common themes, especially concerning the government’s
470 handling of the pandemic. Mistrust in healthcare systems and public health authorities also
471 surfaced as a significant theme. Washington ~~(2006)~~ suggests that many in the Black community
472 and possibly other CoC have a fear of the “white coat” or what is called iatrophobia[25].
473 Iatrophobia is associated with historical events that create what Grier and Cobbs ~~(1968)~~ long ago
474 described as a type of cultural paranoia [26]. Vazquez et al. ~~(2022)~~ found that historical, cultural,
475 and social trauma induce fear and mistrust in public health and medical institutions, influencing
476 COVID-19 testing and vaccination decisions among Latinx, Indigenous Americans, and Black
477 Americans [27]. Lastly, attitudes toward behavioral protective practices, such as masking and
478 social distancing, also varied significantly across groups with Hispanic participants, especially
479 women, ~~being~~ appearing to be more inclined to view vaccination as part of a comprehensive
480 approach necessary for their family’s safety.
481 While there were some similarities across all the FGDs, we found a few decisions
482 surrounding vaccination that were specific to certain groups. It appeared that White participants
483 ~~mentioned~~ noted the importance of effective messaging. They ~~were~~ also appeared more likely to
484 discuss nurturing one’s own immunity. In contrast, Black and Hispanics ~~were~~ appeared more
485 concerned about protecting friends and family. Black participants ~~echoed~~ at times demonstrated
486 a concern about clinical trials, in particular mentioning worries about if they were ethical and
487 inclusive. They also were the only group to mention~~ed~~ the Tuskegee Project as their reason to not
488 trust the medical community, similar to other studies from the literature[24, 28]~~(Strully et al.,~~
489 ~~2021; Zimmerman et al. 2023)~~. A qualitative study by Casslelman-Hontalas et al~~. (2024)~~ found
490 several participants referring to the Tuskegee syphilis study and felt that COVID-19 was being
491 experimented on Blacks [29]. This demonstrates a similar need for better communication from
492 researchers to the public regarding how these trials are being conducted. This emphasizes the
493 importance of communicating everything from how a vaccine works to the extensive research and
494 clinical trials involved in making a new vaccine.
495 As was mentioned in our study by Black participants, faith and religion ~~were~~ appeared
496 important to them in this context. One way to build trust in the Black community and increase
497 vaccination rates is to involve those they trust such as faith leaders [30]~~(Privor-Dumm & King,~~
498 ~~2020)~~. ~~They~~ Participants in our study recommended implementing a faith-based approach, and the
499 first step was to listen and get to know the community [30]~~(Privor-Dumm & King, 2020)~~. Many
500 Black communities have been shown to trust their pastors in the community; therefore, these
501 pastors may be effective messengers for vaccine-related information [30].~~(Privor-Dumm & King,~~
502 ~~2020).~~
503 Our findings align with established vaccine hesitancy frameworks, including the “3Cs”

**Formatted:** Font: (Default) Times New Roman, 12 pt

504 model of confidence, complacency, and convenience and the expanded “5Cs” model, which
505 includes confidence, complacency, constraints, calculation, and collective responsibility [31, 32].

**Formatted:** Font: (Default) Times New Roman, 12 pt

506 Participants’ concerns about rushed vaccine development, inconsistent messaging, and political
507 interference reflect challenges related to confidence. Their questions about personal risk,
508 immunity, and vaccine safety reflect calculation, while repeated references to protecting family
509 members and community members reflect collective responsibility. However, our findings also
510 suggest that these constructs are experienced through racialized and sociopolitical contexts,
511 particularly among participants who linked vaccine concerns to historical mistreatment,
512 underrepresentation in clinical trials, and mistrust of federal institutions.
513 Importantly, participants’ concerns should not be interpreted solely as misinformation or

**Formatted:** Font: (Default) Times New Roman, 12 pt

514 lack of health literacy. Rather, many concerns reflected reasonable responses to rapidly changing
515 guidance, limited transparency about vaccine development, prior experiences of discrimination,
516 and broader political mistrust. This distinction is important because interventions focused only on
517 correcting misinformation may fail if they do not also address trust, representation, and
518 institutional accountability.
519 Taken together, these findings suggest that vaccine communication strategies should be

**Formatted:** Font: (Default) Times New Roman, 12 pt

520 tailored not only by demographic group but also by the underlying source of concern. Messages
521 addressing safety and trial transparency may be particularly important when concerns are rooted
522 in mistrust of research. Language-concordant, family-centered communication may be especially
523 useful when concerns involve protecting elders and navigating conflicting guidance. Nonpartisan,
524 locally grounded messaging may be critical when hesitancy is shaped by political distrust. Across
525 groups, trusted messengers, transparency about uncertainty, and acknowledgment of historical and
526 contemporary inequities may be more effective than information-only approaches.
527 A strength of this study is the use of a nationally recognized framework on health
528 disparities to determine factors affecting vaccine attitudes among a purposively sampled group of
529 participants. Additionally, this is the first article to use the NIMHD framework in the field of
530 vaccine hesitancy to date to our knowledge. This sample provided diverse perspectives from
531 different communities within Pima County. The information is very locally relevant, making the
532 findings internally valid, especially during this unique period during the COVID-19 pandemic.
533 The findings could help to inform future health promotion campaigns for novel vaccines,
534 especially during a pandemic. Additionally, it is important to emphasize the value of this study
535 applying the NIMHD Minority Health and Health Disparities Research Framework to pre-rollout
536 vaccine confidence perceptions in a diverse county-level context, showing how concerns about
537 trust, health literacy, historical marginalization, access, and information environments operate
538 across multiple levels of influence.
539 Nevertheless, we recognize that the study has its limitations. It is important to note that the
540 findings are from data collected prior to vaccine rollout in the United States, and therefore reflect
541 anticipated perceptions, concerns, and stated intentions under uncertainty rather than actual
542 vaccine uptake behavior. The data were not collected with the NIMHD research framework in
543 mind, which may mean that some of the information related to the framework may have been
544 missing not due to the participants not mentioning it, but rather due to the data collection tool and
545 facilitator not being oriented towards the framework and its domains. Additionally, there was some
546 possibility of misinterpretation of the data, since the analysts were mostly not involved with the
547 data collection, member checking was not performed due to the delay of the analysis post data
548 collection, and many were not from Pima County as well. We acknowledge that formal back-
549 translation was not conducted for Spanish focus groups, which may have affected nuance in
550 interpretation. Zoom and technological issues additionally made it challenging for some
551 participants to actively and comfortably engage in discussion which may have made it more
552 challenging to have free and open dialogue or share accurate and complete information. The format
553 may also have introduced digital divide bias, which could have caused a selection bias. This
554 discomfort with the new technology, changing the format of the discussions from a focus group
555 discussion to more of a group interview format, unintentionally, and also additionally making it
556 more challenging to collect socio demographic data leading to some gaps in the demographic data
557 collected. We also had a limited sample size, so while we made comparison between racial ethnic
558 groups and participants, these participants do not represent everyone with the same race or
559 ethnicity and so it is important to note that findings may not be generalizable. No member checking
560 was conducted in this study which limits its validity, but this was not feasible since this was a
561 secondary analysis conducted on the data years after the data were collected so the participants
562 themselves would be removed from their thoughts from this specific time in their lives and
563 therefore it would not have made sense to conduct. One final limitation of this study is that the
564 use of a disparities framework may have made the analysis biased towards trying to find disparities
565 rather than recognizing similarities too.
566 Scientists at the National Institute on Minority Health and Health Disparities are devoted
567 to addressing and conducting research on health disparities and inequities. [33]~~(Webb Hooper et~~
568 ~~al, 2021)~~. To help bridge the gap between health disparities and to improve vaccination uptake
569 among minority populations, it will be important to have access to data with accurate reporting on
570 vaccination status. Gaps in vaccine uptake will persist until we work towards addressing the health
571 disparities and the drivers behind them. Future studies in Pima County may benefit from a follow-
572 up study to determine how attitudes may have changed regarding these vaccines post-pandemic.
573 It would additionally be important to conduct similar studies on other important emerging vaccines
574 such as the human papillomavirus (HPV), human immunodeficiency virus (HIV) and malaria
575 vaccines to determine how vaccine attitudes vary depending on the type of vaccine.

576

#### 577 Conclusion

578 This study adds to the health disparities literature, demonstrating how the COVID-19
579 pandemic exacerbated health disparities in this country. It is vital that policies should address
580 socioeconomic factors to help reduce these disparities [18]~~(Williams et al., 2022)~~. A paper by
581 Strully et al~~. (2021)~~ mentioned that some states had put into place strategies to increase equity
582 while distributing vaccines [28]. However, they note that for this to work, stakeholders need to
583 engage the community and gain their trust [28]~~(Strully et al., 2022)~~. In summary, the driving forces
584 behind vaccine hesitancy may be better understood when categorized into behavioral, biological,
585 sociocultural, and healthcare, and political domains. By applying the NIMHD research framework
586 lens to vaccine hesitancy, it is apparent that there are many opportunities for policymakers and
587 other stakeholders to implement interventions that address different racial/ethnic issues among
588 vaccine uptakes. We encourage future studies to explore the intersectionality of different
589 sociodemographic variables and their impacts on health disparities regarding vaccine hesitancy.

590

591

592

593

594

595

596

597

598

599

600

601

602

**Formatted:** Font: 12 pt

603 **Acknowledgements:** We recognize and acknowledge all of the hard work of the entire Alliance
604 for Vaccine Literacy team, including: Brianne Vaught, Sage Vu, Meccah Jarrah, Forest Melton,
605 Veena Raghuraman, Kerry Johnson, Adria Vasquez, Gloria Villa Barbosa, Shailesh Advani,
606 Benjamin Pope, Abidemi Olubukunmi Okechukwu, Anna Karina Ahmed, Anna Teresa Valencia,
607 Ava Neddermeyer, Beatrice Krauss, Bianca Patricia Carrasco, Brianna Catherine Rooney, Carlee
608 May-Qing Bettermann, Cori Cantin, Danielle Hutchens, Deepa Katuri, Dominique Marie Jordan,
609 Elena Hortencia Campos, Griselda Ruiz-Braun, Harrison Schmitt, Jacob Marczak, Kai (Xiao)
610 Glahn, Karl Krupp, Kelly Wentzel, Lady Dorothy Avila Elli, Mary Kinkade, Maryam Reyad,
611 Miguel Lopez, Pati Monterrey, Paula Garcia, Priscilla McGrath, Purnima Madhivanan, Rachel
612 Cummings, Rachel Zambrano, Ryan Jasareno Gonzales, Samantha Joanne Werts, Sonja
613 Velickovic, Stephanie Mallahan, Riley Sena, Kate Chambers, Kaela Liddle, Katie Donovan,
614 Makayla Lang, Nadia Flores, Martha Moore-Monroy, and Elsa Claire Bautista.

#### 615 Funding

616 This study was conducted using PM’s start-up funds. The Pima County Health Department
617 provided some funds to aid data collection. The content is solely the responsibility of the authors
618 and does not necessarily represent the official views of the funders. MBN was supported by the
619 National Institutes of Health, Fogarty International Center/ Global Health Emerging Scholars
620 Fellowship (D43TW010540). There was no additional external funding received for this study.
621 The content is solely the responsibility of the authors and does not necessarily represent the
622 official views of the funders.

623

624

#### 625 References

626 ~~Al-Jayyousi, G., Sherbash, M., Ali, L., El-Heneidy, A., Alhussaini, N., Elhassan, M., & Nazzal,~~
627 ~~M. (2021). Factors Influencing Public Attitudes towards COVID-19 Vaccination: A~~
628 ~~scoping Review Informed by the Socio-Ecological Model.~~ *~~Vaccines;~~* ~~9; 548-574.~~
629 ~~Center for Disease Control and Prevention. (2025). CDC Museum Covid-19 Timeline.~~
630 ~~Centers	for	Disease	Control	and	Prevention.~~	[~~https://www-cdc-~~](https://www-cdc/)
631 ~~gov.translate.goog/museum/timeline/covid19.html?_x_tr_hl=id&_x_tr_pto=tc&_x_tr_sl=~~
632 ~~en&_x_tr_tl=id~~
633 ~~Center for Disease Control and Prevention. (2025). Trends in United States COVID-19 Deaths,~~
634 ~~Emergency Department (ED) Visits, and Test Positivity by Geographic Area. CDC.~~
635 [~~https://covid.cdc.gov/covid-data-tracker/?#trends_totaldeaths_select_00~~](https://covid.cdc.gov/covid-data-tracker/?&trends_totaldeaths_select_00)
636 ~~Clarke, V., & Braun, V. (2017). Thematic analysis. The journal of positive psychology, 12(3),~~

637	~~297-298.~~

638 ~~Dedoose Version Dedoose-9.2.006, cloud application for managing, analyzing, and presenting~~
639 ~~qualitative and mixed method research data (2021). Los Angeles, CA: SocioCultural~~
640 ~~Research Consultants, LLC~~ [~~www.dedoose.com~~](http://www.dedoose.com/)~~.~~
641 ~~Dror, A., Eisenbach, N., Taiber, S., Morozov, N., Mizzrachi, M., Zigron, A., Srouji, S., & Sela, E.~~
642 ~~(2020). Vaccine Hesitancy: the next challenge in the fight against COVID-19.~~ *~~European~~*
643 *~~Journal of Epidemiology;~~* ~~35; 775-779.~~
644 ~~Gatwood, J., McKnight, M., Fiscus, M., et al. (2021).Factors influencing likelihood of COVID-19~~
645 ~~vaccination: A survey of Tennessee adults.~~ *~~American Journal of Health System Pharm;~~*
646 ~~78(10); 879-889.~~
647 ~~Halstead, N., McKay, R., & Lewis, G. (2022). COVID-19 and Seasonal Flu Vaccination~~
648 ~~Hesitancy: Links to Personality and General Intelligence in a Large UK Cohort.~~ *~~Vaccine;~~*
649 ~~40; 4488-4495.~~
650 ~~Mantina, N., Ngaybe, M., Johnson, K., Velickovic, S., Magrath, P., Gerald L., Krupp, K., Krauss,~~
651 ~~B., Perez,Velez, C., & Madhivana, P. (2022). Racial/Ethnic Disparities in Influenza Risk~~
652 ~~Perception and Vaccination Intention Among Pima County Residents in Arizona.~~ *~~Human~~*
653 *~~Vaccines & Immunotherapeutic;~~*~~18(7);1-8.~~
654 ~~Nikolovski, J., Koldijk, M., Weverling, G. J., Spertus, J. et al. (2021). Factors indicating intention~~
655 ~~to vaccinate with a COVID-19 vaccine among older U.S. adults.~~ *~~PLOS ONE;~~* ~~16(5); 1963~~
656 ~~Privor-Dumm, L and King, T. (2020). Community-based Strategies to Engage Pastors Can help~~
657 ~~Address Vaccine Hesitancy and Health Disparities in Black Communities.~~ *~~Journal of~~*
658 *~~Health Communication;~~* ~~25(10); 827-830.~~
659 ~~Reiter, P., Pennell, M., & Katz, M. (2020). Acceptability of a COVID-19 Vaccine Among Adults~~
660 ~~in the United States: How many people would get vaccinated?~~ *~~Vaccines;~~* ~~38; 6500-6507.~~
661 ~~Soares, P., Rocha, J., Moniz, M., Gama, A., Laires, P. et al. (2021). Factors Associated with~~
662 ~~COVID-19 Vaccine Hesitancy.~~ *~~Vaccines;~~* ~~9; 300-315.~~
663 ~~Strully, K., Harrison, T., Pardo, T., & Carleo-Evangelist, J. (2021). Strategies to Address COVID-~~
664 ~~19 Vaccine Hesitancy and Mitigate Health Disparities in Minority Populations.~~ *~~Frontiers~~*
665 *~~in Public Health;~~* ~~9; 1-7.~~
666 ~~Webb Hooper, M., Napoles, A., and Perez-Stable, E. (2021). No Populations Left Behind: Vaccine~~
667 ~~Hesitancy and Equitable Diffusion of Effective COVID-19 Vaccines.~~ *~~J Gen Int Med.;~~*
668 ~~2130-2133.~~
669 ~~World Health Organization. (2019). Top Ten Threats to Global Health in 2019. WHO.~~
670 [~~https://www.who.int/news-room/spotlight/ten-threats-to-global-health-in-2019~~](https://www.who.int/news-room/spotlight/ten-threats-to-global-health-in-2019)
671 ~~Williams, A., Clayton, H., & Singleton, J. (2022). Racial and Ethnic Disparities in COVID-19~~
672 ~~Vaccination Coverage: The Contribution of Socioeconomic and Demographic Factors.~~
673 *~~American Journal of Preventative Medicine;~~* ~~62(4); 473-482.~~
674 ~~Willis, D., Anderson, J., Montgomery, B., Selig, J., Shah, S. et al. (2023). COVID-19 Vaccine~~
675 ~~Hesitancy and Experiences of Discrimination Among Black Adults.~~ *~~Journal of Racial and~~*
676 *~~Ethnic Disparities;~~* ~~10; 1025-1034.~~
677 ~~Yasmin, F., Najeeb, H., Moeed, A., Naeem, U., Asghar, M. S., Chughtai, N. U., ... & Pakpour, A.~~
678 ~~H. (2021). COVID-19 vaccine hesitancy in the United States: a systematic review.~~
679 *~~Frontiers in public health~~*~~,~~ *~~9~~*~~, 770985.~~
680 ~~Zimmerman, T., Shiroma, K., Fleischmann, K., Xie, B., Jia, C., Verna, ., & Lee, M. (2023).~~
681 ~~Misinformation and COVID-19 Vaccine Hesitancy.~~ *~~Vaccine;~~* ~~41; 136-144.~~
682 **~~Appendix 1.~~** ~~Codebook and definitions~~

683

| **~~Code~~** | **~~Definition~~** |
| --- | --- |
| **~~Biological~~** | ~~Any influence pertaining to an individual’s biological response to internal & external~~ ~~factors. This includes age, gender identity, personal immunity, genetic predispositions,~~ ~~pre-existing medical conditions, sanitation, or environmental exposures.~~  **~~Typical exemplars:~~**  ~~Biological Vulnerability/Mechanisms~~ ~~Caregiver-Child Interaction~~  ~~Family Microbiome~~ ~~Community Illness/Exposure~~ ~~Herd Immunity~~  ~~Sanitation~~ ~~Immunization~~ ~~Pathogen Exposure~~ |
| ~~Biological~~ ~~Vulnerability and~~ ~~Mechanisms~~ | ~~Quotes relating to someone having a biological reason (like a preexisting condition) that~~ ~~makes them particularly vulnerable to a disease.~~  ~~Genetic and physiological factors that affect an individual's susceptibility to diseases and~~ ~~health conditions.~~  ~~E.g. Mentioning that they are older and therefore are more likely to get COVID-19 and~~ ~~therefore need to vaccinate.~~ |
| ~~Caregiver-Child~~ ~~Interaction~~ | ~~Quotes related to wanting to get (themselves or a child) vaccinated (or not) because of~~ ~~their relationship with their caregiver or child.~~  ~~E.g. Having their child get vaccinated because of a feeling that they need to protect their~~ ~~child from getting COVID~~ |
| ~~Family Microbiome~~ | ~~Quotes related to the family having a higher likelihood of sharing diseases, bacteria,~~ ~~viruses, etc. because of their close proximity and interactions.~~  ~~E.g. Needing to get vaccinated to protect their family because they know they will have~~ ~~a higher likelihood of passing the disease on to their family if they go home with the~~ ~~disease~~. |
| ~~Community Illness~~ ~~Exposure~~ | ~~Quotes related to their community generally having the disease or being at risk of getting~~ ~~the disease, or needing to get vaccinated to avoid exposure at a community level.~~  ~~E.g. Choosing to get vaccinated because they are worried that the community is ill and~~ ~~that they may be exposed. Being out in the workforce making them more exposed to~~ ~~getting ill.~~ |
| ~~Herd Immunity~~ | ~~Quotes related to herd immunity, the indirect protection from an infectious disease that~~ ~~happens when a population is immune either through vaccination or immunity developed~~ ~~through previous infection.~~  ~~E.g. Discussion about how many people need to get vaccinated in order to stop the spread.~~ ~~Or quotes related to them feeling that they need to get vaccinated to protect others in their~~ ~~community~~. |

| ~~Sanitation~~ | ~~Quotes related to staying clean or sanitizing (remember this is at a societal level).~~  ~~Societal-level factors such as public health policies and practices that reduce pathogen~~ ~~exposure and disease spread.~~  ~~E.g. The need for the society to be using gloves, PPE, and disinfectants.~~ |
| --- | --- |
| ~~Immunization~~ | ~~Quotes directly related to the need for the society to get immunized against COVID-19~~ ~~(remember this is at a societal level).~~  ~~E.g. The need for the society at large to get vaccinated.~~ |
| ~~Pathogen Exposure~~ | ~~Quotes related to being worried about being exposed to the COVID-19 pathogen, in~~ ~~higher level terms than the community - conceptually thinking of the pandemic for~~ ~~instance~~. |
| **~~Behavioral~~** | ~~How an individual, or group of individuals, cope with health related behaviors and~~ ~~changes in their environment. Their strategies and ways of coping. How they may be~~ ~~affected by the behavior of others. How they respond to different stressors, respond to the~~ ~~behaviors of others. The driving force behind the personal choice that people make every~~ ~~day. The actions and reactions that one takes in response to situations they encounter.~~  **~~Typical exemplars:~~**  ~~Health Behaviors~~ ~~Coping Strategies~~  ~~Family/Community/School/Work Functioning~~ ~~Policies and Law~~ |
| ~~Health behaviors~~ | ~~Quotes relating to health-related behaviors that they do to prevent exposure or protect~~ ~~themselves and be healthy (or also that they don’t do)~~.  ~~E.g. Eating healthy, exercise, handwashing, or the opposite - smoking, being sedentary,~~ ~~etc.~~ |
| ~~Coping styles~~ | *~~Coping is defined as the thoughts and behaviors mobilized to manage internal and~~ ~~external stressful situations.~~*  ~~In this code, include quotes related to how someone manages stress by engaging in a~~ ~~particular behavior (religion, problem-focused, emotion-focused, support-seeking, and~~ ~~meaning-making coping), in particular in how it impacts their vaccination.~~  ~~E.g. Praying, looking to friends for support, drinking alcohol, etc.~~ |
| ~~Family functioning~~ | *~~Family functioning has been defined as “the interactions of family members that involve~~ ~~physical, emotional and psychological activities” (Commonwealth of Kentucky, 2001)~~ ~~and “the process by which the family operates as a whole, including communication and~~ ~~manipulation of the environment for problem solving” (Mosby)~~*  ~~In this code, include quotes related to how the dynamics within a family influence~~ ~~behavior and overall health~~  ~~E.g. Someone mentions that their family always got their shot and therefore they will~~ ~~continue to get their vaccinations.~~ |

| ~~School/Work~~ ~~functioning~~ | ~~Quotes related to how school or work activities, interactions and policies might impact~~ ~~willingness to get vaccinated.~~  ~~E.g. Policies in the school or work promoting/requiring vaccination.~~ |
| --- | --- |
| ~~Community~~ ~~Functioning~~ | ~~Quotes related to how the participant’s community’s general normal activities and~~ ~~interactions impacts their willingness to get vaccinated.~~  ~~E.g. They mention that they need to get vaccinated in order to go out into their community~~ ~~without any issues~~. |
| ~~Policies and Laws~~ | ~~Quotes related to how policies at a societal level might impact willingness to get~~ ~~vaccinated~~.  ~~E.g. The need for laws in the USA that require vaccination in general or for certain~~ ~~activities such as flying or going on cruises.~~ |
| **~~Physical/Built~~ ~~Environment~~** | ~~Includes built or altered components related to the environment we create around~~ ~~ourselves. This can include work/school, personal living situations, and community~~ ~~involvement. An individual's personal environment encompasses the effects they have to~~ ~~participate in, residence and interactions.~~  **~~Typical exemplars:~~ ~~Environments~~**   - **~~Personal~~** - **~~Household~~** - **~~School~~** - **~~Community~~**   ~~Societal Structure~~ ~~Community Resources~~ |
| ~~Personal Environment~~ | ~~The immediate physical surroundings that an individual interacts with daily, that is~~ ~~personal to them (e.g. their room, their personal car).~~  ~~E.g. The participant mentions they have a routine where they keep PPE in their car so that~~ ~~they always have it around in case they need it.~~ |
| ~~Household~~ ~~Environment~~ | ~~Quotes related to physical settings within households (parts of the house that are shared~~ ~~with other household members) that impact health and well-being.~~  ~~E.g. They have a station in their garage where they sanitize their groceries before bringing~~ ~~them into the house.~~ |
| ~~School/Work~~ ~~Environment~~ | ~~Quotes related to how school or work environments might impact willingness to get~~ ~~vaccinated~~.  ~~E.g. Posters promoting vaccination on the walls in the workplace.~~ |
| ~~Community~~ ~~Environment~~ | ~~Quotes related to how the community’s physical environment might impact willingness~~ ~~to get vaccinated~~.  ~~E.g. Community sets up stations for vaccination in the neighborhood so people can access~~ ~~it more easily, posters at the pharmacy declaring if they have vaccines.~~ |

| ~~Community Resources~~ | ~~Quotes related to the availability of community resources (such as funding or health-related items like vaccines) or lack of such resources that helps or hinders vaccination~~ ~~efforts~~.  ~~E.g. Community provides support for COVID-19 testing. Community has testing centers~~ ~~where people can go.~~ |
| --- | --- |
| ~~Societal Structure~~ | ~~Quotes related to the organization and distribution of resources and opportunities within~~ ~~a society that shape the physical and built environment~~ |
| **~~Sociocultural~~ ~~Environment~~** | ~~Any individuals with shared beliefs and the influence of their individual beliefs on others~~ ~~that shape the behavior, attitudes, and interactions of people within a society.~~  **~~Typical exemplars:~~**  ~~Societal Structure/Social Norms/Sociodemographic characteristics~~ ~~Discrimination (Interpersonal and Response)~~  ~~Community/ Family/ Peer Norms~~ ~~Local Structural~~  ~~Social Networks~~ ~~Limited English~~ ~~Cultural Identity~~  **~~Excludes:~~** ~~workplace requirements~~ |
| ~~Sociodemographic~~ ~~characteristics~~ | ~~Quotes related to demographics (age, gender, race, ethnicity, etc.) as a facilitator or barrier~~ ~~to vaccination.~~ |
| ~~Limited English~~ | ~~Quotes related to people who have limited English literacy having issues related to getting~~ ~~vaccinated~~. |
| ~~Cultural Identity~~ | ~~Quotes related to people of a cultural identity having issues or advantages to getting~~ ~~vaccinated~~. |
| ~~Response to~~ ~~Discrimination~~ | ~~Quotes related to someone’s response to an issue of discrimination related to getting~~ ~~vaccinated or that leads to them being more or less likely to get vaccinated.~~ |
| ~~Social Networks~~ | ~~Quotes related to someone’s social networks in general (not family or peer specific) such~~ ~~as social media and how it related to getting vaccinated or being willing to get vaccinated.~~  ~~E.g. Facebook or Twitter impacting their views about vaccines.~~ |
| ~~Family/Peer Networks~~ | ~~Quotes related to family or peer networks and how they impact the participant’s~~ ~~vaccination~~.  ~~E.g. Discussion of how family or peers and how they influence their attitudes about~~ ~~vaccines~~. |
| ~~Interpersonal~~ ~~Discrimination~~ | ~~Quotes related to barriers to vaccination related to interpersonal discrimination issues.~~ |

|  | ~~E.g. A nurse treated someone badly because of their race and therefore the person doesn’t~~ ~~want to get vaccinated~~. |
| --- | --- |
| ~~Community Norms~~ | ~~Quotes related to facilitators or barriers to vaccination related to community norms.~~  ~~E.g. “Everyone in our community gets vaccinated so I do too.”~~ |
| ~~Local Structural~~ ~~Discrimination~~ | ~~Quotes related to discrimination within the local community at a structural level that~~ ~~causes someone not to get vaccinated.~~  ~~E.g. The community has issues related to race that causes some communities to do better~~ ~~than others in this neighborhood and therefore some get vaccinated and others don’t.~~ |
| ~~Social Norms~~ | ~~Quotes related to social norms at large that either promote or are barriers to vaccination.~~  ~~E.g. We as a democratic party are getting vaccinated.~~ |
| ~~Societal Structural~~ ~~Discrimination~~ | ~~Quotes related to societal level discrimination that are facilitators or barriers to~~ ~~vaccination~~.  ~~E.g., “Anti-vaxxers are all republicans.” Redlining causing issues….? Communities of~~ ~~color having differences in ability to get vaccinated due to historical and structural issues.~~ |
| **~~Health Care System~~** | ~~Individuals with shared interactions, attitudes, and experiences with healthcare workers,~~ ~~policies, and settings~~  **~~Typical exemplars:~~** ~~Insurance Coverage~~ ~~Health Literacy~~  ~~Treatment Preferences~~  ~~Patient-Clinician Relationship~~ ~~Medical Decision Making~~ ~~Services Availability/Safety Net~~ ~~Quality of Care~~  ~~Health Care Policies~~ |
| ~~Insurance Coverage~~ | ~~Issues related to someone’s insurance that either facilitate or act as barriers to vaccination.~~ |
| ~~Health Literacy~~ | ~~Issues related to someone not understanding health-related issues and therefore not being~~ ~~interested or willing to get vaccinated~~.  ~~E.g. Misinformation issues. Not understanding how viruses or vaccination works.~~ |
| ~~Treatment Preferences~~ | ~~Issues related to preferences in receiving the vaccine acting as a barrier or facilitator to~~ ~~getting vaccinated~~  ~~E.g. “I don’t like shots”~~ |
| ~~Patient-Clinician~~ ~~Relationship~~ | ~~Issues related to the patient’s relationship with their health care provider that either~~ ~~facilitate or get in the way of their vaccination or vaccine acceptance.~~  ~~E.g. “My personal doctor told me to get vaccinated so I did.”~~ |
| ~~Medical Decision-Making~~ | ~~Issues related to making decisions in a medical context (calculating health decisions) that~~ ~~interfere with or facilitate vaccination.~~ |

|  | ~~E.g. “I want to get vaccinated because I am worried about being hospitalized because I~~ ~~might get sick from other diseases.” “I am worried about the side effects.”~~ |
| --- | --- |
| ~~Availability of~~ ~~Services~~ | ~~Issues related to the availability of vaccines interfering with or facilitating vaccination.~~  ~~E.g. Access related issues to getting vaccinated, ‘the pharmacy had a sign that said~~ ~~“vaccines available” but they didn’t have any.’~~ |
| ~~Safety Net Services~~ | ~~Issues related with~~ **~~policies and programs that help individuals and families manage~~ ~~risk and volatility, protect them from poverty and inequality, and help them to~~ ~~access economic opportunities~~** ~~that either facilitate or act as barriers to vaccination.~~  ~~E.g. Trusting the local health department,~~ |
| ~~Quality of Care~~ | ~~Issues related to the quality of care one receives in a health care setting either facilitating~~ ~~or interfering with vaccination.~~ |
| ~~Health Care Policies~~ | ~~Health care policy-related issues that impact vaccination.~~ |
| **~~Other~~** |  |
| ~~Politics~~ | ~~Political issues, especially considering Federal administration changes, etc.~~ |

684

685

686 **~~Supplemental File 1~~**
687 ~~Focus Group Discussions with Hispanic and White Males~~
688 **~~Table 1~~** ~~displays the results of the focus group discussion with Hispanic and white male~~
689 ~~participants. In these discussions, conversations appeared to focus primarily around the behavioral~~
690 ~~and health care systems domains. For instance, under the Behavioral domain, participants~~
691 ~~mentioned that people in their community had mentioned they would “wait and see.” A participant~~
692 ~~in the group mentioned that they wanted to protect others in the community, but added a caveat~~
693 ~~that they do not feel a “national sentiment”, indicating that there was a political motive promoting~~
694 ~~vaccination that he did not agree with. Under the interpersonal level, the participants mentioned~~
695 ~~having a habit of getting vaccinated that was instilled at a young age by their family, perhaps~~
696 ~~leading to their willingness to get vaccinated again against COVID-19. Additionally, policies and~~
697 ~~laws mandating vaccines to get documents such as green cards appeared effective in influencing~~
698 ~~vaccination behavior in this group.~~
699 ~~Under the Health Care Systems domain, participants mentioned lower trust in the COVID-19~~
700 ~~vaccine because it was so new, and they didn’t know much about it. In making their medical~~
701 ~~decision, they additionally mentioned trusting people doing the~~ *~~“actual science”~~* ~~and weighing the~~
702 ~~efficacy percentages when considering when a vaccine would be acceptable for them to take.~~
703 ~~Finally, under health care policies, participants mentioned several times that they were concerned~~
704 ~~about how quickly the vaccine was developed and worried about the COVID-19 vaccine being~~
705 ~~rushed to the public compared to other vaccines. As one participant said,~~
706 *~~“There's a lot of mistrust. And I, and I think you see that because it's, it's kind of difficult~~*
707 *~~to understand how a vaccine could be developed so fast relatively to, you know, how fast~~*
708 *~~a normal, I guess, a vaccine normally is developed which takes years. And you know I do.~~*
709 *~~I do believe in innovation and technology….” (p12)~~*
710 ~~Under the biological domain, participants demonstrated high scientific literacy and an~~
711 ~~understanding of how herd immunity works, using scientific words such as “vector.” This~~
712 ~~participant mentioned that they knew they needed to get vaccinated to reduce risk for others. Under~~
713 ~~the Sociocultural environment, participants mentioned a higher degree of trust of the local~~
714 ~~community compared to the national government or other higher level health organizations. There~~
715 ~~was no mention of issues which fit under the Physical/built environment domain.~~

716

717

718

719

720

721	**~~Table 1.~~** ~~Hispanic and white male focus group participant findings mapped onto the NIMHD Health Disparities framework (nFGD=1,~~

722	~~nP=4)~~

723

724

**~~LEVELS OF INFLUENCE~~**

|  | **~~Individual~~** | **~~Interpersonal~~** | **~~Community~~** | **~~Societal~~** |
| --- | --- | --- | --- | --- |
| **~~Biological~~** |  |  | ~~p13: But now with Covid-19 going on.~~ **~~I'm, I'm~~ ~~definitely more aware of how People access~~ ~~vectors for diseases~~** ~~like influenza and how the~~ ~~combination of an influencer in the fall, could~~ ~~Real big problems. So now I'm definitely~~ ~~considering getting it. I'm going. I think I'm~~ ~~probably going to because I'm more aware and~~ ~~it seems like it might be a bigger risk to others if~~ ~~I don't get vaccinated (Herd Immunity)~~ |  |
| **~~Behavioral~~** | ~~p15: I've heard kind of the opposite direction like people~~ ~~going in the opposite direction with Covid as it pertains to~~ ~~the Covid vaccine saying like people that are pro-vaccination saying like, I'm not going to be… I'm not~~ ~~going to get the vaccine this as soon as it comes out.~~ **~~I'm~~ ~~going to wait and see if people get sick.~~** ~~And then I'm~~ ~~going to, and then I'll get it. (Health Behaviors)~~  ~~p13: That's the whole reason why I would get the vaccine~~ ~~would be, because~~ **~~it would help protect the people~~ ~~around me~~**~~. I don't have that second national sentiment~~ ~~that people really feel, but that's how I feel. (Health~~ ~~Behavior)~~ | ~~p12: When I was younger,~~ **~~I went with my siblings~~** ~~to to~~ ~~get vaccinated (Family functioning)~~  ~~p13: Um, and I think this is kind of a habit that my dad~~ ~~mostly passed on to me because he, like, never really went~~ ~~to the doctor and, like,~~ **~~just kind of he was healthy, he~~ ~~would get a cold once a year and get over it. And he was~~ ~~always fine.~~** ~~Um, so I think I've kind of adopted that~~ ~~mentality that's why. (Family Functioning)~~ |  | ~~p14: It was~~ **~~part of the process for getting~~ ~~a green card~~**~~. I am from Japan, and I didn't~~ ~~have certain vaccinations that required to be~~ ~~to the residents. (Policies and Laws) (p 14)~~ |
| **~~Sociocultural~~ ~~Environment~~** |  | ~~p15: I think if people can hear it from~~ **~~people trust their~~ ~~neighbors and trust their local government, a lot more~~ ~~than their state and national government.~~** ~~And if they~~ ~~can hear it from local representatives ward officers. (Social~~ ~~Network/Family/Peer Norms~~**~~)~~**  ~~p15:~~ **~~Friends and family, then, that will change a lot of~~ ~~hearts and minds~~** ~~more so than anything else (Social~~ ~~Network/Family/Peer Norms~~ |  |  |
| **~~Health Care~~ ~~Systems~~** | ~~p15: You know, like they don't trust. They don't trust the~~ ~~three-stage system that they use to, to develop vaccines.~~ ~~Least, at least~~ **~~when it's something so novel as Covid~~** ~~(Health Literacy)~~ | ~~p15: I was gonna say, I trust vaccines and~~ **~~I trust the~~ ~~people who are doing the, the actual science.~~**  ~~(Medical Decision-Making)~~ |  | ~~p12: The, the, the need for it, but it is~~ ~~concerning that it would be, you know,~~ ~~there's talks of it being developed so fast.~~ ~~When the most the fastest a vaccine has ever~~ ~~been developed, I believe, is around four~~ ~~years so cutting that down so fast is~~ **~~worrisome because you can really rush~~** ~~(Healthcare Policies)~~  ~~p12: Or the, the process is set for a reason.~~ ~~And~~ **~~it seems that it's trying to be fast~~ ~~tracked~~** ~~(Health Care Policies)~~ |
| **~~Health Outcomes~~** | **~~Individual Health~~** | **~~Family/Organizational Health~~** | **~~Community Health~~** | **~~Population Health~~** |

725 ~~Hispanic male focus group~~
726 **~~Table 2~~** ~~displays the findings for Spanish-speaking male focus group participants. At the~~
727 ~~individual level of influence, participants acknowledge biological, behavioral, and healthcare~~
728 ~~system domains of influence regarding their beliefs about the pandemic. Concern about getting~~
729 ~~sick influenced the behavioral response of one participant who stated,~~ *~~“This is the first time in my~~*
730 *~~life that I have got the flu vaccine and the reason I did it this year was because COVID is involved~~*
731 *~~in this whole problem. So, to prevent them from overlapping, I did it” (p:~~* ~~47). Participants~~
732 ~~acknowledged biological vulnerability and mechanisms, such as the importance of a healthy~~
733 ~~immune system and coping with the pandemic. Health literacy also was mentioned as an influence~~
734 ~~on their beliefs, illustrated by one focus group member who noted the role that not getting clear~~
735 ~~information about the pandemic caused them distress or confusion.~~
736 ~~At the interpersonal level of influence, the domains of influence with the most common~~
737 ~~themes were the sociocultural environment and health care systems. Family and peer norms as~~
738 ~~well as social networks of members of the focus group impacted the interpersonal relationships.~~
739 ~~Another individual from the focus groups showed how conflicting views of companies impacted~~
740 ~~the relationship with the healthcare system.~~
741 ~~The sociocultural environment and health care system were also recurring domains that~~
742 ~~represented the focus group responses. One individual explained how structural discrimination~~
743 ~~was having an impact on certain communities more than others, which is consistent with another~~
744 ~~focus group member, who stated that the availability of resources impacted their attitudes and~~
745 ~~behaviors regarding the pandemic. Lastly, at the societal level one participant stated that at the~~
746 ~~societal level this pandemic and the way information was shared also demonstrated discrimination~~
747 ~~at the societal level.~~
748 ~~Overall, from a sociocultural level the following quote suggests ethnic differences~~
749 ~~regarding how to behave during the pandemic,~~
750 *~~“I think that in the Hispanic environment, or so to speak, there is a little more privacy in~~*
751 *~~terms of people taking into account... Well, I am talking about the circle closest to me, and~~*
752 *~~they take into account that we must follow the rules precisely so as not to become infected.~~*
753 *~~For example, there are not so many parties, I don't know if they go to so many bars and all~~*
754 *~~that.” (p55).~~*

755

756

757	**~~Table 2.~~** ~~Hispanic male focus group participant findings mapped onto the NIMHD Health~~

758	~~Disparities framework (nFGD=2, nP=5, 3)~~

759	**~~LEVELS OF INFLUENCE~~**

|  | **~~Individual~~** | **~~Interpersonal~~** | **~~Community~~** | **~~Societal~~** |
| --- | --- | --- | --- | --- |
| **~~Biological~~** | ~~p56: I believe a lot in science, I believe a lot in science,~~ ~~but i also believe a lot in nature,~~ **~~I believe that we are well~~ ~~designed, so we can convert many diseases (~~**~~Biological~~ ~~Vulnerability and Mechanisms)~~  ~~p55: So that's if I get the vaccine...~~ **~~I'd rather be healthy,~~ ~~have an immune system~~**~~. (Biological Vulnerability and~~ ~~Mechanisms)~~ |  |  | ~~p 50:~~ **~~whether you got it or not, you have to take~~ ~~care of yourself to not get other people sick.~~** ~~(Sanitation/Pathogen Exposure)~~ |
| **~~Behavioral~~** | ~~p55: …~~**~~when he arrives from his work, he takes off all~~ ~~his clothes~~** ~~and that is a constant that has been evolving,~~ ~~but not as much as we would like that he is still very~~ ~~panicky. (Health Behaviors / Coping Strategies)~~  ~~p50: what I would do is isolate myself, you understand me,~~ ~~from my family as well to not get other people sick and~~ ~~that’s what we are doing right now (Health Behaviors)~~ |  |  |  |
| **~~Physical/Built~~ ~~Environment~~** |  | ~~p55: Hispanic Whether by luck or whatever it's called~~ ~~right... She immediately took the test and came back~~ ~~negative. But if we had hidden this problem or not infected~~ ~~us and not to speak clearly why they will say it would have~~ ~~infected other people then that is not to be done~~ ~~(Home/work Environment)~~ |  |  |
| **~~Sociocultural~~ ~~Environment~~** |  | ~~p55: Well, I am talking about~~ **~~the circle closest to me~~**~~, and~~ ~~they take into account, that~~ **~~we must follow the rules~~ ~~precisely so as not to become infected~~** ~~(Family/Peer~~ ~~Norms)~~  ~~p56: …starting with the memes. Yes, you've heard about the~~ ~~memes? they put information on Facebook that they're~~ ~~getting a vaccine and then they take out an eye, which is~~ ~~funny, but~~ **~~those funny things are getting into people's~~ ~~brains, and there's this panic about getting a vaccine~~** ~~(Social Networks)~~ | p55: And then make other people known [their COVID status], that they have the problem, because unfortunately now **out of shame we are hiding part of this process** and that's why more people are becoming infected (Community Norms /Local ~~Structural Discrimination)~~ | ~~p56:~~ **~~They have done so much harm to society, to~~ ~~the people,~~** ~~because that's why they say the people die~~ ~~for lack of information. (Societal Structural~~ ~~Discrimination)~~  ~~p55: Ah! I think that right now the~~ **~~media is~~ ~~polarized, there is misinformation or there is false~~ ~~information~~**~~… I think that it is good to find out~~ ~~through those who are in direct contact with the~~ ~~problem, but not through the television media that~~ ~~sometimes tell lies, so you have to find out through~~ ~~doctors (Social Norms)~~ |
| **~~Health Care Systems~~** | ~~p55: …unfortunately~~ **~~the television stations often distort~~ ~~the informatio~~**~~n according to their criteria. And that is the~~ ~~misinformation that exists now (Health Literacy)~~ | ~~p55: In such a way that everything that is being exposed at~~ ~~the level of companies is being opposed,~~ **~~but the health~~ ~~professionals who want to convince us that the vaccine~~ ~~is going to be the best for the majority of humans, then~~ ~~that is the problem.~~** ~~(Patient-Clinician Relationship)~~ | p 55: So, if a vaccine comes, we are going to say like Covid, if it is already established, it has credibility, it has everything, so the **person will probably have long lines to get the vaccine** (Availability of ~~Services)~~  p 51: So, if someone sees something that worries them the first thing they should do is **go and find good information. Talk with their doctor, talk to the health department, go to good websites on the internet that are like the Mayo Clinic or other responsible groups that can give good information** ~~(Safety net services)~~ | **~~p 48:~~** ~~Look, my opinion on this is that my community~~ ~~still has very, very retroactive roots.~~ **~~Very, very~~ ~~ingrained roots that they still don’t believe in~~ ~~vaccines, they don’t believe~~**  ~~(Structural Discrimination)~~ |
| **~~Health Outcomes~~** | **~~Individual Health~~** | **~~Family/Organizational Health~~** | **~~Community Health~~** | **~~Population Health~~** |

760

761 ~~Hispanic female focus group participants~~
762 **~~Table 3~~** ~~displays the results from Hispanic Female focus groups. In these discussions,~~
763 ~~participants talked about issues related to health literacy, demonstrating a concern with reliability~~
764 ~~of information they found regarding health. Medical decision-making was a common area of~~
765 ~~conversation as well, with an emphasis on calculating the risk of taking a vaccine; they were~~
766 ~~concerned with determining the “Pros and Cons” wondering how the vaccine would affect their~~
767 ~~body. For example, one participant mentioned that they would weigh the risks of side effects, but~~
768 ~~if they would have their life saved they would get the vaccine. However, they also expressed fears~~
769 ~~of getting COVID-19 and possibly~~ *~~“losing a job, being hospitalized, not being able to go out” (p~~*
770 *~~37).~~* ~~They expressed mistrust of local health department recommendations; this was mostly due to~~
771 ~~the fact that the department told them that they do not know much when asked for guidance:~~
772 *~~“The ones who did the exam said she should stay seven more days after the test. The~~*
773 *~~pediatrician tells me that she should stay ten more days. Who is right? They told me, “Well,~~*
774 *~~in reality we don’t know.” So, if you are trusting someone and they tell you “well we don’t~~*
775 *~~know because it’s new.” Who are you going to trust?” (p36)~~*
776 ~~and the inconsistency of recommendations given across the health care system at the time:~~
777 *~~“[T]hey confuse people more like that and they scare them more, because they are the~~*
778 *~~experts they should be constant.” (p37)~~*
779 ~~On the other hand, participants mentioned trust in other sources like family members, family~~
780 ~~doctors, and books they have read.~~
781 ~~Under the sociocultural environment domain, one participant mentioned their trust in~~
782 ~~traditional and alternative medicine. Another participant mentioned that they also have a culture~~
783 ~~of listening to their mothers and so therefore if their mother told them to do a certain health~~
784 ~~behavior like get vaccinated, it would be a part of their culture to listen to them.~~
785 ~~Under the behavioral domain, participants in this group mentioned that getting a vaccine at no cost~~
786 ~~at their workplace was a motivating factor to getting the vaccine. At the interpersonal level,~~
787 ~~participants mentioned that family also influenced them to get the vaccine to help protect those~~
788 ~~with predisposing conditions such as asthma. On a similar note, participants at the biological level~~
789 ~~mentioned that it was important to get vaccinated if a person has a medical condition such as~~
790 ~~cancer. However, a few participants said that they felt healthy themselves, one participant stating,~~
791 *~~“I am a very healthy person. I have never gotten sick, and I haven’t had the need”~~* ~~(p3) which may~~
792 ~~have made them complacent to COVID-19’s risk and perhaps less likely to be interested in getting~~
793 ~~vaccinated for themselves.~~
794 ~~In summary, there appeared to be a big motivation to get a future COVID-19 vaccine in~~
795 ~~these discussions, especially to protect their families. However, these participants did not appear~~
796 ~~to be interested in getting it or encouraging anyone else to get it, at least until there was a~~
797 ~~demonstrated clear benefit to getting it and many others had gotten it before them.~~

798

799

800	**~~Table 3.~~** ~~Hispanic female focus group participant findings mapped onto the NIMHD Health~~

801	~~Disparities framework (nFGD=2, nP=6, 7)~~

802	**~~Levels of Influence~~**

|  | **~~Individual~~** | **~~Interpersonal~~** | **Community** | **~~Societal~~** |
| --- | --- | --- | --- | --- |
| **~~Biological~~** | ~~p40: I know that I am taking care of myself. I wouldn't be too~~ ~~scared if there is a flu outbreak.~~  ~~p5:~~ **~~I didn’t get a vaccine every year,~~** ~~until…until after we~~ ~~knew that he had asthma so we could protect him because he~~ ~~would get the flu really bad. (Biological Vulnerability and~~ ~~Mechanism)~~ | ~~p40: If we didn’t have a mask, we would use a rag so we didn’t cough~~ ~~and spread the virus to the family. It's what we’ve always done. (Family~~ ~~Microbiome)~~  ~~p36: Immediately have hand sanitizer, or wash her hands, and don’t touch~~ ~~anything, don’t touch your face, and don’t get down, constantly. You~~ ~~don’t pay attention to what you are going to do because you’re watching~~ ~~them. Because there is fear-- my fear is that she infects my parents~~ ~~because they are vulnerable. ((Family Microbiome)~~ |  |  |
| **~~Behavioral~~** | ~~p39: So for me~~ **~~the best thing is prevention~~**~~. That is washing~~ ~~your hands, it’s the simplest thing without a lot of chemicals,~~ ~~just soap, alcohol, and more than anything prevention. Like~~ ~~taking care of yourself, and maintaining distance, your face~~ ~~mask, and have the necessary preventions. (Health Behavior)~~ | ~~p4: …~~**~~at work they give it to me for free…~~** ~~(School/Work Functioning)~~  ~~p36: If I have to keep her in quarantine the rest of the year I’ll do it~~**~~, but~~ ~~I don’t want her to do what other people have done that they infect~~ ~~others without caring~~**~~. A lot of people have been infected that way.~~ ~~(Family functioning)~~ |  |  |
| **~~Physical/Built~~**  **~~Environment~~** |  |  |  |  |
| **~~Sociocultural~~ ~~Environment~~** | ~~p3:~~ **~~I also believe in traditional medicin~~**~~e, but I also believe~~ ~~in alternative medicine. (Cultural identity)~~  ~~p5: I also think we listen to moms. I still think of my mom:~~ ~~Honey, go get it, ok mom. Well for me it’s part of the culture~~ ~~I don’t know. (Cultural Identity)~~ | ~~p3: Ok.~~ **~~I wouldn’t encourage anybody~~** ~~because I think that people…~~ ~~everyone in particular has the right to choose. Especially about their~~ ~~body. I wouldn't encourage putting .... anybody a medication.~~ **~~It’s a~~ ~~personal decision; everyone has to choose.~~** ~~(Social Networks)~~ |  |  |
| **~~Health Care Systems~~** |  | ~~p36: I would need to know what is in the vaccine. What is the content of~~ ~~the vaccine? Because I have heard different versions~~**~~.~~** ~~(Medical Decision~~ ~~Making)~~  ~~p37:~~ **~~You’re scared of the results, losing a job, being hospitalized, not~~ ~~being able to go out,~~** ~~and other illnesses like depression that tends to~~ ~~happen when you’re isolated. (Medical Decision Making)~~ | p42: Well, I would ask where they get their information from, if they use trusted resources. Then I would yes, but if they can’t give a trusted source, then no. **Sometimes the local departments know a lot, sometimes they don’t.** ~~(Safety Net Services)~~ |  |
| **~~Health Outcomes~~** | **~~Individual Health~~** | **~~Family/Organizational~~ ~~Health~~** | **Community Health** | **~~Population~~ ~~Health~~** |

803

804

| 805 |  |
| --- | --- |
| 806 | ~~Black male focus group~~ |
| 807 | **~~Table 4~~** ~~displays the findings for AA male focus group participants. Participants used~~ |
| 808 | ~~examples resonating with the biological and behavioral domains of influence at the individual level~~ |
| 809 | ~~of influence to explain their attitudes and behaviors regarding the pandemic. One participant spoke~~ |
| 810 | ~~about their grandmother having a chronic condition that suggests biological vulnerability was~~ |
| 811 | ~~impacting their decision-making. The participants at the behavioral level stated that they would~~ |
| 812 | ~~take the vaccine because that would be the only way to determine the impact of side effects. Yet~~ |
| 813 | ~~many of the participants was afraid of side effects that may contribute to vaccine hesitancy. p53~~ |
| 814 | ~~stated~~ *~~“long term health effects….because we they can't tell us what it does to us five years in the~~* |
| 815 | *~~future, until it starts popping up five years in the future. So that's the biggest worry.~~*~~”~~ |
| 816 | ~~At the interpersonal level, biological, behavioral, and healthcare systems were the domains~~ |
| 817 | ~~of influence that emerged as the themes respondents utilized. One participant noted that their~~ |
| 818 | ~~mother and grandmother’s health impacted their decision not to stay in the same house as them,~~ |
| 819 | ~~thereby reducing the chance of infecting them with COVID-19. The participant stated that their~~ |
| 820 | ~~behavior to take the vaccine would be influenced by requirements from school. At the interpersonal~~ |
| 821 | ~~level of influence, multiple participants were present in the healthcare system domain of influence.~~ |
| 822 | ~~They noted the likelihood of going to their doctors depending on whether they had a friend who~~ |
| 823 | ~~was a doctor and whether the doctor was well-known or a public figure.~~ |
| 824 | ~~Participants from the focus group also had themes representing the biological, behavioral,~~ |
| 825 | ~~and healthcare systems domains of influence at the community level. One participant using the~~ |
| 826 | ~~biological domain stated that they took safety measures not so much for themselves but for people~~ |
| 827 | ~~they were around, while the participant using the behavioral domain believes it is their~~ |
| 828 | ~~responsibility to the rest of the community. Lastly, one of the participants was more concerned~~ |

829 ~~about the vaccine being effective more how much it might cost or if they had insurance for the~~
830 ~~vaccine.~~
831 ~~The societal level of influence had focus group responses based on representing the~~
832 ~~behavioral and healthcare systems domains of influence. Since the participant at the behavioral~~
833 ~~level was entering the Army, where they knew they would be influenced to take the vaccine. The~~
834 ~~participant noted that taking the vaccine would depend on who people felt was most influential to~~
835 ~~them. In summary, the AA male group had a unique concern as it pertains to historical mistrust.~~
836 ~~For example, a focus group participant made a community about whether AA males were~~
837 ~~represented in the clinical trials. The participant stated,~~
838 *~~“So I don't know how much, how much testing was done on African American males~~*
839 *~~personally?” (p 53)~~*

840

841	**~~Table 4.~~** ~~Black male focus group participant findings mapped onto the NIMHD Health Disparities~~

842	~~framework (nFGD=2, nP=3)~~

843	**~~LEVELS OF INFLUENCE~~**

|  | **~~Individual~~** | **~~Interpersonal~~** | **Community** | **~~Societal~~** |
| --- | --- | --- | --- | --- |
| ~~Biological~~ | ~~p57: “I have to stay on quite a bit~~ ~~because of my Nina. M~~**~~y Nina does~~ ~~have a chronic illness~~**~~” (Biological~~ ~~Vulnerability and Mechanisms)~~ | ~~p58:~~ **~~“It would be pretty bad because my mom~~**~~.~~ ~~She's with my grandma every week because she,~~ ~~she lives by herself. Now, which is pretty old. So,~~ ~~they probably honestly probably I’d leave me get~~ ~~stay somewhere.” (Family Microbiome / Caregiver-Child Interaction)~~ | p53: “I think I'm helping enough is more so not even for me, **it's probably for the people I'm around.”** (Community ~~Illness)~~ |  |
| ~~Behavioral~~ | ~~p58: “Totally take it. How is you know~~ ~~something… do…like~~ **~~we don't know~~ ~~how it's gonna affect us~~**~~… just came~~ ~~out.~~ **~~So, I guess there's only really one~~ ~~way to find ou~~**~~t, you know.” (Health~~ ~~Behaviors or Medical Decision Making)~~ | ~~P53: “I think, is probably about two years ago,~~ ~~probably when I came to college or about three~~ ~~years ago. And that was probably for like the~~ ~~tetanus and all the different things you have to do~~ ~~before you come to school. I feel like it's just my~~ ~~responsibility to the rest of the community.”~~ ~~(School/work functioning)~~ | p53**: “I feel like it's just my responsibility to the rest of the community**. (Community Functioning) | ~~p57: "But yeah, I~~ **~~think into the~~ ~~army on January 4, so I'm gonna~~ ~~pretty sure I'm going to get all~~ ~~those again.~~** ~~“~~  ~~(Policies and Laws)~~ |
| ~~Physical/Built Environment~~ |  |  |  |  |
| ~~Sociocultural Environment~~ |  |  |  |  |
| ~~Health Care Systems~~ |  | ~~p53~~**~~: “Probably if I had a friend that was a~~ ~~Doctor.~~**~~” (Patient-Clinician Relationship??)~~  ~~p57~~**~~: “Yeah, respected well known docto~~**~~r.”~~ ~~(Quality of Care)~~ | p53: “I will say the location you know where, where I would have to go to get it. **You know, I'm not too concerned about the pricing, the insurance**, but just where, **where it's most effective**.” ~~(Quality of Care)~~ | ~~p57:~~ **~~Yeah, whoever they feel is~~ ~~the most influential to them.~~ ~~They would agree with whatever~~ ~~they say.~~** ~~(Health Care Policies)~~ |
| **~~Health Outcomes~~** | **~~Individual Health~~** | **~~Family/Organizational~~**  **~~Health~~** | **Community**  **~~Health~~** | **~~Population~~**  **~~Health~~** |

844

845

846 ~~Black Mixed Gender Focus Group~~
847 **~~Table 5~~** ~~displays the findings from two mixed gender focus groups of Black participants.~~
848 ~~At the individual level, participants expressed heightened concern about their biological~~
849 ~~vulnerabilities, including age and pre-existing conditions. There was a fear expressed among~~
850 ~~participants that contracting COVID-19 could result in severe illness, hospitalization, or death.~~
851 ~~The fear led to self-protective behaviors.~~
852 ~~Respondents said they avoided public malls and family gatherings, wore gloves, and~~
853 ~~sprayed their shoes. The transition of germs to their physical/built environment was a concern, so~~
854 ~~a respondent stated that they did not want germs in their car, so they made up strategies to prevent~~
855 ~~the spread of germs from their groceries. A participant said,~~
856 *~~“I don't go out to the malls and family gatherings and stuff where there's a lot of people,~~*
857 *~~so therefore I go out, mainly when there's an essential reason and I try to do all those~~*
858 *~~things. So to distance myself I use a mask and gloves if necessary. Then I pull them off and~~*
859 *~~dispose of those gloves there at the store or where I am.I don't want any germs in my car.~~*
860 *~~And when I pick up my groceries, I call them and pick them up from the store. I have to put~~*
861 *~~it in place into my trunk because again I want nothing near me.” (p32)~~*
862 ~~For the interpersonal domain, focus group participants expressed the responsibility to~~
863 ~~protect loved ones, especially vulnerable family members such as grandchildren or children with~~
864 ~~pre-existing health conditions. Participants believed it was the responsibility of the people to~~
865 ~~protect their loved ones and to make sure that their other members were taking the necessary~~
866 ~~precautions to protect others. Family and peers were used as a source of information about the~~
867 ~~vaccine. A respondent mentioned that they would speak to their friends with science backgrounds~~
868 ~~to determine what is best for the community.~~
869 ~~At the societal level, structural factors such as discrimination, historical mistrust of the~~
870 ~~medical system, and political influence were cited as major concerns. Discrimination was a~~
871 ~~common theme in vaccine decision-making. Respondents gave statements about how the AA~~
872 ~~community had adverse outcomes and were cautious because there is a history. They wanted to~~
873 ~~know how many AAs the vaccine was tested on and what the results were. Political influences on~~
874 ~~public health also eroded trust in federal health agencies like the Centers for Disease Control and~~
875 ~~the National Institute of Health. However, local health leaders and departments were perceived as~~
876 ~~more trustworthy and less influenced by politics. A participant said,~~
877 *~~“Getting ready to get a little deep for a moment. Oh, the Tuskegee Syphilis Study done on~~*
878 *~~African American. I want to say with all my goodness, maybe the 20s, 30s, 40s. I don't~~*
879 *~~know the exact date, but that sticks in my mind, of giving People of color placebo … Oh, it~~*
880 *~~makes me cry sometimes because it's so sad… I am hesitant to go to the doctors. There’s~~*
881 *~~been [a] long line and a long history of African American people, whether we are older or~~*
882 *~~younger or female or male not being treated right in the health care system.” (p43)~~*
883 ~~A good portion of the focus group’s discussions fell under the healthcare system domain.~~
884 ~~Respondents spoke about limited knowledge about protecting oneself against COVID-19. The~~
885 ~~theme of quality of care also emerged, with respondents stating frustration with the Veterans~~
886 ~~Administration's inability to provide adequate services to veterans, requiring them to use private~~
887 ~~insurance to find more competent doctors. A common theme in their decision-making was the side~~
888 ~~effects and the testing population. Respondents expressed concerns about sensitivity to~~
889 ~~medications, knowing the side effects, the possibility of experiencing a side effect, and the need~~
890 ~~to know who the vaccine was tested on and how long. A participant said,~~
891 *~~“Medications are done on white people and maybe white men mostly. Yeah, just save it.~~*
892 *~~And so we are in the minority when they are testing out these new medications and then~~*
893 *~~they are being used on you. Used in a negative way. A lot of work is done in Caribbean~~*
894 *~~and African countries, and people are not well informed.” (p45)~~*
895 **~~Table 5.~~** ~~Black mixed gender focus group participant findings mapped onto the NIMHD Health Disparities~~
896 ~~framework (nFGD=2 groups, nP=9, 4)~~
897 **~~LEVELS OF INFLUENCE~~**

|  | **~~Individual~~** | **~~Interpersonal~~** | **~~Community~~** | **~~Societal~~** |
| --- | --- | --- | --- | --- |
| **~~Biological~~** | ~~p28:~~ **~~I think if I got it, I would be really, really sick because~~ ~~of my age and other risk factors~~**~~. I think I probably have to~~ ~~be put in the hospital and potentially, I could die as well.~~ ~~(Biological Vulnerability and Mechanisms)~~ | ~~p27: So,~~ **~~it's up to us to take care of ourselves and to~~ ~~protect ourselves and our families~~**~~, (Caregiver/Child~~ ~~Interaction)~~  ~~p29: So,~~ **~~I just try to protect my grandchildren and my~~ ~~daughter, who has a bad heart.~~** ~~I tried to pick those, you~~ ~~know, things like that. (Caregiver/Child Interaction)~~ |  |  |
| **~~Behavioral~~** | ~~p32:~~ **~~I don't go out to the malls and their family gatherings~~ ~~and stuff where there's a lot of people~~**~~, so therefore I go out,~~ ~~mainly when there's an essential reason and I try to do all those~~ ~~things. (Health Behaviors~~**~~)~~**  ~~p27: So,~~ **~~it's up to us to take care of ourselves and to protect~~ ~~ourselves and our families,~~** ~~but beyond that those things are~~ ~~beyond my control. So, I do what I can do to control. But~~ ~~beyond that, it's up to the Lord so that kind of where I'm~~ ~~coming. (Health Behaviors/ Coping Strategies)~~ | ~~p27: I do things to protect myself and I have to think~~ ~~about.~~ **~~I have a new grandson, and I do some~~ ~~babysitting for my family, so I don't want to get~~ ~~something to pass on to them.~~** ~~(Family Functioning)~~  ~~p44:~~ **~~I think that I kind of grew up in the generation of~~ ~~our parents did everything for us. So now we're adults,~~ ~~we don't know what we're doing. And I think it would~~ ~~cause a panic in my social circle because we don't~~ ~~know how to reach out for resources.~~** |  |  |
| **~~Physical/Built~~ ~~Environment~~** |  |  |  |  |
| **~~Sociocultural~~ ~~Environment~~** | ~~p27: [I] also have to say~~ **~~for people in the African American~~ ~~community, we've had some negative outcomes~~** ~~out of~~ ~~public health that were intended to be a help to society but~~ ~~worked out against us. …. those who are responsible for~~ ~~making sure that the vaccine is safe and available across~~ ~~populations, but especially in African American populations~~ ~~have as there is a lot of information out there that may not be~~ ~~always factual,~~ **~~but there's been a history and people are~~ ~~cautious~~**~~. (Response to Discrimination)~~ | ~~p32:~~ **~~I'll probably be discussing this with some of my~~ ~~some of my some of my friends, and particularly those~~ ~~that have a sciences background~~** ~~….making sure that~~ ~~I'm talking to people that have had to have a conversation~~ ~~so that we can talk and~~ **~~figure out which one would~~ ~~really be best for the community~~** ~~to possibly look at our~~ ~~us (Family/Peer Network )~~  ~~p43: I have been discriminated against in doctors’ offices~~ ~~in the settings or just because of the color of my skin. You~~ ~~know, it's not a story that I've heard or something that I'll~~ ~~read. Yes. What has happened for me personally.~~ ~~(Interpersonal Discrimination)~~ |  | ~~p28: I would want to know what…~~ **~~how many~~ ~~African Americans was it tested on~~** ~~and what~~ ~~would be outcomes, (Societal Structural~~ ~~Discrimination)~~  ~~p45: Yeah, I'm hoping my insurance will pay for~~ ~~winter, are they willing and, you know, the reality~~ ~~is, and elderly black woman. So, let's be on most~~ ~~of the totem pole are ready and believe it or not,~~ ~~there is racism in medical care system. (Societal~~ ~~Structural Discrimination)~~  ~~p27: African American populations have as there~~ ~~is a lot of information out there that may not be~~ ~~always factual,~~ **~~but there's been a history and~~ ~~people are cautious~~**~~. (Structural Discrimination)~~ |
|  |  | ~~p28:~~ **~~And what are the side effects. All medication~~ ~~seems to have side effects.~~** ~~So, I’ll probably be quite~~ ~~stable. Okay, so what are the side effects. (Medical~~ ~~Decision Making))~~  ~~P45: All the other medications we take in vaccines. We~~ ~~take take two years before they come out and I think this~~ ~~is being done under devious circumstances. And so, I~~ ~~would not take it. (medical decision making)~~ | p35: Well, only during the beginning of the virus, especially in New York. They had a hellacious time dealing with the virus and it finally got it in check to a, to an extent and just get the health care workers. That **they're overworked, as it is** and and I just feel like you just don't get the care that you would normally get if the virus was in upon us, you know. ~~(Availability of Services)~~ | ~~p28: What I mean this I don't know who was it~~ ~~tested on how long was it tested. What was the,~~ **~~what was the outcome of the test. Now, why are~~ ~~you trying to give it to me now that you give it~~ ~~to your cousin?~~** ~~I mean so I don't know I think I~~ ~~would really be apprehensive. (Health Care~~ ~~Policies)~~  ~~p35: Most recently, I had a problem with my back~~ ~~and I went to three epidurals and I must have seen~~ ~~four doctors VA before I finally got sent out to see~~ ~~your doctor on the outside, and now I'm still going~~ ~~through the same things so I'm at a point where~~ **~~I'd~~ ~~rather just pay with my own insurance and just~~ ~~try to get a good competent doctor~~** ~~(Quality of~~ ~~Care)~~ |
| **~~Health Outcomes~~** | **~~Individual Health~~** | **~~Family/Organizational~~**  **~~Health~~** | **~~Community~~**  **~~Health~~** | **~~Population~~**  **~~Health~~** |

898

899 ~~White Female Focus Group~~
900 **~~Table 6~~** ~~displays the findings from two White female focus groups. The group had points~~
901 ~~in their discussions that focused on every domain, with findings at the individual, interpersonal,~~
902 ~~community, and societal levels of influence. Within the individual level, participants expressed~~
903 ~~significant concerns about their health conditions. Some respondents expressed that having an~~
904 ~~autoimmune disease or having a cardiac procedure impacts their vaccine decision-making.~~
905 ~~Participants in these groups mentioned carefully weighed the risks of vaccinations, sometimes~~
906 ~~opting to minimize vaccine uptake due to concerns about triggering immune responses.~~
907 ~~The interpersonal influence was the level most coded in these focus groups. Interpersonal~~
908 ~~relationships and concerns for family health deeply influence decisions about vaccination.~~
909 ~~Respondents frequently mentioned worries about transmitting the illness to family members,~~
910 ~~especially their grandparents. Respondents spoke about needing to receive the vaccine for work.~~
911 ~~Their medical decisions appeared to be based on whether they needed the vaccine to continue~~
912 ~~working. One respondent talked about needing the vaccine to continue working in the neonatal~~
913 ~~intensive care unit (NICU). Another influence on their medical decision making process was the~~
914 ~~family norms on taking vaccines. Respondents spoke about the parents deciding whether the~~
915 ~~family receives a vaccination and how emerging adults may struggle with determining~~
916 ~~independently because they are used to the family making that decision for them. A participant~~
917 ~~said,~~
918 *~~“Yeah, protect other people. I mean, I have a staff that is required to work right now and~~*
919 *~~I do deal with the public and I have two children with asthma at home. So I would definitely~~*
920 *~~get it, but my motivator is to protect them. Primarily, you know, even at whatever possible~~*
921 *~~risk to me, which I don't think there's so much of a risk is there might be a lack of efficacy~~*
922 *~~or a lack of long term benefit, probably, but I would definitely get it if it came out because~~*
923 *~~I am working in-In the public still and I feel like it's my social responsibility as a to not be~~*
924 *~~a vector, if possible.”(p9)~~*
925 ~~The intersection between interpersonal level and health care systems showed the~~
926 ~~importance of trust in health care professionals. Respondents utilized this level of influence by~~
927 ~~inferring that a good relationship with their primary doctor can influence medical decision-making.~~
928 ~~Some respondents said they trusted their doctor and their recommendations when it came to their~~
929 ~~patient-clinician relationship. Additionally, regarding their medical decision-making, a respondent~~
930 ~~expressed not feeling confident in the production and quality of the vaccine.~~
931 *~~“One of my concerns is because it has a profit motive, but also because it's a race right~~*
932 *~~now. Not just cutting corners, but just efficacy may not be measured as stringent as it~~*
933 *~~should be. And so the first generation vaccines may or may not be effective, but they also~~*
934 *~~probably [have] been run through as much rigorous testing in order to have a time frame~~*
935 *~~and that worries me.” (p8)~~*
936 ~~At the community level, respondents demonstrated an awareness of herd immunity and the~~
937 ~~collective responsibility to protect others. Respondents spoke about exposing themselves to~~
938 ~~everything to reach herd immunity or wearing masks to prevent the spread of the illness.~~
939 ~~Accountability for protecting the community was an important theme for this group. In the~~
940 ~~healthcare system domain at the community level, a respondent expressed issues with the~~
941 ~~availability of services and not being able to receive the vaccine despite wanting to because of the~~
942 ~~requirements at the time.~~
943 ~~The societal level of influence had themes about the policies and laws surrounding COVID-~~
944 ~~19, such as taking a vaccine to go on vacation and the quality of care. A respondent spoke about~~
945 ~~how easy the process was at a local pharmacy. A theme that emerged is the political involvement~~
946 ~~around the vaccine. There was a growing awareness of societal debates, mainly driven by social~~
947 ~~media, misinformation, and political rhetoric. Respondents mentioned things like the vaccine~~
948 ~~being completely politicized to help a presidential campaign and a high percentage of people~~
949 ~~making medical decisions based on conflicting information on social media, causing skepticism~~
950 ~~around the vaccine.~~
951 *~~“I'm more than a public safety issue and I know at this point. I don't know who to trust~~*
952 *~~because you have a doctor saying one thing and you have another doctor saying the~~*
953 *~~complete opposite. And so, it is so convoluted. I just don't even. I've sort of lost my trust in~~*
954 *~~medicine as I feel that people can be bought in or swayed or can be directed to say things~~*
955 *~~that are true, because we don't know. We don't know what's true [and] what's not true. So,~~*
956 *~~I mean, the hardest thing is knowing who to go to. “ (p16)~~*

957

958	**~~Table 6.~~** ~~White female focus group participant findings mapped onto the NIMHD Health~~

959	~~Disparities framework (nFGD=2, nP=4,5)~~

960	**~~Levels of Influence~~**

|  | **~~Individual~~** | **~~Interpersonal~~** | **~~Community~~** | **~~Societal~~** |
| --- | --- | --- | --- | --- |
| **~~Biological~~** | ~~p11:~~ **~~I have an autoimmune disease~~**~~, and there~~ ~~are millions of opinions about autoimmune~~ ~~diseases, but there's one school of thought that~~ ~~it's good to minimize vaccines and so I don't, I~~ ~~don't get shingles. (Biological Vulnerability and~~ ~~Mechanisms)~~  ~~p7:~~ **~~I'm going to have a cardiac procedure~~** ~~early October. I want to get the vaccine to give~~ ~~me the two weeks that I need ahead of time to~~ ~~make sure it's working. (Biological Vulnerability~~ ~~and Mechanisms)~~  ~~p18: I fear dying from it. Even though~~ **~~I'm like~~ ~~very young and healthy~~** ~~(Biological~~ ~~Vulnerability and Mechanisms)~~ | ~~p18: But that's like how I think about it like~~ **~~I don't want to~~ ~~get my parents sick. You know my grandmas are both 87~~ ~~they would die~~**~~. I don't want that to happen. So I think, you~~ ~~know, one of my friends. She,~~ **~~her mom had cancer, was on~~ ~~chemo…~~** ~~(Family Microbiome)~~  ~~p19:~~ **~~There were enough vulnerable people in my life that~~** ~~I don't, you know, need to get it and risk giving it to them.~~ ~~(Family Microbiome)~~ | p9: I'm a public librarian so **I've always kind of believed that you get your immunity by just exposing yourself to ~~everything~~** ~~(Herd Immunity)~~  p18: **I also fear, giving it to someone else who wouldn't be able to fight it off** and I agree. (Community Illness)  p8: Have you seen the ad on TV and it's people's faces and there's just words going across the screen it says **“Joe masks for Jerry and Jerry masks for Sue and Sue…**” like they show the cascade of that, you know, of course, to me, that's a ~~hugely smart message. (Herd Immunity)~~ |  |
| **~~Behavioral~~** | ~~p19: Sometimes~~ **~~it's more fear than logic that~~ ~~runs your decision making at that time~~** ~~(Health Behaviors/Coping Strategies)~~ | ~~p16: So, if a hospital said to me, [name of p]. You,~~ **~~you must~~ ~~get this to be employed here that would have a huge sway~~ ~~on me.~~** ~~I can't really think of anybody else in my life that~~ ~~would have that kind of power to make me get it. (Work~~ ~~Functioning)~~  ~~p19: So now~~ **~~you've got all of these people in their early 20s~~ ~~and his parents have decided not to vaccinate them.~~** ~~And~~ ~~they've got to go back and make their own decision whether~~ ~~they're gonna you know get vaccinated and now they've got~~ ~~this weird, you know, strange alternative information out~~ ~~there. (Family Functioning)~~ | p9: I mean, if, i**f they don't want to be responsible for harming their parents or their children** or somebody who's vulnerable that they know and vaccination is also for others ~~(Community Functioning).~~ | ~~p19: You know, we don't think of ourselves as being~~ ~~high risk, but I was getting ready to go on a vacation~~ ~~in the summer and, and this was when the COVID~~ ~~stuff was just presenting you know earlier and people~~ ~~were kind of going well~~ **~~they're not gonna let you on~~ ~~a cruise ship.~~** ~~If you have flu symptoms and they~~ ~~might think it's COVID so you better protect yourself~~ ~~from the flu and so I got it really late in the season for~~ ~~a flu shot that I figured it's better than nothing.~~ ~~(Policies and Laws)~~ |
| **~~Physical/Built~~ ~~Environment~~** |  | ~~p18: And I went to get the shot at a clinic - at like on campus~~ ~~where they had emailed me saying, I could go and get it. Then~~ ~~when I got there turns out it was only for full time employees.~~ ~~And like I was a GA, but I wasn't like enough of an employee.~~ ~~So, I was frustrated by that because taken time out of my day~~ ~~to do that.~~ ~~Work Environment).~~ | p10: I know that some effort has been made to have people get their blood pressure checked when they're at the barbershop and creative ideas like that (Community ~~Resources)~~ |  |
| **~~Sociocultural~~ ~~Environment~~** |  | ~~p16: I have friends that are very much against vaccinations~~ ~~because~~ **~~their kids have had really bad responses~~ ~~(~~**~~Family/Peer Norms)~~**~~.~~**  ~~p8:~~ **~~I have a mother still who's 87 years old and she's my~~ ~~best historical documentarian of what things were like for~~ ~~example like Measles~~** ~~(Family/Peer Network)~~ |  | ~~p19: It started with this autism thing about other~~ ~~British things, and anyway… I think younger people~~ ~~are more aware of the anti-vaccination. concept. I~~ ~~think a high percentage have, as Donald Trump said~~ ~~to somebody, ‘you drank the Kool Aid.’~~ **~~I think~~ ~~there's a high a high percentage of people that~~ ~~because of what they read in social media or~~ ~~whatever are skeptical or of vaccinations~~**~~, not from~~ ~~personal experience. But what they've read. (Societal~~ ~~Structure)~~ |
| **~~Health Care Systems~~** | ~~p9: I have to say that the information out there is~~ ~~intimidating and kind of daunting for non-scientist to understand, you know, to~~ ~~comprehend~~ | ~~p19:~~ **~~I would trust my doctor~~** ~~just because you know I would~~ ~~assume that he's already experienced, you know, having his~~ ~~patients go through this (Patient-Clinician Relationship)~~  ~~p10: Because there's a race to develop vaccines or a lot of~~ ~~them that might come into being and people may be~~ ~~questioning. Should I wait or should I try the first one~~ ~~(Medical Decision Making)~~ | About who will get it first. **And so, I may wish to get it and yet it's not available** because I'm not over a certain age or I don't have a certain pre-existing condition or something. So I could see that being a barrier (Availability of Services)  p7: And the fascinating thing is you see these signs all over to sign “Free flu vaccines” and when you call or you stop in. Oh, ~~we don't have them yet.~~  Well then, don't put the signs up I don't know it. I finally did find that the Safeway at Broadway and Campbell does have them. So, I'm cool with that but…You know, maybe people who see those signs and then try to get one and find out that ~~they're not available.’~~ | ~~p19: finally, after about 20 minutes I just said "I'll try~~ ~~CVS" because CVS is an express script primary~~ ~~provider. But it was a little frustrating. I gave him my~~ ~~Medicare card, which I think that's what's needed, not~~ ~~your part D, but he didn't and this was the pharmacist.~~ ~~Anyway, so then now~~ **~~two days ago I went to CVS~~ ~~and it was like a five-minute process and it was just~~ ~~fine.~~** ~~(Quality of Care)~~  ~~p10: They were trying to pin her to the table and she's~~ ~~got really scared and she was crying and wiggling and~~ ~~trying to get away, and the nurse just got tougher and~~ ~~tougher in her language until she said if you don't stop~~ ~~wiggling, I'm going to break your arm. And my~~ ~~daughter was finally stuff sobbing long enough to get~~ ~~her breath and talk to me.~~ |
| **~~Health Outcomes~~** | **~~Individual Health~~** | **~~Family/Organizational~~**  **~~Health~~** | **~~Community~~**  **~~Health~~** | **~~Population~~**  **~~Health~~** |

961

962

963 ~~White Male Focus Group~~
964 **~~Table 7~~** ~~displays the White male focus group participants coded findings. This group~~
965 ~~focused on the biological, behavioral, physical built environment, socio cultural environment, and~~
966 ~~health care system domains of influence at the individual, interpersonal, community, and societal~~
967 ~~levels of influence. A few participants expressed biological reasons at the individual level~~
968 ~~regarding concern over the pandemic. One participant reported that his father was recovering from~~
969 ~~cancer, and he felt the need to protect him. Another mentioned that he felt he could end up~~
970 ~~hospitalized if he were to contract COVID-19. Protecting family and children was mentioned as~~
971 ~~important to participants under both the biological and behavioral domains. One participant stated~~
972 ~~that their young daughter had respiratory issues, and another was concerned about passing the~~
973 ~~illness to his wife and grandkids.~~
974 *~~“So, I think I also will be- I don't feel like I would be, like, showing any symptoms, to be~~*
975 *~~honest, like I said, I do exercise regularly. Whether I go out for a run or like in my backyard,~~*
976 *~~like, at least for 30 minutes every day, and least five days a week. I try to be healthy, and~~*
977 *~~I've really never had any complications when it comes to my health.” (p22)~~*
978 ~~Participants among this group demonstrated behavioral reasons for their decision-making~~
979 ~~at the interpersonal, community, and societal level of influence. One participant shared concern~~
980 ~~about protecting the community while out in public and another reported that he and his family~~
981 ~~will follow the guidelines put in place. Another participant reported following policies and laws in~~
982 ~~the place of work as his decision-making process,~~
983 *~~“I think my risks of getting it are high. And I think I take precaution, and I am pretty~~*
984 *~~proactive on me taking care of myself. But even with me taking those steps and working,~~*
985 *~~for I have the privilege of working from home, I think I still- anytime you're in public, I~~*

| 986 | *~~think you're putting you, yourself at risk. So, I think there's a high probability of either~~* |
| --- | --- |
| 987 | *~~encountering someone who has COVID-19 or is either connected. So, I think- my~~* |
| 988 | *~~framework that I use is anytime you're out in public or outside you're at risk of getting it.”~~* |
| 989 | *~~(p25)~~* |
| 990 | ~~The physical built environment domain only came up once among this group with a~~ |
| 991 | ~~participant showing concern for his colleagues that taught at the university interacting with~~ |
| 992 | ~~students.~~ |
| 993 | ~~At the interpersonal level of influence, many participants listed issues related to the~~ |
| 994 | ~~sociocultural environmental domain as the driving force behind their decisions. They appeared~~ |
| 995 | ~~more likely to go to family or other relatives when it came to making decisions around the~~ |
| 996 | ~~pandemic. One participant reported that he will not let others influence his decision-making.~~ |
| 997 | ~~Another participant expressed differences between two social groups that they interact with when~~ |
| 998 | ~~it comes to decision-making. One group being scientists and the other being from the business~~ |
| 999 | ~~community:~~ |
| 1000 | *~~“I think for… from my perspective, I think we've kept our circle pretty tight. And I think~~* |
| 1001 | *~~we've kind of been- especially during these times- we've kept it... Well, I think, trust, trust,~~* |
| 1002 | *~~trust in each other. And I think we've been hanging out a lot with like-minded people that~~* |
| 1003 | *~~kind of see this in the same light. So, trust really has played a big part in who we spend~~* |
| 1004 | *~~time with, who we hang out [with]. So, I think that has been a huge part in our, in our~~* |
| 1005 | *~~circle.” (p25)~~* |
| 1006 | ~~Under the health care system domain, participants reported individual and interpersonal~~ |
| 1007 | ~~reasons for making their decisions during the pandemic. This focus group expressed not being too~~ |
| 1008 | ~~worried as they have insurance coverage. Understanding how vaccines work, and the possible~~ |

| 1009 | ~~long-term effects was also noted. One participant appeared to want a vaccine that would be 100%~~ |
| --- | --- |
| 1010 | ~~effective with no side effects. This group also emphasized trusting their personal doctor when it~~ |
| 1011 | ~~comes to making health-related decisions. As one participant said,~~ |
| 1012 | *~~“Yeah, I usually vaccinations already come with a mild risk, regardless of whatever they're~~* |
| 1013 | *~~formulated with. The concern I would have is, you know, are they- I understand that you~~* |
| 1014 | *~~are using a test subject of, you know, our test group of so many people- but what age groups~~* |
| 1015 | *~~have they actually, you know, done testing with?” (p21)~~* |
| 1016 | ~~Another participant mentioned,~~ |
| 1017 | *~~“I think mine would be similar and that I'd want to know the effects of it. Is it you know,~~* |
| 1018 | *~~something, you get the vaccine, but you still have, you know, some odd percentage or~~* |
| 1019 | *~~chance of being exposed to it and getting it? It may just be mild or? I just want to~~* |
| 1020 | *~~understand If I did get the vaccine, what does that mean as far as risk of exposure, and if I~~* |
| 1021 | *~~got sick, what that means.” (p20)~~* |
| 1022 |  |

1023

1024

**~~Table 7.~~** ~~White male focus group participant findings mapped onto the NIMHD Health Disparities~~ ~~framework (nFGD=1, nP=6)~~

1025

**~~LEVELS OF INFLUENCE~~**

|  | **~~Individual~~** | **~~Interpersonal~~** | **Community** | **~~Societal~~** |  |
| --- | --- | --- | --- | --- | --- |
| **~~Biological~~** | ~~p23:~~ **~~I have a father, who's recovering from cancer,~~ ~~who's in a situation~~** ~~that they need to do that so- and I'm~~ ~~still go around him. (Biological Vulnerability and~~  ~~Mechanisms)~~ | ~~p21:~~ **~~I have a daughter that's 14 months old,~~** ~~that has respiratory~~ ~~issues and the conversation started with, you know,~~ **~~her life is more~~ ~~important.~~** ~~(Caregiver-Child Interaction)~~ |  |  |  |
|  | ~~p25: Although~~ **~~I think I am healthy~~** ~~and able bodied~~**~~, I see~~ ~~myself in the hospital.~~** ~~(Biological Vulnerability and~~ ~~Mechanisms~~ | ~~p20: Two things that would worry me is: I know there's some long-term~~  ~~impacts that those would, you know, hearts and some of those things~~ ~~that I would be concerned about; and then~~ **~~the bigger concern would~~ ~~be passing it on to my wife and grandkids~~**~~, (Family Microbiome)~~ |  |  |  |
|  | ~~p20: I would say, I think personally I wouldn't be too~~ ~~worried.~~ **~~I feel like I'm relatively healthy.~~** ~~(Biological~~ ~~Vulnerability and Mechanisms)~~ |  |  |  |  |
| **~~Behavioral~~** |  | ~~p22: You know,~~ **~~my family and I here are following the guidelines,~~ ~~you know, pretty strictly.~~** ~~(Family/Peer Norms)~~ | p22: Because, you know, like you said, **it is a community thing** so like we need to make sure that if we were. like, at the grocery store, if we went out and we were in close contact with some people. You know, we need to track that. (Community ~~Functioning)~~ | ~~p23: Last vaccine I had was in the~~ ~~military, and~~ **~~that was going through~~ ~~a line just getting popped.~~** ~~(Policies~~ ~~and Laws/Work Environment)~~ |  |
| **~~Physical/Built~~ ~~Environment~~** |  | ~~p24: New cases in Tucson are being driven by the university student~~ ~~population, particularly the~~ **~~large student housing complexes~~**~~. And so~~ ~~I have concern for, for my colleagues who are teaching and interacting~~ ~~with those students. (School/Work Environment)~~ |  |  |  |
| **~~Sociocultural~~ ~~Environment~~** |  | ~~p23: Well, I've just kept my social circle where I've done it. And if~~ ~~they're not comfortable, they're not comfortable. I let them dictate how~~ ~~they want to interact with me. I deal with a whole lot of folks all over~~ ~~town and so we interact, one way or the other.~~ **~~So, I don't let pressure~~ ~~from other folks dictate how I'm going to function~~** ~~(Family/Peer~~ ~~Norms)~~ |  |  |  |
|  |  | ~~p25: I don't have a doctor, where our relationship…~~ **~~I don't have a~~ ~~primary care doctor~~**~~. So,~~ **~~I think the first thing I would do is~~ ~~honestly call my mom or my parents or somebody~~**~~, relatives within~~ ~~my family, and just kind of let them know. (Family/Peer Norms)~~ |  |  |  |
|  |  | ~~p24: I have a lot of- as a PhD in microbiology,~~ **~~I have a lot of scientists~~ ~~in my social circle~~**~~, but I'm also a business owner here in town that~~ ~~owns a brewery. And so, I have~~ **~~a lot of non-science folks~~** ~~who are in~~ ~~the business world. And I would say that there is a~~ **~~noticeable~~ ~~difference between those two groups of people.~~** ~~(Family/Peer Norms)~~ |  |  |  |
| **~~Health Care Systems~~** | ~~p23: I don't worry about that.~~ **~~My family is financially~~ ~~taken care of. We got insurance covering that.~~** ~~Everything. That would not be a problem. (Insurance~~  ~~Coverage)~~ | ~~p23:~~ **~~I'd first check with my docto~~**~~r, tell them what's going on, what~~ ~~was reported, and ask them for recommendations. (Patient-Clinician~~ ~~Relationship)~~ |  |  |  |
|  | ~~p21: The other issue is, you know,~~ **~~having some knowledge~~ ~~of vaccines, you know you need time to really understand~~ ~~what the long-term effect is~~** ~~and that's the part that makes~~ ~~me more nervous is, you know~~**~~, I really want something~~ ~~that's just going to be magic~~**~~. (Health Literacy)~~ | ~~p23:~~ **~~I trust my doctor~~**~~. So, I will follow him because I've got a~~  ~~relationship with him. So, he knows how I think. So, we'll go with that.~~ ~~(Patient-Clinician Relationship)~~ |  |  |  |
| **~~Health Outcomes~~** | **~~Individual Health~~** | **~~Family/Organizational~~**  **~~Health~~** | **Community**  **Health** | **~~Population~~**  **~~Health~~** |  |

1026

1027

1028

| 1029 | from: [https://www.who.int/news-room/spotlight/ten-threats-to-global-health-in-2019.](https://www.who.int/news-room/spotlight/ten-threats-to-global-health-in-2019) | | | | | |
| --- | --- | --- | --- | --- | --- | --- |
| 1030 | 2. Prevention CfDCa. Trends in United States COVID-19 Deaths, Emergency Department (ED) Visits, | | | | | |
| 1031 | and Test Positivity by Geographic Area: Center for Disease Control and Prevention; 2025. Available from: | | | | | |
| 1032 | [https://covid.cdc.gov/covid-data-tracker/?#trends_totaldeaths_select_00.](https://covid.cdc.gov/covid-data-tracker/?&trends_totaldeaths_select_00) | | | | | |
| 1033 | 3. Prevention CfDCa. CDC Museum Covid-19 Timeline: Center for Disease Control and Prevention; | | | | | |
| 1034 | 2025 | [cited | 2025]. | Available | from: | [https://www-cdc-](https://www-cdc-gov.translate.goog/museum/timeline/covid19.html?_x_tr_hl=id&_x_tr_pto=tc&_x_tr_sl=en&_x_tr_tl=id) |
| 1035 | [gov.translate.goog/museum/timeline/covid19.html?_x_tr_hl=id&_x_tr_pto=tc&_x_tr_sl=en&_x_tr_tl=id](https://www-cdc-gov.translate.goog/museum/timeline/covid19.html?_x_tr_hl=id&_x_tr_pto=tc&_x_tr_sl=en&_x_tr_tl=id) | | | | | |
| 1036 | . | | | | | |
| 1037 | 4. Ndugga N, Hill L, Artiga S, Haldar S. Latest data on COVID-19 vaccinations by race/ethnicity. Kais | | | | | |
| 1038 | Family Found. 2021. | | | | | |

1.	Organization WH. Top Ten Threats to Global Health in 2019WHO 2019 [cited 2025]. Available

**Formatted:** Font: 12 pt

**Formatted:** Font: 12 pt

1039	5.	Nikolovski J, Koldijk M, Weverling GJ, Spertus J, Turakhia M, Saxon L, et al. Factors indicating

| 1040 | intention to vaccinate with a COVID-19 vaccine among older US adults. PloS one. 2021;16(5):e0251963. |
| --- | --- |
| 1041 | 6. Gatwood J, McKnight M, Fiscus M, Hohmeier KC, Chisholm-Burns M. Factors influencing likelihood |
| 1042 | of COVID-19 vaccination: a survey of Tennessee adults. American Journal of Health-System Pharmacy. |
| 1043 | 2021;78(10):879-89. |
| 1044 | 7. Yasmin F, Najeeb H, Moeed A, Naeem U, Asghar MS, Chughtai NU, et al. COVID-19 vaccine |
| 1045 | hesitancy in the United States: a systematic review. Frontiers in public health. 2021;9:770985. |
| 1046 | 8. Christ C. Vaccine Prioritization in Arizona: Arizona Department of Health Services; 2020 [cited |
| 1047 | 2026]. Available from: [https://directorsblog.health.azdhs.gov/vaccine-prioritization-in-arizona/.](https://directorsblog.health.azdhs.gov/vaccine-prioritization-in-arizona/) |
| 1048 | 9. Current Status of COVID-19 Vaccines Rollout Nationwide: April 2021 Update for the Attorney |
| 1049 | General Community: National Assocation of Attorneys General; 2021 [cited 2026]. Available from: |
| 1050 | [https://www.naag.org/attorney-general-journal/current-status-of-covid-19-vaccines-rollout-](https://www.naag.org/attorney-general-journal/current-status-of-covid-19-vaccines-rollout-nationwide-april-2021-update-for-the-attorney-general-community/) |
| 1051 | [nationwide-april-2021-update-for-the-attorney-general-community/.](https://www.naag.org/attorney-general-journal/current-status-of-covid-19-vaccines-rollout-nationwide-april-2021-update-for-the-attorney-general-community/) |
| 1052 | 10. Kriss JL. COVID-19 vaccination coverage, by race and ethnicity—national immunization survey |
| 1053 | adult COVID module, United States, December 2020–November 2021. MMWR Morbidity and Mortality |
| 1054 | Weekly Report. 2022;71. |
| 1055 | 11. Sypher K. Southern u.s. includes some of the nation’s best and worst COVID-19 vaccination rates: |
| 1056 | here’s why 2921. Available from: [https://www.apmresearchlab.org/ten-across-covid-vaccine.](https://www.apmresearchlab.org/ten-across-covid-vaccine) |
| 1057 | 12. Beleche T, Ruhter J, Kolbe A, Marus J, Bush L, Sommers B. COVID-19 vaccine hesitancy: |
| 1058 | demographic factors, geographic patterns, and changes over time. Published online. 2021;27. |
| 1059 | 13. Soares P, Rocha JV, Moniz M, Gama A, Laires PA, Pedro AR, et al. Factors associated with COVID- |
| 1060 | 19 vaccine hesitancy. Vaccines. 2021;9(3):300. |
| 1061 | 14. Rathnayake D, Bannon F, Clarke M, Jayasinghe V, Udayanga V. Descriptive analysis of |
| 1062 | determinants leading to COVID-19 vaccine hesitancy of healthcare workers in Sri Lanka. Population |
| 1063 | Medicine. 2025;7(March):1-6. |
| 1064 | 15. Bambra C, Lynch J, Smith KE. The unequal pandemic: COVID-19 and health inequalities. The |
| 1065 | Unequal Pandemic: Policy press; 2021. |
| 1066 | 16. Charura D, Hill AP, Etherson ME. COVID-19 vaccine hesitancy, medical mistrust, and mattering in |
| 1067 | ethnically diverse communities. Journal of racial and ethnic health disparities. 2023;10(3):1518-25. |
| 1068 | 17. Savoia E, Piltch-Loeb R, Goldberg B, Miller-Idriss C, Hughes B, Montrond A, et al. Predictors of |
| 1069 | COVID-19 vaccine hesitancy: socio-demographics, co-morbidity, and past experience of racial |
| 1070 | discrimination. Vaccines. 2021;9(7):767. |
| 1071 | 18. Williams AM, Clayton HB, Singleton JA. Racial and ethnic disparities in COVID-19 vaccination |
| 1072 | coverage: the contribution of socioeconomic and demographic factors. American journal of preventive |
| 1073 | medicine. 2022;62(4):473-82. |
| 1074 | 19. Clarke V, Braun V. Thematic analysis. The journal of positive psychology. 2017;12(3):297-8. |
| 1075 | 20. Mantina NM, Block Ngaybe M, Johnson K, Velickovic S, Magrath P, Gerald LB, et al. Racial/ethnic |
| 1076 | disparities in influenza risk perception and vaccination intention among Pima County residents in Arizona. |
| 1077 | Human vaccines & immunotherapeutics. 2022;18(7):2154506. |
| 1078 | 21. Alvidrez J, Castille D, Laude-Sharp M, Rosario A, Tabor D. The national institute on minority health |
| 1079 | and health disparities research framework. American journal of public health. 2019;109(S1):S16-S20. |
| 1080 | 22. Oktay JS. Grounded theory: Oxford University Press; 2012. |
| 1081 | 23. O’Connor C, Joffe H. Intercoder reliability in qualitative research: Debates and practical guidelines. |
| 1082 | International journal of qualitative methods. 2020;19:1609406919899220. |
| 1083 | 24. Zimmerman T, Shiroma K, Fleischmann KR, Xie B, Jia C, Verma N, et al. Misinformation and COVID- |
| 1084 | 19 vaccine hesitancy. Vaccine. 2023;41(1):136-44. |
| 1085 | 25. Washington HA. Medical apartheid: The dark history of medical experimentation on Black |
| 1086 | Americans from colonial times to the present: Doubleday Books; 2006. |

1087

**Formatted:** Font: 12 pt

1088

1089

1090

1091

1092

1093

1094

1095

1096

1097

1098

1099

1100

1101

1102

1103

1104

1105

1106

1107

1108

26. Grier WH, Cobbs PM. Black rage: Wipf and Stock Publishers; 2000.
27. Vázquez E, Juturu P, Burroughs M, McMullin J, Cheney AM. Continuum of trauma: fear and mistrust of institutions in communities of color during the COVID-19 pandemic. Culture, Medicine, and Psychiatry. 2024;48(2):290-309.
28. Strully KW, Harrison TM, Pardo TA, Carleo-Evangelist J. Strategies to address COVID-19 vaccine hesitancy and mitigate health disparities in minority populations. Frontiers in Public Health. 2021;9:645268.
29. Casselman-Hontalas A, Adams-Santos D, Watkins-Hayes C. Discourses of distrust: how lack of trust in the US health-care system shaped COVID-19 vaccine hesitancy. RSF: The Russell Sage Foundation Journal of the Social Sciences. 2024;10(4):154-72.
30. Privor-Dumm L, King T. Community-based strategies to engage pastors can help address vaccine hesitancy and health disparities in black communities. Journal of health communication. 2020;25(10):827-30.
31. Betsch C, Bach Habersaat K, Deshevoi S, Heinemeier D, Briko N, Kostenko N, et al. Sample study protocol for adapting and translating the 5C scale to assess the psychological antecedents of vaccination. BMJ open. 2020;10(3):e034869.
32. Betsch C, Schmid P, Heinemeier D, Korn L, Holtmann C, Böhm R. Beyond confidence: Development of a measure assessing the 5C psychological antecedents of vaccination. PloS one. 2018;13(12):e0208601.
33. Webb Hooper M, Nápoles AM, Pérez-Stable EJ. No populations left behind: vaccine hesitancy and equitable diffusion of effective COVID-19 vaccines. Journal of General Internal Medicine. 2021;36(7):2130-3.

Response to Reviewers

April 23, 2026

**Title:** *COVID-19 Vaccine Confidence Among Adults of Pima County using the NIMHD Minority Health and Health Disparities Research Framework: A Qualitative Analysis*

Dear Editor and Reviewers,

We sincerely thank the Academic Editor and Reviewers for their thoughtful and constructive feedback on our manuscript. We appreciate the time and care taken to evaluate this work. We have carefully revised the manuscript in response to all comments and believe the paper has been substantially strengthened as a result.

Major revisions include: clarifying recruitment and eligibility procedures; improving demographic reporting and table presentation; revising the manuscript to clearly reflect that data were collected prior to COVID-19 vaccine rollout and therefore represent anticipated perceptions and intentions rather than actual vaccine uptake; expanding the description of qualitative rigor and coding procedures; strengthening the mapping of findings to the NIMHD Minority Health and Health Disparities Research Framework; tempering conclusions regarding subgroup

differences; improving discussion of the study’s contribution to existing literature; revising tables for readability; and moving supplementary tables to Supporting Information files.

Below we provide a detailed point-by-point response to each comment. All changes are reflected in the revised manuscript and tracked-changes version. Page and line numbers refer to the revised manuscript.

We are grateful to the Editor and Reviewers for their careful reading and constructive feedback. Their comments helped us improve the manuscript’s clarity, rigor, and contribution. We hope that the revisions have addressed all concerns and that the revised manuscript will now be suitable for publication in PLOS ONE.

Sincerely,

Maiya Block Ngaybe PhD, MPH On behalf of all authors

### Responses to Journal Requirements / Editor Comments

| **Comment** | **Response** |
| --- | --- |
| 1. Please ensure that your manuscript meets PLOS ONE's style requirements, including those for file naming. | Thank you. We carefully reviewed the manuscript and submission files for compliance with PLOS ONE style requirements, including formatting, file naming, tables, references, and supporting information labeling. We also reviewed the manuscript against the PLOS ONE formatting templates and revised the files accordingly. |
| 2. We note that the grant information you provided in the ‘Funding Information’ and ‘Financial Disclosure’ sections do not match. Please ensure correct grant numbers are provided. | Thank you for identifying this inconsistency. We have corrected and harmonized the funding information and financial disclosure statements across the submission system and manuscript files to ensure consistency, including the correct grant number for MBN’s support.  The revised funding statement now reads: *“This study was conducted using PM’s start-up funds. The Pima County Health Department provided some funds to aid data collection. The*  *content is solely the responsibility of the authors and does not necessarily represent the official views of the funders. MBN was supported by the National Institutes of Health, Fogarty International Center/ Global Health Emerging Scholars Fellowship (D43TW010540). There was no additional external funding received for this study. The content is solely the responsibility of the authors and does not necessarily represent the official views of the funders.”*  These revisions are reflected in the manuscript on page [24], lines [596-597]. |
| 3. Please state what role the funders took in the study. If the funders had no role, include the statement in the cover letter. | Thank you. We have included the requested funder role statement in the cover letter and revised disclosure materials.  The statement is:  *“The funders had no role in study design, data collection and analysis, decision to publish, or preparation of the manuscript.”* |
| 4. Please confirm whether the submission contains all raw data required to replicate the study and revise the Data Availability Statement accordingly. | Thank you for this important request. We revised the Data Availability Statement to more accurately reflect the nature of the qualitative data and the  journal’s data-sharing expectations.: |

|  | *“Since our data contain potentially identifying or sensitive patient information, we have provided our ethical review board information in case someone would like to request permission to access our data and can make a reasonable case for why they would need access to the data.*  *Contact:* [*vpr-irb@arizna.edu*](mailto:vpr-irb@arizna.edu).*”* |
| --- | --- |
| 5. Please include a new copy of Table 1–7 in your manuscript; the current table is difficult to read. | Thank you. We reformatted and replaced Tables 1–7 to improve readability and consistency with PLOS ONE table guidelines. |
| 6. Supplementary tables are included in the manuscript file. Please remove them and upload them as Supporting Information, with legends listed in the manuscript after the references. | Thank you. We removed the supplementary tables from the main manuscript and uploaded them separately as Supporting Information files. We also added corresponding legends after the references section in the manuscript, as requested. |
| 7. If reviewers recommended citations, evaluate whether they are relevant and should be cited. | Thank you. We reviewed all recommended areas for additional citation and revised the discussion to better situate our findings in the existing literature where appropriate. We added relevant citations  where they strengthened contextualization and interpretation. |
| 8. Please review the reference list for completeness and correctness, including any retracted articles. | Thank you. We carefully reviewed and revised the reference list for completeness, formatting consistency, and accuracy. We also checked the cited literature to ensure that no retracted references were included unintentionally. Any changes to the reference list are reflected in the  revised manuscript. |
| **Responses to Reviewer #1** | |
| Comment 1: Clarify sampling details and temper conclusions about racial/ethnic differences. Incomplete demographic reporting and small subgroup representation make structured racial/ethnic comparisons too strong. | Thank you for this important observation. We agree and have revised the manuscript to present subgroup findings more cautiously. We now clarify that demographic information was incomplete for a portion of participants and that some groups had small representation, limiting the interpretation of subgroup comparisons. We revised the relevant sections in the Results and Discussion to avoid overstatement and to frame these findings as observed patterns within this sample and focus group context, rather than  broader racial or ethnic group characteristics. See results and discussion sections. |

| Comment 2: Emphasize that findings reflect pre-rollout perceptions rather than actual vaccine behavior. | Thank you. We agree and revised the manuscript throughout to make clear that data were collected between August and December 2020, prior to vaccine rollout in the United States, and therefore reflect anticipated perceptions, concerns, and stated intentions under uncertainty rather than actual vaccine uptake behavior. We revised the  Methods and discussion section to make this clear. |
| --- | --- |
| Comment 3: Provide more transparency regarding qualitative rigor, especially given no member checking. Clarify how credibility and consistency across coders were maintained. | Thank you. We expanded the Methods section to provide greater detail on the steps used to support qualitative rigor and analytic consistency.  Specifically, we clarified coder training, the process for codebook development and refinement, how transcripts were independently reviewed, and how discrepancies were discussed and resolved through consensus among the research team. We also expanded the Limitations section to acknowledge that member checking was not performed and to explain this as a limitation  while describing other steps taken to support credibility and dependability. |
| Comment 4: Avoid overgeneralizing group distinctions. These should be described as observed patterns within specific focus groups. | Thank you. We agree and have revised the manuscript to avoid overgeneralized statements about racial and ethnic groups. The language now reflects that these were patterns observed within this purposive sample and focus group setting,  rather than definitive group-level characteristics. |
| Comment 5: Deepen the discussion to clarify contribution beyond existing literature. | Thank you for this helpful suggestion. We revised the Discussion to move beyond summary and more clearly articulate how this study contributes  to the literature. |
| Comment 6: Improve demographic clarity and editorial consistency. | Thank you. We revised the demographic reporting to improve clarity, including updating the participant characteristics table to present counts and percentages more transparently and noting where data were missing or not disclosed. We also reviewed the manuscript for minor editorial and  formatting inconsistencies, including reference formatting. |
| **Responses to Reviewer #2** | |
| Comment 1: Recruitment procedures and inclusion/exclusion criteria are insufficiently described. | Thank you. We expanded the Methods section to provide additional detail on recruitment  procedures, including how participants were identified and invited, the recruitment setting and |

|  | approach, and the inclusion criteria used for  participation. |
| --- | --- |
| Comment 2: Demographic reporting is incomplete and percentages do not total 100%. | Thank you. We have clarified in the methods section that we do not have consistent demographic information and the totals are not 100% because some participants belonged to multiple groups. |
| Comment 3: The study does not discuss thematic saturation. | Thank you, we did, but called it “data saturation” so adjusted the wording accordingly. |
| Comment 4: Analytic procedures, including intercoder reliability and resolution of disagreements, are not fully described. | Thank you. We revised the Methods section to better describe the analytic process, including how coding consistency was supported, and how coding disagreements were resolved through  iterative discussion and consensus. |
| Comment 5: Mapping of findings to the NIMHD framework domains is not clearly demonstrated. | Thank you. We strengthened the presentation of the NIMHD framework throughout the manuscript. Specifically, we revised the Results to more explicitly identify how themes align with NIMHD levels and domains, and we clarified this mapping in the text and tables/[figure, if added].  We also revised the Discussion to explain how the framework helped structure interpretation of multilevel determinants of vaccine confidence. |
| Comment 6: Data were collected before vaccine rollout, so findings reflect anticipated intentions rather than actual confidence or uptake. | Thank you. We agree and revised the manuscript throughout to clarify the temporal context of the data. We now consistently describe the findings as reflecting pre-rollout perceptions, anticipated  vaccine intentions, and early confidence concerns, rather than actual uptake behavior. |
| Comment 7: Data availability is unclear; de-identified qualitative data or excerpts should be clarified. | Thank you. We revised the Data Availability Statement to clarify what materials are available and, where applicable, the reasons full transcripts are not publicly shared due to confidentiality and re-identification concerns. We also ensured that representative excerpts and supporting materials are included as appropriate in the Appendices and  that the statement clearly explains the mechanism for accessing data if permissible. |
| Comment 8: Findings are largely descriptive. Greater analytic depth is needed, including explicit linkage of themes to the framework and illustrative participant quotations. | We agree with the reviewer that the original Results section was largely descriptive. To address this, we adjusted the results and added more interpretation. We also added a cross-domain  synthesis paragraph to clarify how biological vulnerability, behavioral risk management, |

|  | sociocultural trust, healthcare system mistrust, and  political context interacted to shape vaccine intention. |
| --- | --- |
| Comment 9: Some interpretations overextend the data and should be tempered. | Thank you. We carefully reviewed the manuscript and revised interpretations that may have overstated the implications of the findings. We now use more cautious language in the Abstract, Results, Discussion, and Conclusion, particularly regarding subgroup comparisons and implications  for behavior. |
| Comment 10: The discussion could better situate findings in existing vaccine hesitancy literature. | Thank you. We expanded the Discussion to better contextualize our findings within prior literature on vaccine confidence, mistrust. This helps clarify both what is consistent with prior studies and what is distinctive about our findings. |
| **Responses to Reviewer #3** | |
| Comment 1: Four independent coders are mentioned, but no inter-rater reliability metric is reported. | Thank you for raising this point. We revised the Methods section to more clearly describe our  coding process and the procedures used to support analytic consistency. |
| Comment 2: If focus groups were conducted in Spanish, translation verification or back-translation should be described. | Thank you. We did not have funding for translation verification or back-translation due to the funding limitations for this research, which is why we do not report it. We further acknowledge in the Limitations that formal back-translation was  not conducted, which may have affected nuance in interpretation. |
| Comment 3: Ethical approval and informed consent should appear earlier in the Methods, including IRB protocol number. | Thank you. We revised the Methods section so that ethical approval and informed consent are presented clearly and prominently at the beginning of the Methods. The IRB protocol number is  provided. |
| Comment 4: Member checking was not performed, which weakens rigor. | Thank you. We agree that the absence of member checking should be clearly acknowledged. We retained this as a limitation and expanded the manuscript to describe alternative strategies used to support credibility, including multiple coders, iterative discussion, codebook refinement, and team consensus. We also clarify the practical reasons member checking was not feasible in this  study. |
| Comment 5: Percentages are reported without raw numbers, and denominator inconsistency makes interpretation  difficult. | Thank you. We have clarified in the methods section that we do not have consistent demographic information and the totals are not |

|  | 100% because some participants belonged to  multiple groups. |
| --- | --- |
| Comment 6: Very small subgroup representation limits the ability to draw thematic conclusions for those groups. | Thank you. We agree and have tempered the manuscript accordingly. We revised the Abstract, Results, and Discussion to avoid implying broad generalizability from very small subgroup representation. We now describe these as sample-specific observations and explicitly note this  limitation. |
| Comment 7: The NIMHD framework is referenced, but the mapping to levels of influence is not explicit enough. | Thank you. We strengthened the framework application by explicitly explaining the mapping of themes to relevant NIMHD levels and domains  clearer in the Methods. |
| Comment 8: Virtual focus groups may introduce digital divide bias, which should be addressed as a selection bias. | Thank you for this important observation. We added discussion of this issue to the Limitations section. |
| Comment 9: Thematic overlap between codes may indicate insufficient discriminant validity. | Thank you. We appreciate this point. However, we do not believe there is thematic overlap, since each theme has distinct differences based on the framework and therefore do not believe this is an issue. We discussed the definition of the overlap and came up with specific inclusion criteria during  our iterative discussions to ensure validity. |
| Comment 10: Presence of a stray number such as “940” suggests manuscript cleanup issues. | Thank you for identifying this. We carefully reviewed the manuscript and removed stray metadata, formatting artifacts, and versioning remnants to ensure a clean revised submission. |
