# Supplementary material for: COVID-19 vaccine confidence among adults of pima county using the NIMHD minority health and health disparities research framework: A qualitative analysis
Source: PLoS One. 2026 Jul 22;21(7):e0353345. doi: 10.1371/journal.pone.0353345 (PMC13390816; doi:10.1371/journal.pone.0353345)
Supplement: Peer review history

# Peer review: Decision Letter 0

**DOI:** 10.1371/journal.pone.0353345.r001

**Authors:** Mohamed Abousenna

2 Mar 2026

PONE-D-25-63283

COVID-19 Vaccine Confidence Among Adults of Pima County using the NIMHD Minority Health and Health Disparities Research Framework: A Qualitative Analysis

PLOS One

Dear Dr. Madhivanan,

Thank you for submitting your manuscript to PLOS ONE. After careful consideration, we feel that it has merit but does not fully meet PLOS ONE’s publication criteria as it currently stands. Therefore, we invite you to submit a revised version of the manuscript that addresses the points raised during the review process.

Please submit your revised manuscript by Apr 16 2026 11:59PM. If you will need more time than this to complete your revisions, please reply to this message or contact the journal office at plosone@plos.org. When you're ready to submit your revision, log on to https://www.editorialmanager.com/pone/ and select the 'Submissions Needing Revision' folder to locate your manuscript file.

Please include the following items when submitting your revised manuscript:

- A letter that responds to each point raised by the academic editor and reviewer(s). You should upload this letter as a separate file labeled 'Response to Reviewers'.
- A marked-up copy of your manuscript that highlights changes made to the original version. You should upload this as a separate file labeled 'Revised Manuscript with Track Changes'.
- An unmarked version of your revised paper without tracked changes. You should upload this as a separate file labeled 'Manuscript'.

-->If you would like to make changes to your financial disclosure, please include your updated statement in your cover letter. Guidelines for resubmitting your figure files are available below the reviewer comments at the end of this letter.

If applicable, we recommend that you deposit your laboratory protocols in protocols.io to enhance the reproducibility of your results. Protocols.io assigns your protocol its own identifier (DOI) so that it can be cited independently in the future. For instructions see: https://journals.plos.org/plosone/s/submission-guidelines#loc-laboratory-protocols. Additionally, PLOS ONE offers an option for publishing peer-reviewed Lab Protocol articles, which describe protocols hosted on protocols.io. Read more information on sharing protocols at https://plos.org/protocols?utm_medium=editorial-email&utm_source=authorletters&utm_campaign=protocols.

We look forward to receiving your revised manuscript.

Kind regards,

Mohamed Samy Abousenna, Ph.D

Academic Editor

PLOS One

Journal Requirements:

When submitting your revision, we need you to address these additional requirements.

1. Please ensure that your manuscript meets PLOS ONE's style requirements, including those for file naming. The PLOS ONE style templates can be found at

https://journals.plos.org/plosone/s/file?id=wjVg/PLOSOne_formatting_sample_main_body.pdf and

https://journals.plos.org/plosone/s/file?id=ba62/PLOSOne_formatting_sample_title_authors_affiliations.pdf

2.  We note that the grant information you provided in the ‘Funding Information’ and ‘Financial Disclosure’ sections do not match.

When you resubmit, please ensure that you provide the correct grant numbers for the awards you received for your study in the ‘Funding Information’ section.

3. Thank you for stating the following financial disclosure:

“This study was conducted using PM’s start-up funds. The Pima County Health Department provided some funds to aid data collection. The content is solely the responsibility of the authors and does not necessarily represent the official views of the funders. MBN was supported by the National Institutes of Health, Fogarty International Center/ Global Health Emerging Scholars Fellowship (D43TW010540). There was no additional external funding received for this study.”

Please state what role the funders took in the study. If the funders had no role, please state: "The funders had no role in study design, data collection and analysis, decision to publish, or preparation of the manuscript."

If this statement is not correct you must amend it as needed.

Please include this amended Role of Funder statement in your cover letter; we will change the online submission form on your behalf.

4. We note that your Data Availability Statement is currently as follows: All relevant data are within the manuscript and its Supporting Information files.

Please confirm at this time whether or not your submission contains all raw data required to replicate the results of your study. Authors must share the “minimal data set” for their submission. PLOS defines the minimal data set to consist of the data required to replicate all study findings reported in the article, as well as related metadata and methods (https://journals.plos.org/plosone/s/data-availability#loc-minimal-data-set-definition).

For example, authors should submit the following data:

- The values behind the means, standard deviations and other measures reported;

- The values used to build graphs;

- The points extracted from images for analysis.

Authors do not need to submit their entire data set if only a portion of the data was used in the reported study.

If your submission does not contain these data, please either upload them as Supporting Information files or deposit them to a stable, public repository and provide us with the relevant URLs, DOIs, or accession numbers. For a list of recommended repositories, please see https://journals.plos.org/plosone/s/recommended-repositories.

If there are ethical or legal restrictions on sharing a de-identified data set, please explain them in detail (e.g., data contain potentially sensitive information, data are owned by a third-party organization, etc.) and who has imposed them (e.g., an ethics committee). Please also provide contact information for a data access committee, ethics committee, or other institutional body to which data requests may be sent. If data are owned by a third party, please indicate how others may request data access.

5. Please include a new copy of Table 1-7 in your manuscript; the current table is difficult to read. Please follow the link for more information: https://journals.plos.org/plosone/s/tables

6. We notice that your supplementary tables are included in the manuscript file. Please remove them and upload them with the file type 'Supporting Information'. Please ensure that each Supporting Information file has a legend listed in the manuscript after the references list.

7. If the reviewer comments include a recommendation to cite specific previously published works, please review and evaluate these publications to determine whether they are relevant and should be cited. There is no requirement to cite these works unless the editor has indicated otherwise.

8. Please review your reference list to ensure that it is complete and correct. If you have cited papers that have been retracted, please include the rationale for doing so in the manuscript text, or remove these references and replace them with relevant current references. Any changes to the reference list should be mentioned in the rebuttal letter that accompanies your revised manuscript. If you need to cite a retracted article, indicate the article’s retracted status in the References list and also include a citation and full reference for the retraction notice.

[Note: HTML markup is below. Please do not edit.]

Reviewers' comments:

Reviewer's Responses to Questions

**Comments to the Author**

1. Is the manuscript technically sound, and do the data support the conclusions?

The manuscript must describe a technically sound piece of scientific research with data that supports the conclusions. Experiments must have been conducted rigorously, with appropriate controls, replication, and sample sizes. The conclusions must be drawn appropriately based on the data presented.

Reviewer #1: Partly

Reviewer #2: Partly

Reviewer #3: Yes

**********

2. Has the statistical analysis been performed appropriately and rigorously?

Reviewer #1: N/A

Reviewer #2: N/A

Reviewer #3: Yes

**********

3. Have the authors made all data underlying the findings in their manuscript fully available?

The PLOS Data policy requires authors to make all data underlying the findings described in their manuscript fully available without restriction, with rare exception (please refer to the Data Availability Statement in the manuscript PDF file). The data should be provided as part of the manuscript or its supporting information, or deposited to a public repository. For example, in addition to summary statistics, the data points behind means, medians and variance measures should be available. If there are restrictions on publicly sharing data—e.g. participant privacy or use of data from a third party—those must be specified.

Reviewer #1: Yes

Reviewer #2: No

Reviewer #3: Yes

**********

4. Is the manuscript presented in an intelligible fashion and written in standard English?

PLOS ONE does not copyedit accepted manuscripts, so the language in submitted articles must be clear, correct, and unambiguous. Any typographical or grammatical errors should be corrected at revision, so please note any specific errors here.

Reviewer #1: Yes

Reviewer #2: Yes

Reviewer #3: Yes

**********

5. Review Comments to the Author

Please use the space provided to explain your answers to the questions above. You may also include additional comments for the author, including concerns about dual publication, research ethics, or publication ethics. (Please upload your review as an attachment if it exceeds 20,000 characters)

Reviewer #1: Clarify sampling details and temper conclusions about racial/ethnic differences. The manuscript states, “Demographic information was not reported for almost half of the participants (21, 38%).” It also reports that only “9% were Black, and 4% were Asian,” yet presents structured racial/ethnic comparisons such as “Black participants distinctly highlighted systemic discrimination… Hispanic participants demonstrated strong community-centered trust, and White participants focused on confusion due to societal misinformation.” With incomplete demographic data and small subgroup representation, these comparisons should be framed more cautiously.

Emphasize that findings reflect pre-rollout perceptions rather than actual vaccine behavior.

The study notes recruitment occurred “between 22 Aug and 05 December, 2020” and that “The COVID-19 vaccination program was launched on December 14th, 2020, in the US.” Since data collection preceded vaccine availability, conclusions about “vaccination acceptance” should clearly reflect that participants were discussing anticipated decisions under uncertainty.

Provide more transparency regarding qualitative rigor. The authors acknowledge, “Transcripts were not returned to participants for comment or correction,” and “member checking was not performed due to the delay of the analysis post data collection.” Given these decisions, further clarification on how credibility and consistency across coders were maintained would strengthen methodological confidence.

Avoid overgeneralizing group distinctions. The manuscript states, “Black and Hispanic participants prominently emphasized familial and community biological vulnerabilities, contrasting with White participants, who focused more on individual health and robust immunity.” Given purposive sampling from team networks and incomplete demographic reporting, these distinctions should be described as observed patterns within specific focus groups rather than broader group characteristics.

Deepen the discussion to clarify contribution beyond existing literature. The discussion largely reiterates findings, for example, “Health literacy and misinformation were major concerns mentioned across groups,” and “Historical and sociopolitical mistrust were also common themes.” Expanding on how this study uniquely advances understanding within the NIMHD disparities framework would strengthen its scholarly contribution.

Improve demographic clarity and minor editorial consistency. The manuscript notes that participants were grouped by race/ethnicity and language, yet also states, “since they did not disclose their demographic information, we will not report it at an individual level.” Providing a clearer demographic breakdown table and carefully reviewing minor formatting inconsistencies in references would improve overall clarity.

Reviewer #2: This manuscript addresses an important public health topic, examining COVID-19 vaccine confidence among adults in Pima County using the NIMHD Minority Health and Health Disparities Research Framework. The study employs an appropriate qualitative design, and ethical approval and informed consent are clearly reported. The manuscript is written in clear and intelligible English.

However, there are several methodological and analytical weaknesses that need to be addressed to ensure the data fully support the conclusions:

Technical Soundness:

Recruitment procedures and inclusion/exclusion criteria are insufficiently described.

Demographic reporting is incomplete (percentages do not total 100%), which limits evaluation of sample representation.

The study does not discuss thematic saturation, making it unclear whether the sample size was sufficient.

Analytic procedures (intercoder reliability, resolution of coding disagreements) are not fully described.

Mapping of findings to the NIMHD framework domains is not clearly demonstrated.

Data were collected in Fall 2020, before COVID-19 vaccine rollout, so findings reflect anticipated vaccine intentions rather than actual confidence or uptake.

Data Availability:

While the manuscript states that all relevant data are included, it is unclear whether de-identified qualitative data (e.g., transcripts or excerpts) are publicly accessible. This is required to meet PLOS Data Policy.

Results and Discussion:

Findings are largely descriptive. Greater analytic depth is needed, including explicit linkage of themes to NIMHD framework levels and illustrative participant quotations.

Some interpretations overextend the data and should be tempered, particularly given the pre-vaccine timing.

The discussion could better situate findings in the context of existing literature on vaccine hesitancy.

Recommendations for Improvement:

Clarify recruitment strategies and inclusion/exclusion criteria.

Correct demographic reporting and describe thematic saturation.

Provide details on coder training, intercoder reliability, and conflict resolution.

Strengthen analytic depth and explicitly map findings to the NIMHD framework.

Clarify data availability and provide de-identified qualitative excerpts if possible.

Revise conclusions to reflect exploratory insights and avoid predictive claims.

Overall, the study has potential to contribute valuable insights into vaccine hesitancy among minority populations, but major revisions are required before it can be considered for publication.

Reviewer #3: 47–49 Missing Coder Reliability Mistake: You mention "four independent coders" but fail to report an Inter-rater Reliability (IRR) metric (e.g., Cohen’s Kappa or Percent Agreement).

Technical Impact: Without this, the reader cannot verify the consistency of the thematic analysis. This is a common "Major Revision" or "Rejection" point in qualitative research.

48 Transcription/Translation Gap Mistake: The line is physically broken, but technically it misses the Back-Translation verification step. If FGDs were conducted in Spanish, standard protocol requires a description of how translation accuracy was verified (e.g., back-translation by a third party).

50 Ethics Sequence Mistake: The mention of "Informed Consent" is tucked into the analysis description.

Technical Impact: Ethical approval and consent must be the first item in the Methods section, usually including the IRB Protocol Number, which is missing here.

472 Validation Failure Mistake: (From the back snippet/limitations) "Member checking was not performed."

Technical Impact: In qualitative studies, member checking (returning transcripts to participants) is a primary tool for Credibility (Internal Validity). Its absence significantly weakens the study's rigor.

53–54 Denominator Inconsistency Mistake: You report percentages (27%, 25%, 9%, 4%) for a sample size of N=56.

Technical Impact: These percentages result in non-whole numbers of people (e.g., 27% of 56 is 15.12). In clinical/social science reporting, if the denominator is under 100, you must report the raw number (n) alongside the percentage, or the math appears fabricated.

54 Under-representation Mistake: Reporting 4% for a racial group in a sample of 56 means only 2 people represented that entire category.

Technical Impact: This is statistically insufficient to draw thematic conclusions for that specific demographic, yet the abstract implies generalizable findings across these groups.

56 Missing NIMHD Mapping Mistake: You claim to use the NIMHD Framework, but the results snippet (Page 56) shows codes like "(Insurance Coverage)" and "(Health Literacy)" without mapping them to the specific Levels of Influence (Individual, Interpersonal, Community, Societal).

Back Snippet Selection Bias Mistake: The focus groups were conducted virtually (Line 46).

Technical Impact: This introduces a significant "Digital Divide" bias. Participants without stable internet or high health literacy (as mentioned in line p21) were likely excluded, yet the study aims to examine "health disparities." This contradiction must be addressed in the methodology as a selection bias.

Back Snippet Thematic Overlap Mistake: The codes "(Patient-Clinician Relationship)" and "(Health Literacy)" are applied to the same participant (p23).

Technical Impact: This suggests a lack of Mutual Exclusivity in your codebook. If codes overlap too much, the thematic analysis lacks "Discriminant Validity."

940 Metadata Leak Mistake: The presence of a stray number like "940" suggests the manuscript was generated from a database or old versioning software

Technical Impact: This signals to the editor that the "Clean Copy" protocol was not followed, often leading to a desk rejection for lack of professionalism.

**********

6. PLOS authors have the option to publish the peer review history of their article (what does this mean?). If published, this will include your full peer review and any attached files.

If you choose “no”, your identity will remain anonymous but your review may still be made public.

**Do you want your identity to be public for this peer review?**  For information about this choice, including consent withdrawal, please see our Privacy Policy.

Reviewer #1: No

Reviewer #2: No

Reviewer #3: **Yes:**  Safdar Ali

**********

[NOTE: If reviewer comments were submitted as an attachment file, they will be attached to this email and accessible via the submission site. Please log into your account, locate the manuscript record, and check for the action link "View Attachments". If this link does not appear, there are no attachment files.]

To ensure your figures meet our technical requirements, please review our figure guidelines: https://journals.plos.org/plosone/s/figures

You may also use PLOS’s free figure tool, NAAS, to help you prepare publication quality figures: https://journals.plos.org/plosone/s/figures#loc-tools-for-figure-preparation.

NAAS will assess whether your figures meet our technical requirements by comparing each figure against our figure specifications.

Attachment Submitted filename: Review File PONE-D-25-63283.docx

# Author comment: Author response to Decision Letter 1

**DOI:** 10.1371/journal.pone.0353345.r002

2 Jun 2026

Dear Editor and Reviewers,

We sincerely thank the Academic Editor and Reviewers for their thoughtful and constructive feedback on our manuscript. We appreciate the time and care taken to evaluate this work. We have carefully revised the manuscript in response to all comments and believe the paper has been substantially strengthened as a result.

Major revisions include: clarifying recruitment and eligibility procedures; improving demographic reporting and table presentation; revising the manuscript to clearly reflect that data were collected prior to COVID-19 vaccine rollout and therefore represent anticipated perceptions and intentions rather than actual vaccine uptake; expanding the description of qualitative rigor and coding procedures; strengthening the mapping of findings to the NIMHD Minority Health and Health Disparities Research Framework; tempering conclusions regarding subgroup differences; improving discussion of the study’s contribution to existing literature; revising tables for readability; and moving supplementary tables to Supporting Information files.

Below we provide a detailed point-by-point response to each comment. All changes are reflected in the revised manuscript and tracked-changes version. Page and line numbers refer to the revised manuscript.

We are grateful to the Editor and Reviewers for their careful reading and constructive feedback. Their comments helped us improve the manuscript’s clarity, rigor, and contribution. We hope that the revisions have addressed all concerns and that the revised manuscript will now be suitable for publication in PLOS ONE.

Sincerely,

Maiya Block Ngaybe PhD, MPH

On behalf of all authors

Responses to Journal Requirements / Editor Comments

Comment Response

1. Please ensure that your manuscript meets PLOS ONE's style requirements, including those for file naming. Thank you. We carefully reviewed the manuscript and submission files for compliance with PLOS ONE style requirements, including formatting, file naming, tables, references, and supporting information labeling. We also reviewed the manuscript against the PLOS ONE formatting templates and revised the files accordingly.

2. We note that the grant information you provided in the ‘Funding Information’ and ‘Financial Disclosure’ sections do not match. Please ensure correct grant numbers are provided. Thank you for identifying this inconsistency. We have corrected and harmonized the funding information and financial disclosure statements across the submission system and manuscript files to ensure consistency, including the correct grant number for MBN’s support.

The revised funding statement now reads:

“This study was conducted using PM’s start-up funds. The Pima County Health Department provided some funds to aid data collection. The content is solely the responsibility of the authors and does not necessarily represent the official views of the funders. MBN was supported by the National Institutes of Health, Fogarty International Center/ Global Health Emerging Scholars Fellowship (D43TW010540). There was no additional external funding received for this study. The content is solely the responsibility of the authors and does not necessarily represent the official views of the funders.”

These revisions are reflected in the manuscript on page [24], lines [596-597].

3. Please state what role the funders took in the study. If the funders had no role, include the statement in the cover letter.

Thank you. We have included the requested funder role statement in the cover letter and revised disclosure materials.

The statement is:

“The funders had no role in study design, data collection and analysis, decision to publish, or preparation of the manuscript.”

4. Please confirm whether the submission contains all raw data required to replicate the study and revise the Data Availability Statement accordingly. Thank you for this important request. We revised the Data Availability Statement to more accurately reflect the nature of the qualitative data and the journal’s data-sharing expectations.:

“Since our data contain potentially identifying or sensitive patient information, we have provided our ethical review board information in case someone would like to request permission to access our data and can make a reasonable case for why they would need access to the data. Contact: vpr-irb@arizna.edu.”

5. Please include a new copy of Table 1–7 in your manuscript; the current table is difficult to read.

Thank you. We reformatted and replaced Tables 1–7 to improve readability and consistency with PLOS ONE table guidelines.

6. Supplementary tables are included in the manuscript file. Please remove them and upload them as Supporting Information, with legends listed in the manuscript after the references.

Thank you. We removed the supplementary tables from the main manuscript and uploaded them separately as Supporting Information files. We also added corresponding legends after the references section in the manuscript, as requested.

7. If reviewers recommended citations, evaluate whether they are relevant and should be cited.

Thank you. We reviewed all recommended areas for additional citation and revised the discussion to better situate our findings in the existing literature where appropriate. We added relevant citations where they strengthened contextualization and interpretation.

8. Please review the reference list for completeness and correctness, including any retracted articles.

Thank you. We carefully reviewed and revised the reference list for completeness, formatting consistency, and accuracy. We also checked the cited literature to ensure that no retracted references were included unintentionally. Any changes to the reference list are reflected in the revised manuscript.

Responses to Reviewer #1

Comment 1: Clarify sampling details and temper conclusions about racial/ethnic differences. Incomplete demographic reporting and small subgroup representation make structured racial/ethnic comparisons too strong.

Thank you for this important observation. We agree and have revised the manuscript to present subgroup findings more cautiously. We now clarify that demographic information was incomplete for a portion of participants and that some groups had small representation, limiting the interpretation of subgroup comparisons. We revised the relevant sections in the Results and Discussion to avoid overstatement and to frame these findings as observed patterns within this sample and focus group context, rather than broader racial or ethnic group characteristics. See results and discussion sections.

Comment 2: Emphasize that findings reflect pre-rollout perceptions rather than actual vaccine behavior.

Thank you. We agree and revised the manuscript throughout to make clear that data were collected between August and December 2020, prior to vaccine rollout in the United States, and therefore reflect anticipated perceptions, concerns, and stated intentions under uncertainty rather than actual vaccine uptake behavior. We revised the Methods and discussion section to make this clear.

Comment 3: Provide more transparency regarding qualitative rigor, especially given no member checking. Clarify how credibility and consistency across coders were maintained.

Thank you. We expanded the Methods section to provide greater detail on the steps used to support qualitative rigor and analytic consistency. Specifically, we clarified coder training, the process for codebook development and refinement, how transcripts were independently reviewed, and how discrepancies were discussed and resolved through consensus among the research team. We also expanded the Limitations section to acknowledge that member checking was not performed and to explain this as a limitation while describing other steps taken to support credibility and dependability.

Comment 4: Avoid overgeneralizing group distinctions. These should be described as observed patterns within specific focus groups.

Thank you. We agree and have revised the manuscript to avoid overgeneralized statements about racial and ethnic groups. The language now reflects that these were patterns observed within this purposive sample and focus group setting, rather than definitive group-level characteristics.

Comment 5: Deepen the discussion to clarify contribution beyond existing literature. Thank you for this helpful suggestion. We revised the Discussion to move beyond summary and more clearly articulate how this study contributes to the literature.

Comment 6: Improve demographic clarity and editorial consistency.

Thank you. We revised the demographic reporting to improve clarity, including updating the participant characteristics table to present counts and percentages more transparently and noting where data were missing or not disclosed. We also reviewed the manuscript for minor editorial and formatting inconsistencies, including reference formatting.

Responses to Reviewer #2

Comment 1: Recruitment procedures and inclusion/exclusion criteria are insufficiently described. Thank you. We expanded the Methods section to provide additional detail on recruitment procedures, including how participants were identified and invited, the recruitment setting and approach, and the inclusion criteria used for participation.

Comment 2: Demographic reporting is incomplete and percentages do not total 100%.

Thank you. We have clarified in the methods section that we do not have consistent demographic information and the totals are not 100% because some participants belonged to multiple groups.

Comment 3: The study does not discuss thematic saturation.

Thank you, we did, but called it “data saturation” so adjusted the wording accordingly.

Comment 4: Analytic procedures, including intercoder reliability and resolution of disagreements, are not fully described.

Thank you. We revised the Methods section to better describe the analytic process, including how coding consistency was supported, and how coding disagreements were resolved through iterative discussion and consensus.

Comment 5: Mapping of findings to the NIMHD framework domains is not clearly demonstrated.

Thank you. We strengthened the presentation of the NIMHD framework throughout the manuscript. Specifically, we revised the Results to more explicitly identify how themes align with NIMHD levels and domains, and we clarified this mapping in the text and tables/[figure, if added]. We also revised the Discussion to explain how the framework helped structure interpretation of multilevel determinants of vaccine confidence.

Comment 6: Data were collected before vaccine rollout, so findings reflect anticipated intentions rather than actual confidence or uptake.

Thank you. We agree and revised the manuscript throughout to clarify the temporal context of the data. We now consistently describe the findings as reflecting pre-rollout perceptions, anticipated vaccine intentions, and early confidence concerns, rather than actual uptake behavior.

Comment 7: Data availability is unclear; de-identified qualitative data or excerpts should be clarified.

Thank you. We revised the Data Availability Statement to clarify what materials are available and, where applicable, the reasons full transcripts are not publicly shared due to confidentiality and re-identification concerns. We also ensured that representative excerpts and supporting materials are included as appropriate in the Appendices and that the statement clearly explains the mechanism for accessing data if permissible.

Comment 8: Findings are largely descriptive. Greater analytic depth is needed, including explicit linkage of themes to the framework and illustrative participant quotations.

We agree with the reviewer that the original Results section was largely descriptive. To address this, we adjusted the results and added more interpretation. We also added a cross-domain synthesis paragraph to clarify how biological vulnerability, behavioral risk management, sociocultural trust, healthcare system mistrust, and political context interacted to shape vaccine intention.

Comment 9: Some interpretations overextend the data and should be tempered.

Thank you. We carefully reviewed the manuscript and revised interpretations that may have overstated the implications of the findings. We now use more cautious language in the Abstract, Results, Discussion, and Conclusion, particularly regarding subgroup comparisons and implications for behavior.

Comment 10: The discussion could better situate findings in existing vaccine hesitancy literature.

Thank you. We expanded the Discussion to better contextualize our findings within prior literature on vaccine confidence, mistrust. This helps clarify both what is consistent with prior studies and what is distinctive about our findings.

Responses to Reviewer #3

Comment 1: Four independent coders are mentioned, but no inter-rater reliability metric is reported. Thank you for raising this point. We revised the Methods section to more clearly describe our coding process and the procedures used to support analytic consistency.

Comment 2: If focus groups were conducted in Spanish, translation verification or back-translation should be described.

Thank you. We did not have funding for translation verification or back-translation due to the funding limitations for this research, which is why we do not report it. We further acknowledge in the Limitations that formal back-translation was not conducted, which may have affected nuance in interpretation.

Comment 3: Ethical approval and informed consent should appear earlier in the Methods, including IRB protocol number. Thank you. We revised the Methods section so that ethical approval and informed consent are presented clearly and prominently at the beginning of the Methods. The IRB protocol number is provided.

Comment 4: Member checking was not performed, which weakens rigor.

Thank you. We agree that the absence of member checking should be clearly acknowledged. We retained this as a limitation and expanded the manuscript to describe alternative strategies used to support credibility, including multiple coders, iterative discussion, codebook refinement, and team consensus. We also clarify the practical reasons member checking was not feasible in this study.

Comment 5: Percentages are reported without raw numbers, and denominator inconsistency makes interpretation difficult.

Thank you. We have clarified in the methods section that we do not have consistent demographic information and the totals are not 100% because some participants belonged to multiple groups.

Comment 6: Very small subgroup representation limits the ability to draw thematic conclusions for those groups.

Thank you. We agree and have tempered the manuscript accordingly. We revised the Abstract, Results, and Discussion to avoid implying broad generalizability from very small subgroup representation. We now describe these as sample-specific observations and explicitly note this limitation.

Comment 7: The NIMHD framework is referenced, but the mapping to levels of influence is not explicit enough.

Thank you. We strengthened the framework application by explicitly explaining the mapping of themes to relevant NIMHD levels and domains clearer in the Methods.

Comment 8: Virtual focus groups may introduce digital divide bias, which should be addressed as a selection bias.

Thank you for this important observation. We added discussion of this issue to the Limitations section.

Comment 9: Thematic overlap between codes may indicate insufficient discriminant validity.

Thank you. We appreciate this point. However, we do not believe there is thematic overlap, since each theme has distinct differences based on the framework and therefore do not believe this is an issue. We discussed the definition of the overlap and came up with specific inclusion criteria during our iterative discussions to ensure validity.

Comment 10: Presence of a stray number such as “940” suggests manuscript cleanup issues.

Thank you for identifying this. We carefully reviewed the manuscript and removed stray metadata, formatting artifacts, and versioning remnants to ensure a clean revised submission.

Attachment Submitted filename: Cover Letter_NIMHD Paper Responses.docx

# Peer review: Decision Letter 1

**DOI:** 10.1371/journal.pone.0353345.r003

**Authors:** Mohamed Abousenna, Mohamed Abousenna

23 Jun 2026

COVID-19 Vaccine Confidence Among Adults of Pima County using the NIMHD Minority Health and Health Disparities Research Framework: A Qualitative Analysis

PONE-D-25-63283R1

Dear Dr. Madhivanan,

We’re pleased to inform you that your manuscript has been judged scientifically suitable for publication and will be formally accepted for publication once it meets all outstanding technical requirements.

Within one week, you’ll receive an e-mail detailing the required amendments. When these have been addressed, you’ll receive a formal acceptance letter and your manuscript will be scheduled for publication.

An invoice will be generated when your article is formally accepted. Please note, if your institution has a publishing partnership with PLOS and your article meets the relevant criteria, all or part of your publication costs will be covered. Please make sure your user information is up-to-date by logging into Editorial Manager at Editorial Manager® and clicking the ‘Update My Information' link at the top of the page. For questions related to billing, please contact billing support.

If your institution or institutions have a press office, please notify them about your upcoming paper to help maximize its impact. If they’ll be preparing press materials, please inform our press team as soon as possible -- no later than 48 hours after receiving the formal acceptance. Your manuscript will remain under strict press embargo until 2 pm Eastern Time on the date of publication. For more information, please contact onepress@plos.org.

Kind regards,

Mohamed Samy Abousenna, Ph.D

Academic Editor

PLOS One

Additional Editor Comments (optional):

Reviewers' comments:

Reviewer's Responses to Questions

**Comments to the Author**

1. If the authors have adequately addressed your comments raised in a previous round of review and you feel that this manuscript is now acceptable for publication, you may indicate that here to bypass the “Comments to the Author” section, enter your conflict of interest statement in the “Confidential to Editor” section, and submit your "Accept" recommendation.

Reviewer #2: All comments have been addressed

Reviewer #3: All comments have been addressed

**********

2. Is the manuscript technically sound, and do the data support the conclusions?

The manuscript must describe a technically sound piece of scientific research with data that supports the conclusions. Experiments must have been conducted rigorously, with appropriate controls, replication, and sample sizes. The conclusions must be drawn appropriately based on the data presented.

Reviewer #2: Yes

Reviewer #3: Yes

**********

3. Has the statistical analysis been performed appropriately and rigorously?

Reviewer #2: Yes

Reviewer #3: Yes

**********

4. Have the authors made all data underlying the findings in their manuscript fully available?

The PLOS Data policy requires authors to make all data underlying the findings described in their manuscript fully available without restriction, with rare exception (please refer to the Data Availability Statement in the manuscript PDF file). The data should be provided as part of the manuscript or its supporting information, or deposited to a public repository. For example, in addition to summary statistics, the data points behind means, medians and variance measures should be available. If there are restrictions on publicly sharing data—e.g. participant privacy or use of data from a third party—those must be specified.

Reviewer #2: Yes

Reviewer #3: Yes

**********

5. Is the manuscript presented in an intelligible fashion and written in standard English?

PLOS ONE does not copyedit accepted manuscripts, so the language in submitted articles must be clear, correct, and unambiguous. Any typographical or grammatical errors should be corrected at revision, so please note any specific errors here.

Reviewer #2: Yes

Reviewer #3: Yes

**********

6. Review Comments to the Author

Please use the space provided to explain your answers to the questions above. You may also include additional comments for the author, including concerns about dual publication, research ethics, or publication ethics. (Please upload your review as an attachment if it exceeds 20,000 characters)

Reviewer #2: Thank you for the revisions made in response to the reviewers' comments. The authors have adequately addressed the concerns raised during the previous review round. The manuscript has been improved substantially, the methodology is sound, the results support the conclusions, and the presentation is clear. In my opinion, the manuscript is suitable for publication in its current form.

Reviewer #3: All previous review comments are incorporated and track changes. No need to another changes in this article just accept the track changes

**********

7. PLOS authors have the option to publish the peer review history of their article (what does this mean?). If published, this will include your full peer review and any attached files.

If you choose “no”, your identity will remain anonymous but your review may still be made public.

**Do you want your identity to be public for this peer review?**  For information about this choice, including consent withdrawal, please see our Privacy Policy.

Reviewer #2: No

Reviewer #3: **Yes:**  safdar ali

**********

Attachment Submitted filename: PONE-D-25-63283_R1.docx

# Editor report: Acceptance letter

**DOI:** 10.1371/journal.pone.0353345.r004

**Authors:** Mohamed Abousenna, Mohamed Abousenna

PONE-D-25-63283R1

PLOS One

Dear Dr. Madhivanan,

I'm pleased to inform you that your manuscript has been deemed suitable for publication in PLOS One. Congratulations! Your manuscript is now being handed over to our production team.

At this stage, our production department will prepare your paper for publication. This includes ensuring the following:

* All references, tables, and figures are properly cited

* All relevant supporting information is included in the manuscript submission,

* There are no issues that prevent the paper from being properly typeset

You will receive further instructions from the production team, including instructions on how to review your proof when it is ready. Please keep in mind that we are working through a large volume of accepted articles, so please give us a few days to review your paper and let you know the next and final steps.

Lastly, if your institution or institutions have a press office, please let them know about your upcoming paper now to help maximize its impact. If they'll be preparing press materials, please inform our press team within the next 48 hours. Your manuscript will remain under strict press embargo until 2 pm Eastern Time on the date of publication. For more information, please contact onepress@plos.org.

You will receive an invoice from PLOS for your publication fee after your manuscript has reached the completed accept phase. If you receive an email requesting payment before acceptance or for any other service, this may be a phishing scheme. Learn how to identify phishing emails and protect your accounts at https://explore.plos.org/phishing.

If we can help with anything else, please email us at customercare@plos.org.

Thank you for submitting your work to PLOS ONE and supporting open access.

Kind regards,

PLOS ONE Editorial Office Staff

on behalf of

Dr. Mohamed Samy Abousenna

Academic Editor

PLOS One
